# Monoterpene-Containing Substituted Coumarins as Inhibitors of Respiratory Syncytial Virus (RSV) Replication

**DOI:** 10.3390/molecules26247493

**Published:** 2021-12-10

**Authors:** Tatyana M. Khomenko, Anna A. Shtro, Anastasia V. Galochkina, Yulia V. Nikolaeva, Galina D. Petukhova, Sophia S. Borisevich, Dina V. Korchagina, Konstantin P. Volcho, Nariman F. Salakhutdinov

**Affiliations:** 1 Department of Medicinal Chemistry, N.N. Vorozhtsov Novosibirsk Institute of Organic Chemistry, Acad. Lavrentjev Ave. 9, 630090 Novosibirsk, Russia; chomenko@nioch.nsc.ru (T.M.K.); korchaga@nioch.nsc.ru (D.V.K.); anvar@nioch.nsc.ru (N.F.S.); 2 Laboratory of Chemotherapy for Viral Infections, Smorodintsev Research Intitute of Influenza, Professor Popova Str., 15/17, 197376 St. Petersburg, Russia; anna.shtro@influenza.spb.ru (A.A.S.); nastyagalochkina@yandex.ru (A.V.G.); ulechka.s89@gmail.com (Y.V.N.); galina.petukhova@influenza.spb.ru (G.D.P.); 3 Laboratory of Physical Chemistry, Ufa Chemistry Institute of the Ufa Federal Research Center, 71 Octyabrya pr., 450054 Ufa, Russia; monrel@mail.ru

**Keywords:** coumarin, terpene, antiviral activity, cytotoxicity, respiratory syncytial virus, molecular modelling, F protein

## Abstract

Respiratory syncytial virus (RSV) is a critical cause of infant mortality. However, there are no vaccines and adequate drugs for its treatment. We showed, for the first time, that O-linked coumarin–monoterpene conjugates are effective RSV inhibitors. The most potent compounds are active against both RSV serotypes, A and B. According to the results of the time-of-addition experiment, the conjugates act at the early stages of virus cycle. Based on molecular modelling data, RSV F protein may be considered as a possible target.

## 1. Introduction

Respiratory syncytial virus (RSV), which belongs to the Pneumoviridae family, is an enveloped negative-sense RNA virus with two major serotypes, A and B [1]. RSV infects the respiratory tract, causing annual epidemics during the cold season. Despite a relatively low variability of this virus, the immunity to it is unstable, which may cause repeated infections of the same individual throughout life. It usually causes a cold. Patients under the age of 2 years, especially those born prematurely or with a heart condition, as well as the elderly, develop different symptoms of respiratory syncytial infection [2,3,4]. These age groups present with involvement of not only the upper respiratory tract, but the lower respiratory tract as well, and develop severe bronchiolitis and pneumonia, which can lead to death. RSV is the most common cause of bronchiolitis and pneumonia in children younger than one year of age [5]. According to a meta-analysis carried out in 2010, the number of children who may die from this disease is estimated to be about 199,000 annually [6]. RSV-associated childhood respiratory illness has become a challenge since the summer of 2021, when the number of cases increased sharply, which may have been due to the relaxation of COVID-19 quarantine measures [7]. There is no vaccine for RSV. Therapy for respiratory syncytial infection is usually symptomatic. The only treatment option is a non-specific and poorly effective antiviral agent: ribavirin [8,9]. In addition, the monoclonal antibody Palivizumab is approved for prophylaxis, but it is expensive and only moderately effective at reducing hospitalization rates.

**Figure 1:**
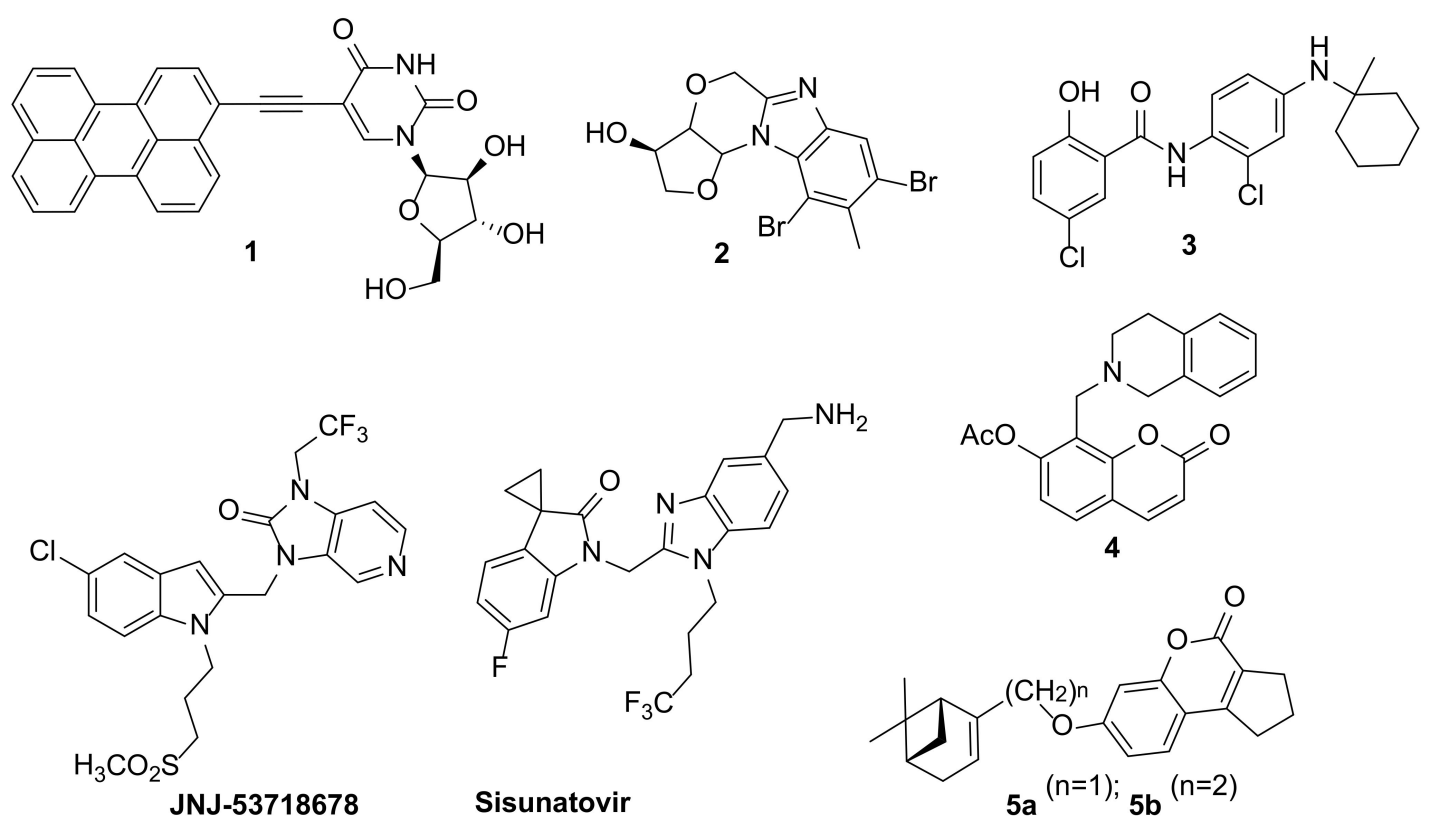
Known compounds with anti-RSV activity.

In recent decades, there has been significant progress in the identification of potential targets for RSV therapy and the search for low-molecular-weight inhibitors of RSV replication; the results of these studies were summarized in a review published in 2019 [10]. Of particular interest are new low-molecular-weight agents against RSV, such as (perylen-3-ylethynyl)uracil derivative **1** [11], furanoxazine-fused benzimidazole **2** [12], and benzamide **3** [13] (Figure 1), which inhibit RSV replication by acting on cellular targets, as well as JNJ-53718678 [14] and sisunatovir [15], which are under second phase of clinical trials and are highly effective inhibitors of the fusion (F) protein. The F protein is essential for viral entry into the host cell.

Many coumarin derivatives display a variety of biological activities [16,17,18,19,20], in particular antiviral activity [21,22,23,24,25,26,27]. Tetrahydroisoquinoline **4** is the only coumarin derivatives which exhibit anti-RSV activity [27]. Attachment of monoterpenoid fragments to parent molecules is known to significantly enhance their antiviral activity [28,29]. Recently, we have found that monoterpene-containing substituted 7-hydroxycoumarins effectively inhibit H1N1 influenza virus replication, with compound **5b** being most active [30]. Conjugate **5a** was found to exhibit the highest activity when added to infected cell culture at early stages of viral reproduction, probably due to the interaction with viral hemagglutinin. Replacement of the monoterpenoid moiety with a benzyl substituent led to a complete loss of antiviral activity. However, there are no data on RSV inhibitory activity of coumarin derivatives comprising a terpene moiety. In this study, we revealed the ability of monoterpene–coumarin conjugates to inhibit RSV replication and suggested a possible mechanism of their action.

## 2. Results and Discussion

### 2.1. Chemistry

Our earlier study on the activity of monoterpene–coumarin conjugates against the influenza virus revealed that the structure and absolute configuration of a monoterpene moiety and the size of an annulated aliphatic ring had a significant effect on the antiviral activity. In addition, as the length of an aliphatic bridge between monoterpene and coumarin moieties increases from one to two CH_2_-groups, the antiviral activity enhances [30]. Given these data, in this study we synthesized a library of compounds that included both previously obtained coumarin–monoterpenoid hybrids [30,31] and new compounds with coumarin and monoterpene bicyclic fragments separated from each other by three or four CH_2_-groups, as well as a number of nitrogen-containing coumarin derivatives with an NH_2_-group instead of an OH-group.

**Scheme 1:**
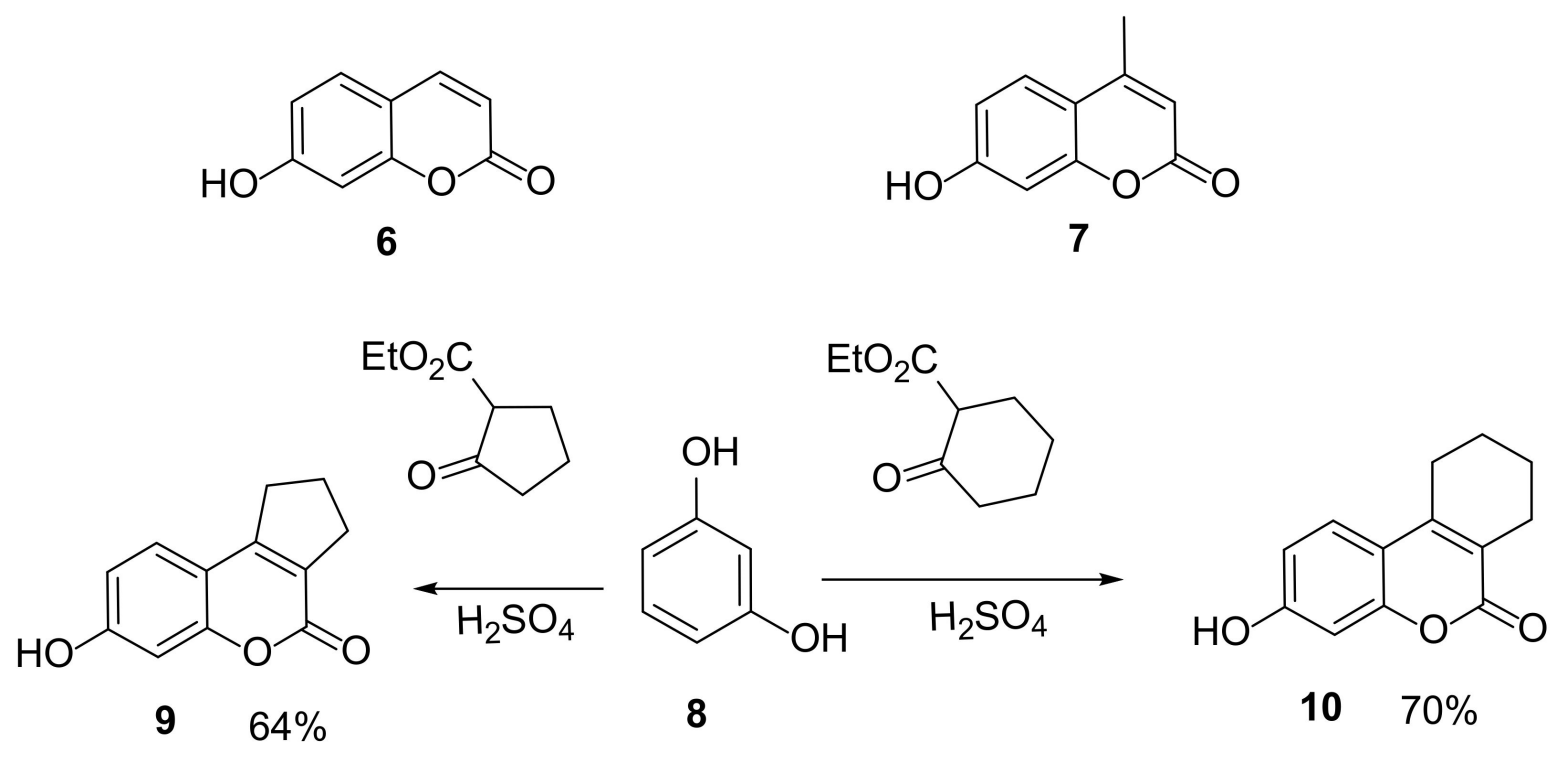
Synthesis of 7-hydroxycoumarins.

7-Hydroxycoumarin derivatives were prepared from commercially available umbelliferone **6** and 4-methyl-7-hydroxycoumarin **7**, as well as coumarins **9** and **10** (Scheme 1) synthesized from resorcinol **8** according to [31].

**Scheme 2:**
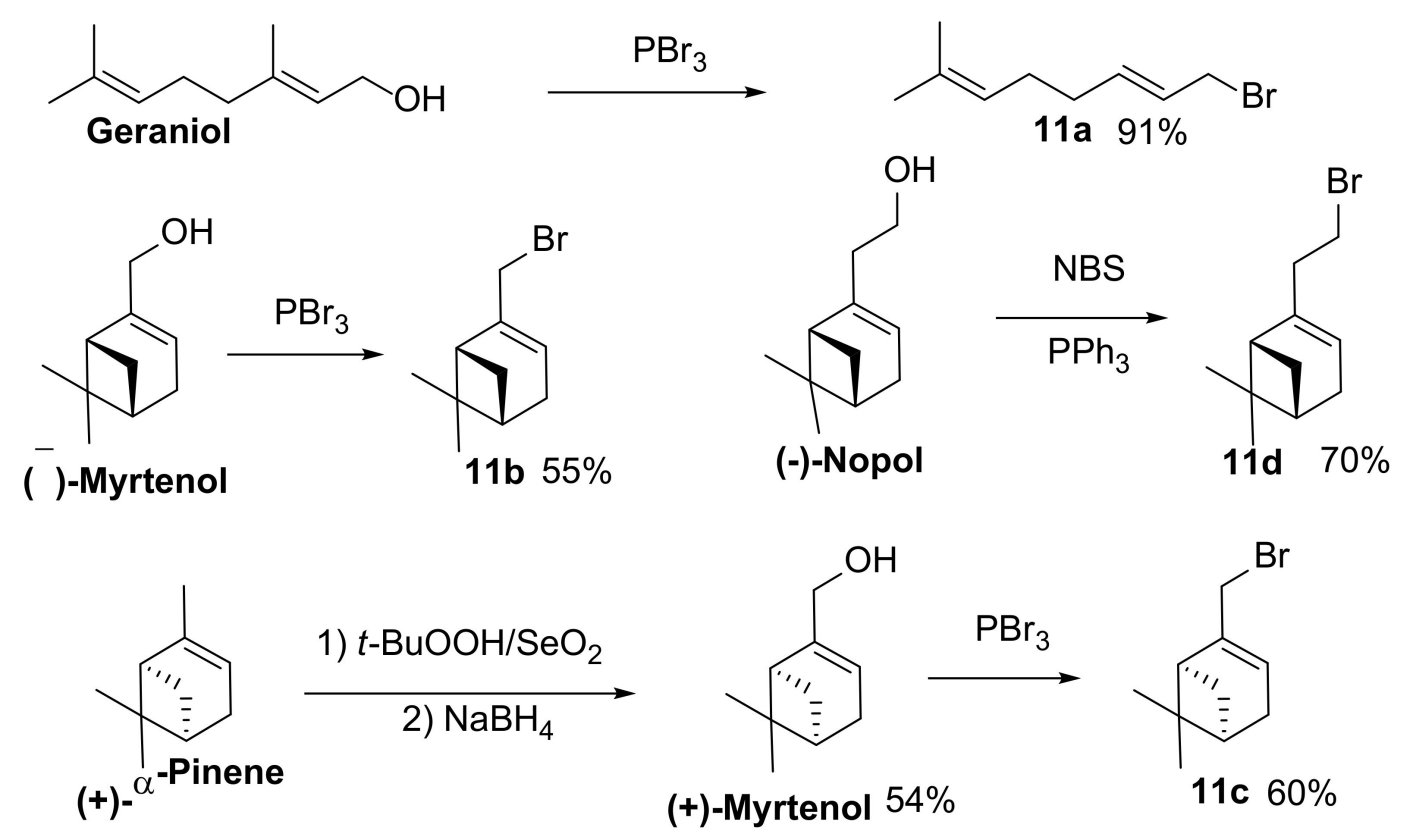
Synthesis of monoterpenoid bromides **11a**–**d**.

Bromides **11a**–**c** were synthesized in the reaction between appropriate alcohols and PBr_3_ in accordance with [31], using geraniol, (–)-myrtenol, and (+)-myrtenol produced from (+)-α-pinene as starting monoterpenoids (Scheme 2). Nopol bromide **11d** was previously obtained by the method presented in [31] in a low yield; therefore, the NBS/PPh_3_ system [32] was used to synthesize compound **11d**.

Derivatives of (–)-α-pinene and its homologue (–)-nopol exhibited the highest activity against the influenza virus; therefore, we synthesized compounds containing an extended aliphatic chain and an (–)-α-pinene moiety. A homolog of nopol bromide **11d**, which contained an additional CH_2_-group, was synthesized according to the following scheme: (+)-*Trans*-pinocarveol, which was prepared according to [33], reacted with triethyl orthoacetate in the presence of hexanoic acid to form ester **12**, which was then reduced by LiAlH_4_ to alcohol **13** [34]. Bromide **11e** was prepared by treating alcohol **13** with NBS/PPh_3_.

**Scheme 3:**
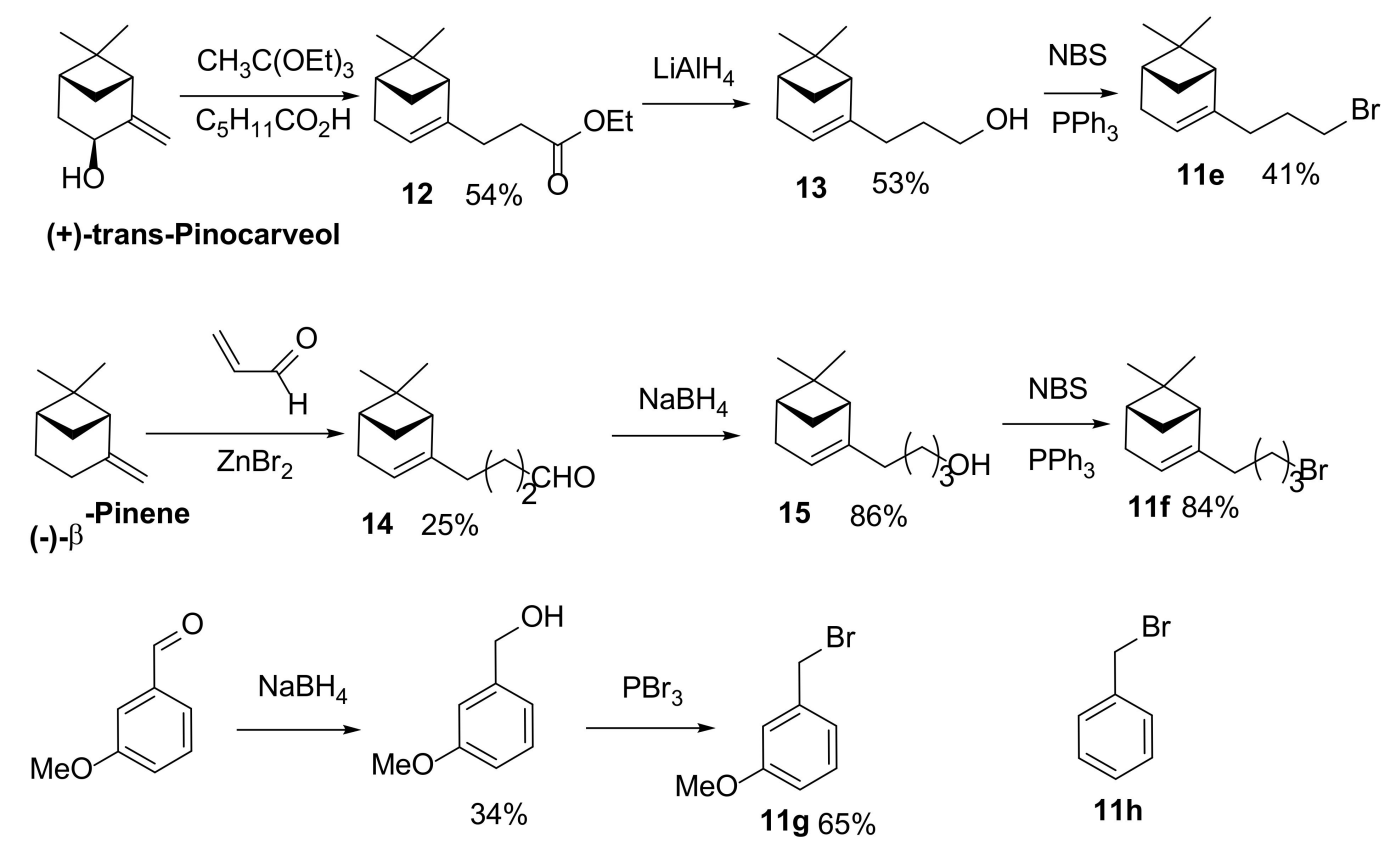
Synthesis of bromides **11e**–**h**.

Another nopol bromide homolog containing two additional CH_2_-groups, compound **11j**, was synthesized as follows (Scheme 3): (−)-β-Pinene reacted with acrolein in the presence of ZnBr_2_ to form aldehyde **14** (conversion, 55%; yield after chromatography, 25%), which was reduced by NaBH_4_ to alcohol **15** [35]. The interaction of alcohol **15** with NBS/PPh_3_ resulted in bromide **11f** [35]. 1-(Bromomethyl)-3-methoxybenzene **11g** was synthesized from 3-methoxybenzaldehyde; in addition, benzyl bromide **11h** was used.

**Scheme 4:**
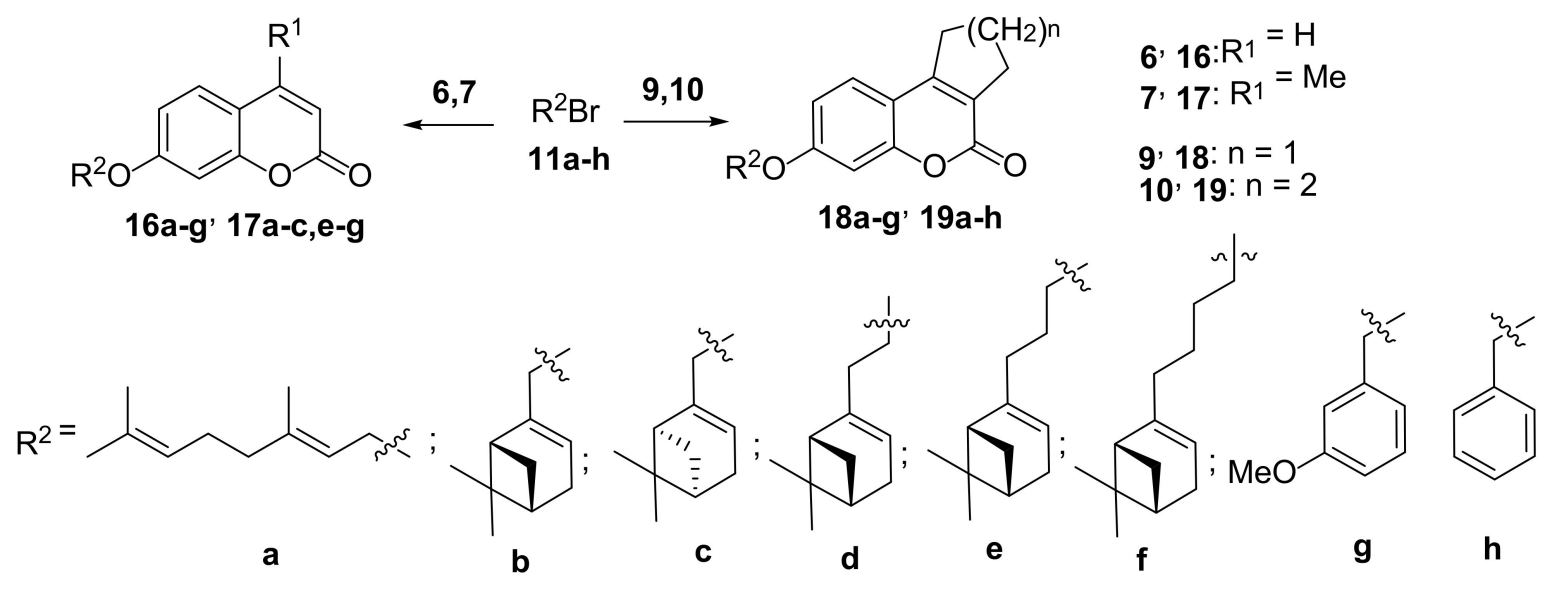
Synthesis of substituted coumarins **16**–**19**.

Aurapten **16a** and other coumarin derivatives **16**–**19** were prepared in the reaction of 7-hydroxycoumarins **6**–**10** with appropriate bromides **11** (Scheme 4), as described elsewhere [30]. The products were purified by recrystallization or column chromatography (yields 24–80%). The reaction of nopyl bromide **11d** with methylcoumarin **7** failed due to the formation of a complex reaction mixture with a high resinification level.

**Scheme 5:**
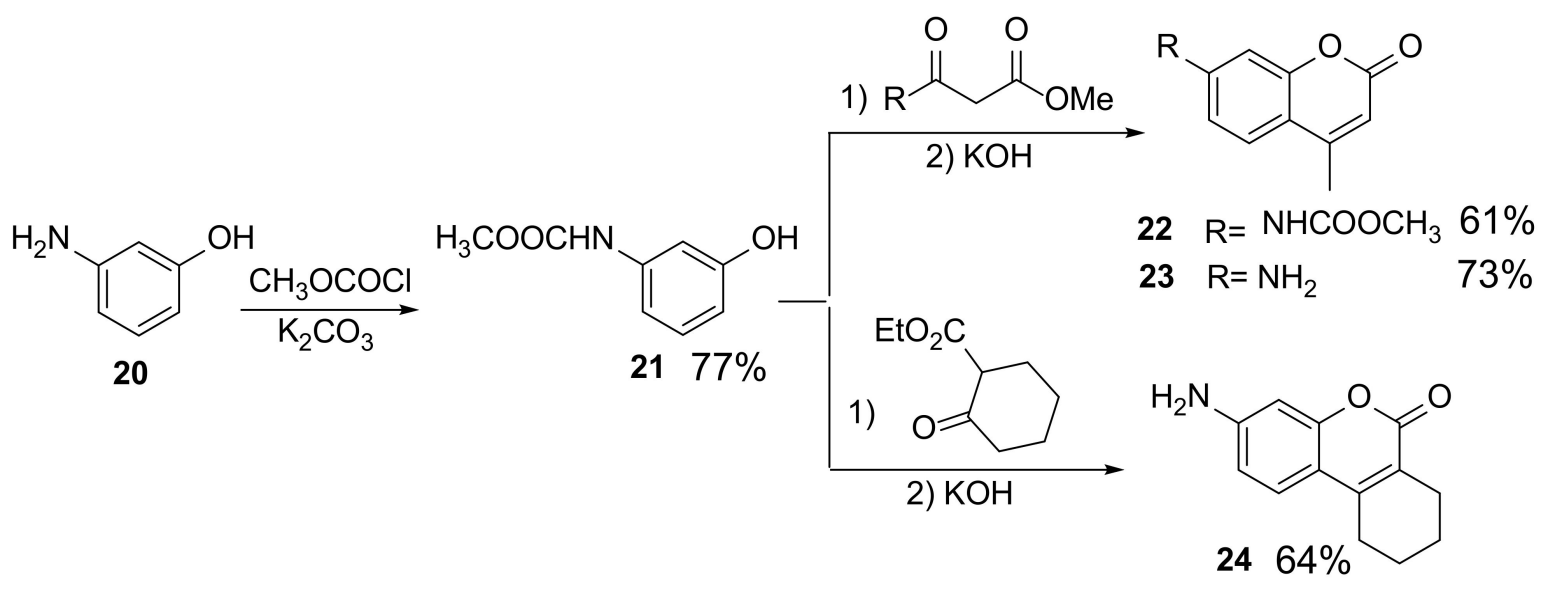
Synthesis of aminocoumarins **23** and **24**.

7-Aminocoumarins **23** and **24** were synthesized according to the method presented in [36], starting from 3-aminophenol **20**, via intermediate compound **21**, and its interaction with the appropriate ketoesters (Scheme 5).

**Scheme 6:**
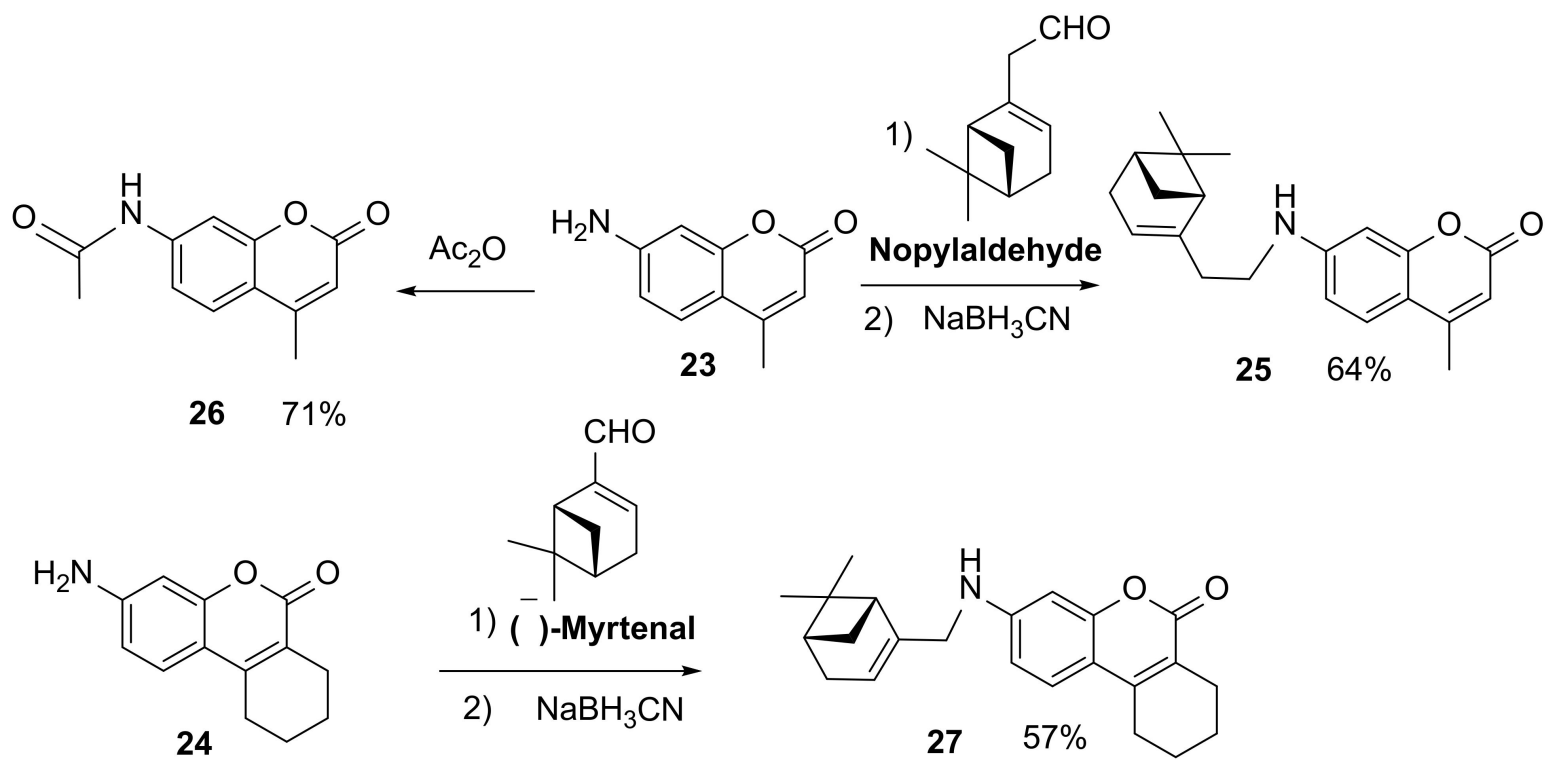
Synthesis of aminocoumarin derivatives **25**–**27**.

Furthermore, compound **23** reacted with (–)-nopol aldehyde, and on reduction with NaBH_3_CN, produced secondary amine **25**. In addition, acylation of amine **23** with acetic anhydride led to acetamide **26** [37]. Similarly to compound **25**, amine **27** was synthesized from amine **24** and (–)-myrtenal (Scheme 6).

### 2.2. Biology

#### 2.2.1. Cytotoxicity Test

The cytotoxicity was tested using a standard MTT-test (detailed below in Section 3.2.1). A series of 2-fold dilutions of compounds was made; then, each dilution was added to 24 h old cell monolayer, after which, cells were incubated for 24 h. Cell viability was assessed by adding the MTT solution. Based on the data obtained, the CC_50_ was calculated.

#### 2.2.2. Antiviral Activity

The antiviral activity against the respiratory syncytial virus was assessed by adding a series of 3-fold dilutions of test compounds, followed by addition of the virus in a series of 10-fold dilutions. Cells were incubated for 1 h; then, the virus was washed out and compounds were added again. Cell were incubated for 6 days, after which the viral presence was investigated, using the ELISA method.

The virus titer was calculated using the Reed and Muench method.

The obtained results are shown in Table 1. Compounds were considered promising with a selectivity index of 10 or higher.

At the first stage, all the prepared compounds were tested for their ability to inhibit the replication of RSV A. Aurapten **16a**, containing an acyclic monoterpene substituent, exhibited high activity against RSV A in the lower micromolar range, but its selectivity index was less than 8 due to high cytotoxicity. Among compounds **16b**,**c** containing an α-pinene fragment, only compound **16c**, derived from (+)-α-pinene, exhibited noticeable activity, with the selectivity index exceeding 11. Elongation of the hydrocarbon chain from **16b** to compounds **16d**–**f** led to a nonlinear change in the biological properties: high antiviral activity was exhibited by compounds with one (**16d**) and three (**16f**), but not two (**16e**) additional CH_2_-groups. Due to its lower cytotoxicity, compound **16f** displayed the best selectivity index, about 65, among monoterpene–coumarin conjugates of this type. Coumarin derivative **16g**, containing an aromatic substituent, showed a high selectivity index (30), which was related to its low cytotoxicity, rather than its high activity.

Among 4-methylcoumarin derivatives **17**, compound **17c**, containing an (+)-α-pinene moiety, exhibited high activity in the submicromolar range, whereas its (–)-isomer was 20-fold less active. Compound **17c** had a high selectivity index of 90. Elongation of the hydrocarbon chain (a compound with one additional CH_2_-group was not prepared due to the formation of a complex reaction mixture) did not affect the activity much; it was almost the same, similarly to toxicity. The less cytotoxic compound **17c** had a selectivity index of 62. Compound **17g**, containing an aromatic substituent, did not display significant antiviral activity.

Investigation of the biological properties of compound **18**, comprising a cyclopentane ring annulated with a coumarin moiety, showed that (+)-α-pinene derivative **18c** with a selectivity index of 100 exhibited the highest activity and moderate toxicity. The other compounds were either toxic (**18a**,**b**,**e**–**g**) or inactive (**18d**); only compound **18e** had a noticeable selectivity index of 18.

Among coumarin **19**, containing an annulated cyclohexane ring, the highest activity was exhibited by compounds **19a** and **19d**, which were derived from geraniol and nopol, respectively. High selectivity indices were also displayed by compounds **19c** and **19f**, which were both less active and less toxic. Coumarin derivative **19g**, containing a methoxybenzyl substituent, had a selectivity index of more than 10, which was due to its low cytotoxicity. Removal of the methoxy group from the aromatic ring in compound **19h** led to a sharp increase in its toxicity.

Amine **25** showed significant activity and moderate toxicity, which resulted in a selectivity index of more than 10. Transition to acetamide **26** led to a loss of the antiviral effect. Compound **27** was almost one order of magnitude more active and significantly more toxic than amine **25**, with the selectivity index being about 15. Compound **27** was much more active than its oxygen-containing analog **19b**, which substantiates further research regarding the synthesis of N-linked monoterpene–coumarin conjugates and the investigation of their anti-RSV activity.

A significant portion of the prepared coumarin derivatives were tested for their ability to inhibit RSV replication. Among the compounds tested, **16c,g**, **17c,f**, **18c**, **19a,c**,**d**, and **19f** had a selectivity index of more than 10, with the last two being most active. In general, activity of *O*-linked coumarin–monoterpene conjugates against RSV type B was similar to that against RSV type A, which indicates that the antiviral activity of these compounds lacks type specificity. At the same time, 7-aminocoumrine derivatives demonstrated some activity against RSV type A, but not type B. Among compounds with similar SI against both RSV types, **19c** was chosen to investigate a possible action mechanism.

**Figure 2:**
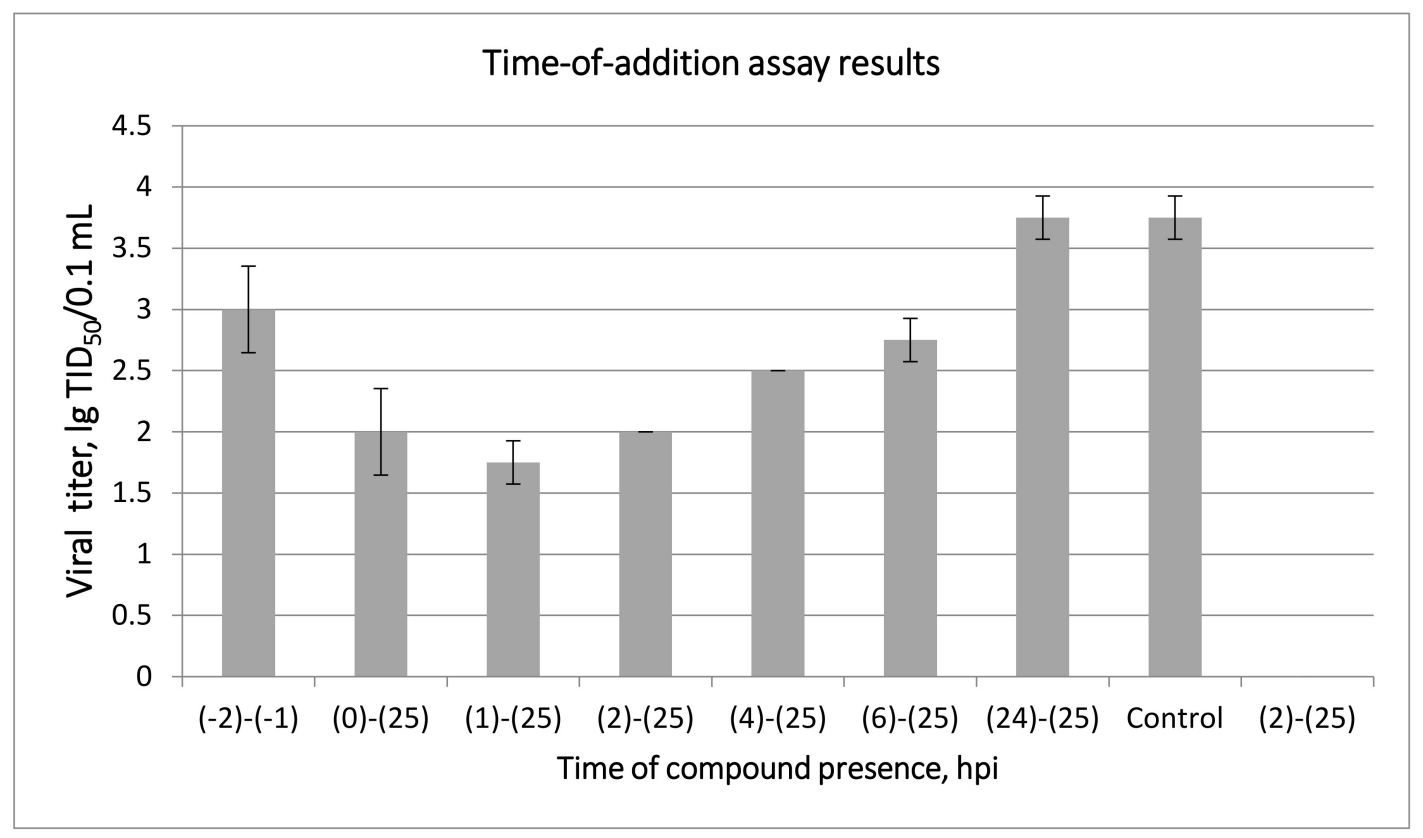
Activity of **19c** against RSV A according to time-of-addition experiment.

To elucidate action mechanisms of the most active compounds, we performed an experiment using the time-of-addition method. Compound **19c** was added to the cells with replicating virus at different time points corresponding to different stages of viral life cycle. The results are shown in Figure 2.

According to the data presented in Figure 2, compound **19c** reduces the virus titer at all time points, except for prophylactic use 2 h before virus entry into cells and late introduction after 24 h. This means that action of the compound occurred within 0–6 h after infection. A slight decrease in the virus titer was also noted at time points 2 and 4. Our data suggest that the target of **19c** may be the surface F and/or G proteins, viroporin SH, and, probably, L protein (its fragment responsible for transcription).

### 2.3. Molecular Modeling Study

According to the results of the time-of-addition experiment, the surface F protein may be considered a potential biological target. In addition, the pharmacophore features of the inhibitor of F-protein sisunatovir and compounds **19c**, **19f**, and **19h** are similar. In all cases, there are hydrophobic parts capable of hydrophobic intermolecular contacts, and donors and acceptors of hydrogen bonds (more details are presented in the Supplementary Materials).

**Figure 3:**
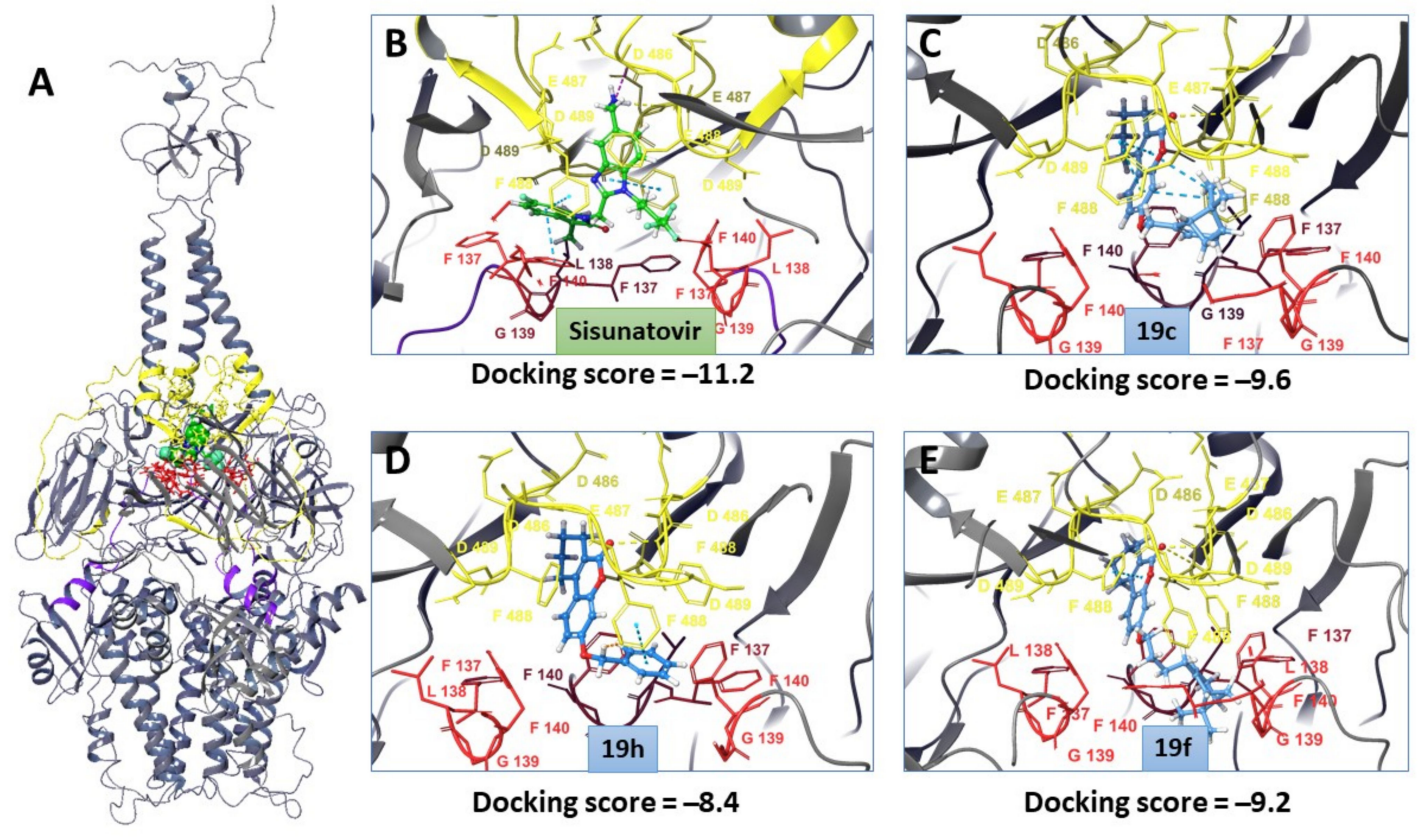
Structure of the RSV F-protein: (**A**) structure corresponds to PDB [40] code 7LVW [39]: fusion peptide region is presented in violet secondary structure (137–157 amino acids); functional residues (137–140) are shown in red; amino acids of the membrane anchor are shown in yellow (amino acids 454–499); the green molecule is sisunatovir [15]; (**B**) sisunatovir in the binding site; (**C**–**E**) positions of possible entry inhibitors (**19c**, **19f**, and, **19h**). π–π stacking interactions are denoted by blue dotted lines; H-bonds and salt bridges are denoted by yellow and violet lines, respectively.

We suggest that compounds **19c**, **19f**, and **19h** can bind to a fusion peptide region [38,39] (Figure 3A), similarly to sisunatovir [15] (Figure 3B). Several functional amino acids are located in the binding site of potential entry inhibitors (Figure 3A). The red segment in Figure 3 is a part of the fusion peptide (137–140 amino acids), and the yellow segment corresponds to amino acids of the membrane anchor. Entry inhibitors can interact with these amino acids and inhibit the conformational transition from a pre- to post-fusion conformation. The coumarin moiety of compounds **19c**, **19f**, and **19h** occurs in a cavity surrounded by phenylalanines and forms π–π stacking interactions with them (Figure 3C,D). The terpene fragment of **19c** and **19f** occurs in a hydrophobic cavity composed of amino acids of the fusion peptide region (Figure 3C,E). The aromatic part of **19h** cannot be placed into the cavity due to a short linker between it and the coumarin fragments (Figure 3D). Compound **19h** forms intramolecular interactions with amino acids of the membrane anchor.

Docking scores shown in Figure 3 may be considered as a parameter that characterizes the affinity of compounds to the binding site. The best positions are shown in Figure 3. The best ligand position was chosen based on the clustering energy and formation energy of the ligand–protein complex. For more energy parameters, see the Supplementary Materials. Affinity values of lead compounds have a slightly higher value than those for sisunatovir. Compound **19h** had the lowest affinity. These results correlate with experimental data (Table S1). Thus, RSV F protein may be considered as a possible target.

## 3. Materials and Methods

### 3.1. Chemistry

#### 3.1.1. General Chemical Methods

Reagents and solvents were purchased from commercial suppliers (Sigma-Aldrich, Acros, Japan) and used as received. GC-MS: *Agilent 7890A* gas chromatograph equipped with an Agilent 5975C quadrupole mass spectrometer as a detector; quartz column HP-5MS (copolymer 5%–diphenyl–95%–dimethylsiloxane) of length 30 m, internal diameter 0.25 mm and stationary phase film thickness 0.25 µm. Optical rotation: polAAr 3005 spectrometer. ^1^H and ^13^C NMR: Bruker DRX-500 apparatus at 500.13 MHz (^1^H) and 125.76 MHz (^13^C) and Bruker Avance—III 600 apparatus at 600.30 MHz (^1^H) and 150.95 MHz (^13^C), *J* in Hz; structure was determined by analyzing the ^1^H NMR spectra, including ^1^H–^1^H double resonance spectra and ^1^H–^1^H 2D homonuclear correlation (COSY, NOESY), *J*-modulated ^13^C NMR spectra (JMOD), and ^13^C–^1^H 2D heteronuclear correlation with one-bond (C–H COSY, ^1^*J*(C,H) = 160 Hz, HSQC, ^1^*J*(C,H) = 145 Hz) and long-range spin–spin coupling constants (C–H COSY, ^1^*J*(C,H) = 160 Hz, HSQC, ^1^*J*(C,H) = 145 Hz). HR-MS: DFS Thermo Scientific spectrometer in a full scan mode (15–500 *m/z*, 70 eV electron impact ionization, direct sample administration).

Spectral and analytical investigations were carried out at the Collective Chemical Service Center of the Siberian Branch of the Russian Academy of Sciences. All product yields are given for pure compounds purified by recrystallization from ethanol or isolated by column chromatography (SiO_2_; 60–200 μ; Macherey-Nagel). The purity of the target compounds was determined by GC-MS methods. All of the target compounds reported in this paper had a purity of no less than 95%.

#### 3.1.2. Synthesis of Coumarins **9**, **10**

Syntheses were carried out from resorcinol **5** and appropriate β-ketoesters **6, 7**, in accordance with [31]. Concentrated H_2_SO_4_ (5 mL, 94 mmol) was added dropwise to cooled (0–5 °C) solution of resorcinol **8** (45 mmol) and appropriate β-ketoesters (45 mmol) in dry ethanol (15 mL), with vigorous stirring. The mixture was stirred until it congealed, left overnight at r.t., and poured into ice water (150 mL). The resulting solid was filtered off and crystallized from ethanol–water (75%). The yields of **9, 10** were 64% and 70%, respectively.

#### 3.1.3. Synthesis of Bromides **11a–c,g**

(+)-Myrtenal was synthesized according to the procedure presented in [41] by the oxidation of (+)-α-pinene using a t-BuOOH/SeO_2_ system with a 57% yield. 

(+)-Myrtenol was synthesized from the corresponding aldehyde via reduction to alcohols with NaBH_4_, as described above. NaBH_4_ (10.3 mmol) was added to a cooled (0–5 °C) solution of 10.3 mmol of the appropriate aldehyde in methanol (20 mL), and the reaction mixture was stirred for 3 h at room temperature. Then, 5% aqueous HCl was added to obtain a pH of 4–5. The solvent was distilled, and the product was extracted using ether and dried with Na_2_SO_4_. The solvent was evaporated; the resulting alcohol (54% yield) was used in the synthesis without purification.

(3-Methoxyphenyl)methanol was synthesized from 3-methoxybenzaldehyde via a reaction with NaBH_4_, as described above (yield: 34%).

Bromide **11a** was synthesized from geraniol via the reaction with PBr_3_ [30].

PBr_3_ (8.9 mmol) was added to cooled (0–5 °C) solution of geraniol (26.7 mmol) in dry ether (30 mL), and the reaction mixture was stirred for 2 h at r.t. Saturated aqueous NaHCO_3_ was added, and the product was extracted with ether. The extracts were washed with brine, dried with Na_2_SO_4_, and evaporated. 

Additional bromides **11b,c,g** were synthesized as described above. Compounds **11a–c,g** (with yields of 91%, 55%, 60% and 65%, respectively) were sufficiently pure and used for the next step without purification. 

#### 3.1.4. Synthesis of Bromide **11d**

Bromide 11**d** was synthesized from (−)-nopol via reaction with NBS–PPh_3_, as described in [32].

Triphenylphosphine (2.0 equiv., 6.1 g, 23 mmol) was dissolved in dry DCM (23 mL) under argon. *N*-bromosuccinimide (NBS) (2.0 equiv., 4.2 g, 23 mmol) was added to this solution in small portions over 5 min in an ice-water bath. Subsequently, the resulting deep red mixture was stirred at room temperature for 30 min. Then, pyridine (1 mL) was added, and the color turned reddish-brown. (–)-Nopol (1.0 equiv., 2.0 mL, 12 mmol) was added to the mixture dropwise over 10 min. The reaction mixture was stirred overnight at room temperature. Later, the mixture was diluted with hexane (40 mL) and filtered through a silica gel plug. Then, the reaction flask was stirred with EtOAc–hexane (1:1, 40 mL) and filtered through the silica gel plug. Later, it was concentrated in vacuo and the crude residue was purified by chromatography on SiO_2_ (hexane) to obtain bromide **11d** (2.3 g, 70% yield).

#### 3.1.5. Synthesis of Bromide **11e**

Bromide **11e** was synthesized from alcohol **13** via a reaction with NBS–PPh_3_, as described for **11d (**the yield of **11e**-41%). Alcohol **13** was obtained by the reduction of ether **12**, synthesized from (+)-*trans*-pinocarveol [34] with LiAIH_4_.

A mixture of (+)-*trans*-pinocarveol (2.45 g, 16 mmol; obtained by the oxidation of β-pinene with t-BuOOH in the presence of SeO_2_, in accordance with [33]), triethyl orthoacetate (3.93 g, 24 mmol), and hexanoic acid (0.25 g, 2.4 mmol) was heated for 6 h at 150 °C. The mixture was then diluted with Et_2_O (150 mL) and washed successively with sat. aq. NaHCO_3_ soln. and H_2_O, dried with Na_2_SO_4_, and evaporated. Compound **12** was purified by column chromatography on SiO_2_, eluent–hexane–ethyl acetate (1.92 g, yield 54%).

A solution of **12** (2.22 g, 10 mmol) in Et_2_O (10 mL) was added dropwise to a suspension of LiAIH_4_ (2.1 g, 55 mmol) in refluxing Et_2_O (20 mL) . After 1 h at r.t., the mixture was cooled to 0 °C, and H_2_O (2 mL), 15% aq. NaOH soln. (2 mL), and then H_2_O (3 mL) were added. After 30 min, the mixture was filtered and evaporated. Compound **13** was purified by column chromatography on SiO_2_, eluent–hexane–ethyl acetate (1.40 g, yield 78%).

#### 3.1.6. Synthesis of Bromide **11f**

Aldehyde **14**, obtained according to [35], was reduced to alcohol **15** by NaBH_4_ (yield: 86%). The OH group of alcohol **15** was replaced by Br using NBS–PPh_3_ (yield 11f: 84%). Then, 100 mL Et_2_O, acrolein (8.6 g, 154 mmol) and (−)-β-pinene (14.5 g, 107 mmol) were added to a solution of anhydrous ZnBr_2_ (4.3 g, 19 mmol). The solution was stirred for 48 h at 25 °C. Then, it was poured into water (200 mL) and filtered by suction to remove zinc salts. The ether layer was separated, and the aqueous layer was extracted with 2 × 50 mL Et_2_O. The combined ether layers were dried and evaporated. Compound **14** was purified by column chromatography on SiO, eluent–hexane–CHCl_3_ (30%) (**14**: 3.76 g, yield 25%, unreacted (−)-β-pinene-3.13 g).

#### 3.1.7. Synthesis of Compounds **16a**–**g**, **17a**–**c**,**e**–**g**, **18a**–**g** and **19a**–**g**

Compounds **16a**–**d,g**, were synthesized from coumarin **6** and the corresponding bromides **11a**–**d,g** with the use of DBU and DMF, in accordance with [30].

DBU (1.0 mmol) and the corresponding bromides **11a**–**d,g** (0.75 mmol) were added to compound **1** (0.5 mmol) in dry DMF (5 mL) at r.t. under stirring. The reaction mixture was stirred at r.t. for 15 min, and then heated at 60 °C for 5 h. H_2_O (15 mL) was added, and the product was extracted with ethyl acetate. The extracts were washed with brine, dried with Na_2_SO_4_, and evaporated. 

Compounds **16e,f**, **16e,f**, **17e,f** and **18e,f** were synthesized as above. 

Compounds **16a**, **17a**, and **18a** were synthesized from coumarins **6,7,9** and geranyl bromide **11a** with the use K_2_CO_3_ and ethanol, in accordance with [31].

K_2_CO_3_ (1.0 mmol) and geranyl bromide **11a** (0.75 mmol) were added to corresponding compounds **5–7** (0.5 mmol) in dry ethanol (5 mL) at r.t. under stirring. The reaction mixture was stirred at r.t. for 15 min, and then heated at 60 °C for 5 h. The hot solution was filtered; the filtrate was kept at −18 °C for 48 h. 

Compounds **17a**–**c,g**, **18a**–**d,g**, and **19a**–**d,g** were synthesized from coumarins **7**,**9**,**10** and corresponding bromides **11a**–**d,g** with the use K_2_CO_3_ and ethanol, as described above.

^1^H NMR spectra of **16a**–**d,g**, **17a**–**c,g**, **18a**–**d,g**, and **19a**–**d,g** coincided with the corresponding spectra published in the literature [30,31].

Products **16a**–**g**, **17a**–**c,e**–**g**, **18a**–**g**, and **19a**–**g** were isolated in the individual form: (**a**) by recrystallization from ethanol; or (**b**) by column chromatography on a silica gel, eluent–hexane, solution containing from 25% to 100% chloroform in hexane, ethanol.

*7-(3-((1R,5S)-6,6-Dimethylbicyclo[3.1.1]hept-2-en-2-yl)propoxy)-2H-chromen-2-one* (**16e**). Yield: 58%, method **b**. M.p. 75 °C. ${\left[\alpha \right]}_{589}^{24.5}$ = −19.4 (*c* = 1.40, CHCl_3_). HRMS: 324.1714 ([M]^+^, *m*/*z* calcd for C_21_H_24_O_3_ 324.1720). ^1^H-NMR (CDCl_3_, δ_H_): 0.80 (s, 3H-C(21)); 1.11 (d, 1H, ^2^J = 8.5, H_anti_ H–C(19)); 1.24 (s, 3H-C(20)); 1.77–1.89 (m, 2H, 2H-C(11)); 2.00 (ddd, 1H, J_18,16_ = J_18,19sin_ = 5.6, J_18,14_ = 1.4, H-C(18)); 2.03–2.11 (m, 3H, 2H-C(12)), 1H-C(16)); 2.15 (dm, 1H, ^2^J = 17.5, H-C(15)); 2.22 (dm, 1H, ^2^J = 17.5, H’-C(15)); 2.34 (ddd, 1H, ^2^J = 8.5, J_19sin,16_ = J_19sin,18_ = 5.6, H_sin_-C(19)); 3.94–3.98 (m, 2H, 2H-C(10)); 5.18–5.22 (m, 1H, H-C(14)); 6.19 (d, 1H, J_3,4_ = 9.5, H-C(3)); 6.75 (d, 1H, J_9,7_ = 2.4, H-C(9)); 6.79 (dd, 1H, J_7,6_ = 8.5, J_7.9_ = 2.4, H-(C-7)); 7.32 (d, 1H, J_6.7_ = 8.5, H-6); 7.59 (d, 1H, J_4,3_ = 9.5, H-4). ^13^C-NMR (CDCl_3_, δ_C_): 155.74 (s, C(1)); 161.05 (s, C(2)); 112.72 (d, C(3)); 143.27 (d, C(4)); 112.21 (s, C(5)); 128.53 (d, C(6)); 112.78 (d, C(7)); 162.24 (s, C(8)); 101.15 (d, C(9)); 68.11 (t, C(10)); 26.42 (t, C(11)); 32.83 (t, C(12)); 146.90 (s, C(13)); 116.61 (d, C(14)); 31.09 (t, C-(15)); 40.66 (d, C(16)); 37.78 (s, C(17)); 45.58 (d, C(18)); 31.53 (t, C(19)); 26.14 (q, C(20)); 21.02 (q, C(21)).

*7-(4-((1R,5S)-6,6-Dimethylbicyclo[3.1.1]hept-2-en-2-yl)butoxy)-2H-chromen-2-one* (**16f**). Yield: 80%, method **b**. ${\left[\alpha \right]}_{589}^{22}$ = −9.0 (*c* = 1.78, CHCl_3_). HRMS: 337.1793 ([M-H]^+^, *m*/*z* calcd for C_22_H_25_O_3_ 337.1798). ^1^H-NMR (CDCl_3_, δ_H_): 0.80 (s, 3H-C(22)); 1.11 (d, 1H, ^2^J = 8.5, H_anti_-C(20)); 1.24 (s, 3H-C(21)); 1.42–1.56 (m, 2H, 2H-C(12)); 1.74–1.81 (m, 2H, 2H-C(11)); 1.95–2.02 (m, 3H, 2H-C(13)), 1H-C(19)); 2.02–2.08 (m, 1H, H-C(17)); 2.15 (dm, 1H, ^2^J = 17.4, H-C(16)); 2.22 (dm, 1H, ^2^J = 17.4, H’-C(16)); 2.32 (ddd, 1H, ^2^J = 8.5, J_20sin,17_ = J_20sin,19_ = 5.6, H_sin_-C(20)); 3.98 (t, 2H, J_10,11_ = 6.5, 2H-C(10)); 5.16–5.20 (m, 1H, H-C(15)); 6.20 (d, 1H, J_3,4_ = 9.5, H-C(3)); 6.76 (d, 1H, J_9,7_ = 2.4, H-C(9)); 6.79 (dd, 1H, J_7,6_ = 8.6, J_7.9_ = 2.4, H-C(7)); 7.32 (d, 1H, J_6.7_ = 8.6, H-C(6)); 7.59 (d, 1H, J_4,3_ = 9.5, H-C(4)). ^13^C-NMR (CDCl_3_, δ_C_): 155.77 (s, C(1)); 161.07 (s, C(2)); 112.74 (d, C(3)); 143.26 (d, C(4)); 112.22 (s, C(5)); 128.53 (d, C(6)); 112.79 (d, C(7)); 162.26 (s, C(8)); 101.21 (d, C(9)); 68.38 (t, C(10)); 28.56 (t, C(11)); 23.32 (t, C(12)); 36.31 (t, C(13)); 147.68 (s, C(14)); 116.14 (d, C(15)); 31.12 (t, C(16)); 40.73 (d, C(17)); 37.78 (s, C(18)); 45.60 (d, C(19)); 31.52 (t, C(20)); 26.19 (q, C(21)); 21.05 (q, C(22)).

7*-(3-((1R,5S)-6,6-Dimethylbicyclo[3.1.1]hept-2-en-2-yl)propoxy)-4-methyl-2H-chromen-2-one* (**17e**). Yield: 49%, method **a**. M.p. 86 °C. ${\left[\alpha \right]}_{589}^{24.5}$ = −19.5 (*c* = 0.65, CHCl_3_). HRMS: 337.1796 ([M-H]^+^, m/z calcd for C_22_H_25_O_3_ 337.1798). ^1^H-NMR (CDCl_3_, δ_H_): 0.81 (s, 3H-C(22)); 1.13 (d, 1H, ^2^J = 8.5, H_anti_-C(20)); 1.25 (s, 3H-C(21)); 1.78–1.90 (m, 2H, 2H-C(12)); 2.01 (ddd, 1H, J_19,17_ = J_19,20sin_ = 5.6, J_19,15_ = 1.3, H-C(19)); 2.04–2.12 (m, 3H, 2H-C(13)), 1H-C(17)); 2.17 (dm, 1H, ^2^J = 17.5, H-C(16)); 2.23 (dm, 1H, ^2^J = 17.5, H’-C(16)); 2.35 (ddd, 1H, ^2^J = 8.5, J_20sin,17_ = J_20sin,19_ = 5.6, H_sin_-C(20)); 2.37 (d, 3H, J_10,3_ = 0.8, 3H-C(10)); 3.97 (t, 2H, J_11,12_ = 6.5, 2H-C(11)); 5.20–5.23 (m, 1H, H-C(15)), 6.09 (q, 1H, J_3,10_ = 0.8, H-C(3)); 6.77 (d, 1H, J_9,7_ = 2.5, H-C(9)); 6.82 (dd, 1H, J_7,6_ = 8.8, J_7,9_ = 2.5, H-C(7)); 7.46 (d, 1H, J_6,7_ = 8.8, H-C(6)). ^13^C-NMR (CDCl_3_, δ_C_): 155.16 (s, C(1)); 161.24 (s, C(2)); 111.66 (d, C(3)); 152.46 (s, C(4)); 113.28 (s, C(5)); 125.32 (d, C(6)); 112.55 (d, C(7)); 162.09 (s, C(8)); 101.19 (d, C(9)); 18.53 (q, C(10)); 68.10 (t, C(11)); 26.47 (t, C(12); 32.88 (t, C(13)); 146.96 (s, C(14)); 116.64 (d, C(15)); 31.13 (t, C(16)); 40.69 (d, C(17)); 37.83 (s, C(18)); 45.60 (d, C(19)); 31.57 (t, C(20)); 26.18 (q, C(21)); 21.06 (q, C(22)).

*7-(4-((1R,5S)-6,6-Dimethylbicyclo[3.1.1]hept-2-en-2-yl)butoxy)-4-methyl-2H-chromen-2-one* (**17f**). Yield: 24%, method **a**. M.p. 77 °C. ${\left[\alpha \right]}_{589}^{26}$ = −20.3 (*c* = 0.60, CHCl_3_). HRMS: 351.1954 ([M-H]^+^, *m/z* calcd for C_23_H_27_O_3_ 351.1955). ^1^H-NMR (CDCl_3_, δ_H_): 0.81 (s, 3H-C(23)); 1.11 (d, 1H, ^2^J = 8.5, H_anti_-C(21)); 1,24 (s, 3H-C(22)); 1.43–1.57 (m, 2H, 2H-C(13)); 1.74–1.82 (m, 2H, 2H-C(12)); 1.96–2.02 (m, 3H, 2H-C(14)), 1H-C(20)); 2.03–2.08 (m, 1H, H-C(18)); 2.16 (dm, 1H, ^2^J = 17.5, H-C(17)); 2.23 (dm, 1H, ^2^J = 17.5, H’-C(17)); 2.33 (ddd, 1H, ^2^J = 8.5, J_21sin,18_ = J_21sin,20_ = 5.6, H_sin_-C(21)); 2.37 (d, 3H, J_10,3_ = 1.2, 3H-C(10)); 3.98 (t, 2H, J_11,12_ = 6.5, 2H-C(11)); 5.16–5.20 (m, 1H, H-C(16)); 6.10 (q, 1H, J_3,10_ = 1.2, H-C(3)); 6.77 (d, 1H, J_9,7_ = 2.5, H-C(9)); 6.82 (dd, 1H, J_7,6_ = 8.8, J_7,9_ = 2.5, H-C(7)); 7.45 (d, 1H, J_6,7_ = 8.8, H-C(6)). ^13^C-NMR (CDCl_3_, δ_C_): 155.14 (s, C(1)); 161.25 (s, C(2)); 111.65 (d, C(3)); 152.46 (s, C(4)); 113.24 (s, C(5)); 125.31 (d, C(6)); 112.53 (d, C(7)); 162.06 (s, C(8)); 101.18 (d, C(9)); 18.55 (q, C(10)); 68.33 (t, C(11)); 28.59 (t, C(12)); 23.33 (t, C(13); 36.35 (t, C(14)); 147.70 (s, C(15)); 116.13 (d, C(16)); 31.12 (t, C(17)); 40.70 (d, C(18)); 37.80 (s, C(19)); 45.55 (d, C(20)); 31.54 (t, C(21)); 26.20 (q, C(22)); 21.07 (q, C(23)).

*7-(3-((1R,5S)-6,6-Dimethylbicyclo[3.1.1]hept-2-en-2-yl)propoxy)-2,3-dihydrocycl penta[c]chromen-4(1H)-one* (**18e**) Yield 47%, method **a**. M.p. 68 °C. ${\left[\alpha \right]}_{589}^{24.5}$ = −18.6 (*c* = 0.88, CHCl_3_). HRMS: 364.2038 ([M]^+^, *m*/*z* calcd for C_24_H_28_O_3_ 364.2033). ^1^H-NMR (CDCl_3_, δ_H_): 0.81 (s, 3H-C(24)); 1.12 (d, 1H, ^2^J = 8.5, H_anti_-C(22)); 1.25 (s, 3H-C(23)); 1.77–1.90 (m, 2H, 2H-C(14)); 2.01 (ddd, 1 H, J_21.19_ = J_21,22sin_5.6, J_21,17_ = 1.4, H-C(21)); 2.04–2.12 (m, 3H, 2H-C(15), 1H-C(19)); 2.13–2.19 (m, 3H, 2H-C(11), 1H-C(18)); 2.23 (dm, 1H, ^2^J = 17.6, H’-C(18)); 2.34 (ddd, 1H, ^2^J = 8.5, J_22s,19_ = J_22s,21_ = 5.6, H_sin_-C(22)); 2.83–2.88 (m, 2H, 2H-C(10)); 2.98–3.03 (m, 2H, 2H-C(12)); 3.96 (t, 2H, J_13,14_ = 6.5, 2H-C(13)); 5.19–5.23 (m, 1H, H-C(17)); 6.78–6.82 (m, 2H,H-C(7), H-C(9)); 7.27–7.32 (m, 1H, H-C(6)). ^13^C-NMR (CDCl_3_, δ_C_): 155.64 (s, C(1)); 160.46 (s, C(2)); 124.16 (s, C(3)); 156.28 (s, C(4)); 112.11 (s, C(5)); 125.38 (d, C(6)); 112.45 (d, C(7)); 161.43 (s, C(8)); 101.10 (d, C(9)); 30.20 (t, C(10)); 22.43 (t, C(11)); 31.90 (t, C(12)); 68.04 (t, C(13)); 26.50 (t, C(14)); 32.90 (t, C(15)); 146.99 (s, C(16)); 116.59 (d, C(17)); 31.12 (t, C(18)); 40.68 (d, C(19)); 37.81 (s, C(20)); 45.59 (d, C(21)); 31.56 (t, C(22)); 26.17 (q, C(23)); 21.05 (q, C(24)).

*7-(4-((1R,5S)-6,6-Dimethylbicyclo[3.1.1]hept-2-en-2-yl)butoxy)-2,3-dihydrocyclopenta[c]chromen-4(1H)-one* (**18f**). Yield: 53%, method **a**. M.p. 61 °C. ${\left[\alpha \right]}_{589}^{26}$ = −17.0 (*c* = 1.00, CHCl_3_). HRMS: 377.2110 ([M-H]^+^, *m*/*z* calcd for C_25_H_29_O_3_ 377.2111). ^1^H-NMR (CDCl_3_, δ_H_): 0.80 (s, 3H-C(25)); 1.11 (d, 1H, ^2^J = 8.5, H_anti_-C(23)); 1.24 (s, 3H-C(24)); 1.43–1.56 (m, 2H, 2H-C(15)); 1.73–1.81 (m, 2H, 2H-C(14)); 1.95–2.01 (m, 3H, 2H-C(16), H-C(22)); 2.02–2.07 (m, 1H, H-C(20); 2.12–2.19 (m, 3H, 2H-C(11), 1H-C(19)); 2.22 (dm, 1H, ^2^J = 17.5, H’-C(19)); 2.32 (ddd, 1H, ^2^J = 8.5, J_23sin,20_ = J_23sin,22_ = 5.6, H_sin_-C(23)); 2.82–2.88 (m, 2H, 2H-C(10)); 2.97–3.03 (m, 2H, 2H-C(12)); 3.97 (t, 2H, J_13,14_ = 6.5, 2H-C(13)); 5.16–5.20 (m, 1H, H-C(18)); 6.78–6.82 (m, 2H, H-C(7), H-C(9)); 7.26–7.32 (m, 1H, H-C(6)). ^13^C-NMR (CDCl_3_, δ_C_): 155.62 (s, C(1)); 160.48 (s, C(2)); 124.14 (s, C(3)); 156.29 (s, C(4)); 112.07 (s, C(5)); 125.37 (d, C(6)); 112.42 (d, C(7)); 161.40 (s, C(8)); 101.10 (d, C(9)); 30.19 (t, C(10)); 22.41 (t, C(11)); 31.90 (t, C(12)); 68.26 (t, C(13)); 28.62 (t, C(14)); 23.34(t, C(15)); 36.35 (t, C(16)); 147.72 (s, C(17)); 116.10 (d, C(18)); 31.11 (t, C(19)); 40.69 (d, C(20)); 37.79 (s, C(21)); 45.54 (d, C(22)); 31.53 (t, C(23)); 26.19 (q, C(24)); 21.06 (q, C(25)).

*3-(3-((1R,5S)-6,6-Dimethylbicyclo[3.1.1]hept-2-en-2-yl)propoxy)-7,8,9,10-tetrahydro-6H-benzo[c]chromen-6-one* (**19e**). Yield: 33%, method **a**. M.p. 70 °C. ${\left[\alpha \right]}_{589}^{24.5}$ = −17.8 (*c* = 0.73, CHCl_3_). HRMS: 377.2110 ([M-H]^+^, *m*/*z* calcd for C_25_H_29_O_3_ 377.2111). ^1^H-NMR (CDCl_3_, δ_H_): 0.81 (s, 3H-C(25)); 1.12 (d, 1H, ^2^J = 8.6, H_anti_-C(23)); 1.25 (s, 3H-C(24)); 1.73–1.89 (m, 6H, 2H-C(11), 2H-C(12), 2H-C(15)); 2.01 (ddd, 1 H, J_22.20_ = J_22,23sin =_ 5.6, J_22,18_ = 1.4, H-C(22)); 2.04–2.12 (m, 3H, 2H-C(16), 1H-C(20)); 2.16 (dm, 1H, ^2^J = 17.5, H-C(19)); 2.23 (dm, 1H, ^2^J = 17.5, H’-C(19)); 2.34 (ddd, 1H, ^2^J = 8.6, J_23sin,20_ = J_23sin,22_ = 5.6, H_sin_-C(23)); 2.50–2.55 (m, 2H, 2H-C(10)); 2.69–2.74 (m, 2H, 2H-C(13)); 3.96 (t, 2H, J_14,15_ = 6.5, 2H-C(14)); 5.19–5.23 (m, 1H, H-C(18)); 6.75 (d, 1H, J_9,7_ = 2.5, H-C(9)); 6.80 (dd, 1H, J_7,6_= 8.8, J_7,9_ = 2.5, H-C(7)); 7.41 (d, 1H, J_6,7_ = 8.8, H-C(6)). ^13^C-NMR (CDCl_3_, δ_C_): 153.38 (s, C(1)); 162.09 (s, C(2)); 120.22 (s, C(3)); 147.19 (s, C(4)); 113.46 (s, C(5)); 123.90 (d, C(6)); 112.23 (d, C(7)); 160.85 (s, C(8)); 100.97 (d, C(9)); 23.69 (t, C(10)); 21.57 (t, C(11)); 21.27 (t, C(12)); 25.08 (t, C(13)); 67.99 (t, C(14)); 26.52 (t, C(15)); 32.91 (t, C(16)); 147.03 (s, C(17)); 116.57 (d, C(18)); 31.13 (t, C(19)); 40.70(d, C(20)); 37.82 (s, C(21)); 45.61 (d, C(22)); 31.57 (t, C(23)); 26.18 (q, C(24)), 21.06 (q, C(25)).

*3-(4-((1R,5S)-6,6-Dimethylbicyclo[3.1.1]hept-2-en-2-yl)butoxy)-7,8,9,10-tetrahydro-6H-benzo[c]chromen-6-one* (**19f**). Yield: 36%, method **a**. M.p. 69 °C. ${\left[\alpha \right]}_{589}^{27}$ = −17.6 (*c* = 0.90, CHCl_3_). HRMS: 391.2265 ([M-H]^+^, *m*/*z* calcd for C_26_H_31_O_3_ 391.2268). ^1^H-NMR (CDCl_3_, δ_H_): ^1^H-NMR (CDCl_3_, δ_H_): 0.81 (s, 3H-C(26)); 1.11 (d, 1H, ^2^J = 8.5, H_anti_-C(24)); 1.24 (s, 3H-C(25)); 1.42–1.56 (m, 2H, 2H-C(16)); 1.73–1.86 (m, 6H, 2H-C(11), 2H-C(12), 2H-C(15)); 1.95–2.02 (m, 3H, 2H-C(17), H-C(23)); 2.02–2.08 (m, 1H, H-C(21)); 2.16 (dm, 1H, ^2^J = 17.4, H-C(20)); 2.22 (dm, 1H, ^2^J = 17.4, H’-C(20)); 2.33 (ddd, 1H, ^2^J = 8.5, J_24sin,21_ = J_24sin,23_ = 5.6, H_sin_-C(24)); 2.49–2.55 (m, 2H, 2H-C(10));)); 2.68–2.75 (m, 2H, 2H-C(13)); 3.96 (t, 2H, J_14,15_ = 6.5, 2H-C(14)); 5.16–5.20 (m, 1H, H-C(19)); 6.75 (d, 1H, J_9,7_ = 2.5, H-C(9)); 6.79 (dd, 1H, J_7,6_= 8.8, J_7,9_ = 2.5, H-C(7)); 7.41 (d, 1H, J_6,7_ = 8.8, H-C(6)). ^13^C-NMR (CDCl_3_, δ_C_): 153.36 (s, C(1)); 162.10 (s, C(2)); 120.19 (s, C(3)); 147.19 (s, C(4)); 113.42 (s, C(5)); 123.89 (d, C(6)); 112.19 (d, C(7)); 160.82 (s, C(8)); 100.95 (d, C(9)); 23.68 (t, C(10)); 21.56 (t, C(11)); 21.25 (t, C(12)); 25.08 (t, C(13)); 68.20 (t, C(14)); 28.64 (t, C(15)); 23.35 (t, C(16)); 36.37(t, C(17)); 147.74 (s, C(18)); 116.09 (d, C(19)); 31.12 (t, C(20)); 40.70 (d, C(21)); 37.80 (s, C(22)); 45.55 (d, C(23)); 31.54 (t, C(24)); 26.20 (q, C(25)), 21.07 (q, C(26)).

#### 3.1.8. Synthesis of 7-aminocoumarines **23** and **24**

7-Aminocoumarins **23** and **24** were synthesized from m-aminophenol **20**, in accordance with [37].

Methoxycarbonyl chloride (3.6 mL 47 mmol) was added dropwise to a cooled (5–10 °C) suspension of m-aminophenol **20** (4.4 g, 40 mmol) and K_2_CO_3_ (3.5 g) in 35 mL of ethyl acetate and 3 mL of water, with vigorous stirring. The mixture was stirred for 1 h; then, 10 mL of water was added, and the mixture was stirred for another 3 h. The product was extracted with ethyl acetate. The extracts were washed with water, 1 M H_2_SO_4_, and brine, dried with Na_2_SO_4_, and evaporated. The resulting solid was crystallized from benzene to give 5.7 g of **21** (77%).

A mixture of compound **21** (4.6 g, 28 mmol) and 5.5 mL acetoacetic ester was added dropwise to 12 mL H_2_SO_4_ with vigorous stirring. The mixture was stirred for 2 h and diluted with 50 mL of ice-water. The precipitate was removed by filtration, washed with water, MeOH, and ether, and dried to give 4,0 g of **22** (61%).

A suspension of 2.8 g (12 mmol) of compound **22** in 6 mL of 45% KOH solution was stirred at 90 °C for 0.5 h until the solution formed. The mixture was cooled and diluted with water and acidified with concentrated HCl to pH 5–6. A solution of alkali was added to the suspension, to obtain pH 8. The mixture was stirred until crystallization ceased. The precipitate was removed by filtration, washed with water, MeOH, ether and dried to give 1.53 g of **23** (73%).

Similarly, compound **24** was synthesized from compound **21** with a yield of 64%.

#### 3.1.9. Synthesis of 7-aminocoumarines **25**–**27**

Amine **25** was obtained by the interaction of compound **23** and (−)-nopinal and subsequent reduction with NaBH_3_CN, in accordance with [42].

Compound **25** (0.097 g,1.0 mmol), (−)-nopinal (0.112 g 1.2 mmol) (synthesized by the oxidation of (−)-nopol with IBX according to the procedure [43]) and acetic acid (100 μL) were dissolved in methanol (5 mL) and stirred at room temperature for 2.5 h. NaBH_3_CN (0.110 g, 2.0 mmol) was added, and the reaction mixture was stirred at room temperature for 1.5 h. Methanol was evaporated and the reaction mixture was extracted with CH_2_Cl_2_. The organic layer was washed with brine, dried over anhydrous Na_2_SO_4_, filtered, and evaporated. The residue was crystallized from ethanol to give 0.115 g of **25** (64%).

Similarly, compound **27** was synthesized from amine **24** and (−)-myrtenal (yield-57%).

7-N-acetylaminocoumarin **26** was synthesized from 7-aminocoumarin **23**, in accordance with [38].

A mixture of 7-aminocoumarin **23** (0.200 g, 1.1 mmol) and DMAP (20 mg) was dissolved in 1 mL CH_2_Cl_2_. Acetic anhydride (0.2 mL, 2.1 mmol) was added, and the mixture was stirred at room temperature for 24 h. On completion of the reaction, 10 mL of ice-cold water was added. The precipitate was filtered, washed with water, and dried. The resulting solid was crystallized from ethanol to give 0.176 g of **26** (71%). NMR spectrum **26** coincided with the corresponding spectrum published in the literature [38].

*7-(2-((1R,5S)-6,6-Dimethylbicyclo[3.1.1]hept-2-en-2-yl)ethylamino)-4-methyl-2H-chromen-2-one* (**25**). Yield: 64% M.p. 131 °C. ${\left[\alpha \right]}_{589}^{27}$ = −19.7 (*c* = 0.95, CHCl_3_). HRMS: 323.1878 ([M]^+^, *m*/*z* calcd for C_21_H_25_O_2_N_1_ 323.1880). ^1^H-NMR (CDCl_3_, δ_H_): 0.81 (s, 3H-C(21)); 1.11 (d, 1H, ^2^J = 8.6, H_anti_-C(19)); 1.25 (s, 3H-C(20)); 2.04 (ddd, 1 H, J_18.16_ = J_18,19sin =_ 5.6, J_18,14_ = 1.4, H-C(18)); 2.07–2.11 (m, 1H, H-C(16)); 2.21 (dm, 1H, ^2^J = 17.6, H-C(15)); 2.25–2.33 (m, 3H, 22H-C(12), 1H’-C(15)); 2.31 (d, 3H, J_10,3_ = 1.2, 3H-C(10)); 2.36 (ddd, 1H, ^2^J = 8.6, J_19sin,16_ = J_19sin,18_ = 5.6, H_sin_-C(19)); 3.10–3.19 (m, 2H, 2H-C(11)); 5.32–5.36 (m, 1H, H-C(14)); 5.95 (q, 1H, J_3,10_ = 1.2, H-C(3)); 6.44 (d, 1H, J_9,7_ = 2.4, H-C(9)); 6.49 (dd, 1H, J_7,6_ = 8.7, J_7,9_ = 2.4, H-C(7)); 7.32 (d, 1H, J_6,7_ = 8.7, H-C(6)).

^13^C-NMR (CDCl_3_, δ_C_): 155.83 (s, C(1)); 161.80 (s, C(2)); 109.39 (d, C(3)); 152.79 (s, C(4)); 110.64 (s, C(5)); 125.34 (d, C(6)); 110.57 (d, C(7)); 150.96 (s, C(8)); 98.32 (d, C(9)); 18.40 (q, C(10)); 40.89 (t, C(11)); 35.79 (t, C(12)); 144.85 (s, C(13)); 119.21 (d, C(14)); 31.24 (t, C(15)); 40.59 (d, C(16)); 37.86 (s, C(17)); 45.13 (d, C(18)); 31.64 (t, C(19)); 26.08 (q, C(20)); 21.10 (q, C(21)).

3-(((1R,5S)-6,6-Dimethylbicyclo[3.1.1]hept-2-en-2-yl)methylamino)-7,8,9,10-tetrahydro-6H-benzo[c]chromen-6-one **(27).** Yield: 57%. ${\left[\alpha \right]}_{589}^{27}$ = −16.3 (*c* = 0.74, CHCl_3_). HRMS: 349.2040 ([M]^+^, *m*/*z* calcd for C_23_H_27_O_2_N_1_ 349.2036). ^1^H-NMR (CDCl_3_, δ_H_): 0.79 (s, 3H-C(23)); 1.12 (d, 1H, ^2^J = 8.6, H_anti_-C(21); 1.25 (s, 3H-C(22)); 1.71–1.82 (m, 4H, 2H-C(11), 2H-C(12)); 2.05–2.10 (m, 2H, H-C(18), H-C(20)); 2.19 (dm, 1H, ^2^J = 17.8, H-C(17)); 2.26 (dm, 1H, ^2^J = 17.8, H’-C(17)); 2.36 (ddd, 1H, ^2^J = 8.6, J_21sin,18_ = J_21sin,20_ = 5.6, H_sin_-C(21)); 2.47–2.52 (m, 2H, 2H-C(10)); 2.64–2.70 (m, 2H, 2H-C(13)); 3.62–3.66 (m, 2H, 2H-C(14)); 5.41–5.45 (m, 1H, H-C(16)); 6.45 (d, 1H, J_9,7_ = 2.4, H-C(9)); 6.50 (dd, 1H, J_7,6_= 8.7, J_7,9_ = 2.4, H-C(7)); 7.28 (d, 1H, J_6,7_ = 8.7, H-C(6)).^13^C-NMR (CDCl_3_, δ_C_): 153.92 (s, C(1)); 162.57 (s, C(2)); 117.92 (s, C(3)); 147.63 (s, C(4)); 110.83 (s, C(5)); 123.77 (d, C(6)); 110.51 (d, C(7)); 149.93 (s, C(8)); 98.57 (d, C(9)); 23.62 (t, C(10)); 21.73 (t, C(11)); 21.38 (t, C(12)); 24.99 (t, C(13)); 48.54 (t, C(14)); 144.05 (s, C(15)); 118.81 (d, C(16)); 31.01 (t, C(17)); 40.71 (d, C(18)); 38.00 (s, C(19)); 43.87 (d, C(20)); 31.46 (t, C(21)); 26.01 (q, C(22)), 21.00 (q, C(23)).

### 3.2. Biology

#### 3.2.1. Cytotoxicity Test

The compounds were weighed in amounts of 2 mg and dissolved in 100 μL of DMSO. Then, the resulting solution was adjusted with the medium to a concentration of 1000 μg/mL, and a series of twofold dilutions was prepared from it. One-day culture of HEp2 cells, grown in 96-well plates, cell concentration 3 × 10^5^/well of the plate, was checked visually in an inverted microscope for the integrity of the monolayer. Plates were selected for work where the cell closure was 60–80%.

Dilutions of the compounds at the appropriate concentration were added to the plate in a volume of 100 μL in each well in two replicates for each tested concentration. The plates were incubated for 24 h at 37 °C in the presence of 5% CO_2_. Cell viability was assessed using the MTT assay.

The MTT solution was prepared on a maintenance medium at a concentration of 0.5 mg/mL. Then, 0.1 mL of MTT solution was added to each well. After 1.5 h of MTT contact at 37 °C at a CO_2_ concentration of 5%, MTT was discarded with the cells of the well and 0.1 mL of ethyl alcohol 96% was poured, after which the optical density in the wells was measured at a wavelength of 535 nm. Based on the data obtained, the CC_50_ was calculated.

#### 3.2.2. Antiviral Activity

The antiviral activity against the respiratory syncytial virus (RSV A—strain A2, RSV B—strain 9320) was assessed in a series of threefold dilutions of test compounds, starting from ½CC_50_, which were added to HEp-2 cell culture at a double concentration, at 100 μL per well, followed by the addition of 100 μL of the virus in a series of 10-fold dilutions. Cells were incubated at 37 °C and 5% CO_2_ for 1 h. Then, the virus was washed out, and the compounds were again added at a single concentration and incubated at 37 °C and 5% CO_2_ for 6 days. For the enzyme-linked immunosorbent assay (ELISA), cell culture was fixed with cold 80% acetone at –20 °C for 15 min and then washed with phosphate-buffered saline containing 0.05% Tween 20. Next, a solution of primary mouse anti-RSV F protein antibodies was added to the culture and incubated at room temperature under continuous stirring for 2 h. Then, cells were again washed with buffer, secondary anti-mouse antibodies were added, and the cells were incubated under continuous stirring for 2 h. Then, the antibodies were washed off, and a substrate–chromogenic mixture with tetramethylbenzidine was added. After 5 min, the reaction was stopped with 0.1 M sulfuric acid, and the optical density of the solution was measured at a wavelength of 450 nm. Wells with absorbance values twofold or greater than the cell control were considered contaminated. The virus titer was calculated using the Reed and Muench method. All experiments were made in triplicate.

#### 3.2.3. Time-of-Addition Assay

Compound **19c** was added at different time points before, after or simultaneously with the introduction of the virus. The time of addition of the compound was counted from point 0—the time of entry of the virus into the cell. During the period (𢄤1)–0, the cells together with the virus were incubated at 40 °C. All other experiments were carried out at 37 °C. RSV virus A 2 mL was added to the cells at a time that was conventionally designated as point −1, after which the cells were kept for an hour at a temperature of 40 °C. Then, at point 0, the virus was unbound. The cells were transferred to a thermostat at 37 °C, where they were incubated for 25 h. After this period, the medium was taken from each well and a series of ten-fold dilutions were made on a fresh cell culture and incubated for 6 days. For each compound, 2 repetitions were made by different operators. The virus titer was estimated by ELISA. The compound was added at the following times relative to the addition of the virus: point −2—the compound was introduced one hour before cell infection (prophylactic regimen); point 0—at the moment of temperature change; points 1, 2, 4, 6, 24—after 1, 2, 4, 6 and 24 h after the temperature change, respectively. In the wells marked (−2) − (25), the compound was kept throughout the experiment, starting from point -2 and until the end of the experiment −25 h. No compound was added to the control wells; instead, a similar volume of medium was added.

### 3.3. Molecular Modeling

#### 3.3.1. Receptor and Ligand Preparation

Crystallographic structures of the RSV F protein (PDB codes 7LVW [39] and 7KQD [15]) were downloaded from the Protein Data Bank database [40]. The code of the full-length protein trimer was 7LVW; 7KQD is the code of a ligand–protein complex, but only one protein from three trimeric forms. Model protein structures were prepared using the Schrodinger Protein Preparation Wizard tool (Schrodinger Suite Software): hydrogen atoms were added and minimized; missing amino acid side chains were added; bond multiplicities were restored; solvent molecules were removed; and the entire structure was optimized in the OPLS3e force field [44] at a physiological pH value. For a correct calculation procedure, we used binding site alignment procedures 7LVW and 7KQD. As results, full-size proteins and ligands (sisunatovir) in complex were obtained.

The geometric parameters of ligands (coumarin derivatives) and sisunatovir (RSV F inhibitor) were also optimized, taking into account all permissible conformations.

#### 3.3.2. Molecular Docking

Molecular docking was performed using Schrodinger Suite (Release 2020-4) software. Coumarin derivatives were docked using the forced ligand positioning protocol (IFD) under the following conditions: flexible protein and ligand; grid matrix size of 15 Å; and amino acids (within a radius of 5 Å from the ligand) restrained and optimized, taking into account the influence of a ligand. Docking solutions were ranked by evaluating the following calculation parameters: docking score (based on GlideScore minus penalties); ligand efficiency (LE, which takes into account an atomic distribution of the scoring function); a model energy value (Emodel), including a GlideScore value, energy-unrelated interactions, and parameters of the energy spent in positioning of the ligand in the binding site.

## Figures and Tables

**Table 1:** Antiviral activity and cytotoxicity of compounds **16**–**18**, **25**–**27** against RSV A and B.

Compound		R	CC_50_ *^a^*, µM	RSV A		RSV B	
				IC_50_ *^b^*, µM	SI*^c^*	IC_50_, µM	SI *^c^*
**16a**	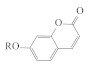	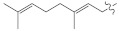	58.7 ± 7.6	6.7 ± 0.8	7.9	10.7±2.1	5.5
**16b**		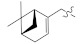	113 ± 25.6	>111	<1	34.4 ± 1.7	3.3
**16c**		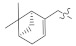	307.1 ± 34.6	27 ± 1.8	**11.4**	18.5±0.9	**16.8**
**16d**		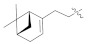	25.8 ± 1.6	2.9 ± 0.67	8.9	23.5±0.7	1.1
**16e**		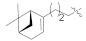	24.7 ± 6.7	>25	<1	14.8 ± 0.9	1.7
**16f**		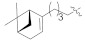	121.1 ± 24.5	2.5 ± 0.87	**64.6**	d.n.t.*^d^*	-
**16g**		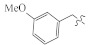	1204.4 ± 204	41.6 ± 3.2	**29.9**	37.6 ± 7.4	**32**
**17a**	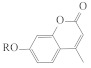	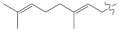	483.4 ± 23.6	46.7 ± 8.7	**10.3**	52.4±3.4	9.2
**17b**		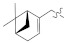	795.7 ± 28.9	29 ± 3.2	**27.4**	241 ± 41	3.3
**17c**		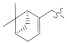	116 ± 23.6	1.3 ± 0.75	**90**	4.7±0.9	**24.7**
**17e**		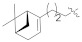	26.6 ± 12.3	12.4 ± 2.1	2.1	5 ± 1.7	5.3
**17f**		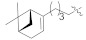	658.2 ± 54.6	13.9 ± 1.6	**62.1**	20.1±3.2	**32.7**
**17g**		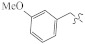	172 ± 21.1	>60.7	3	>185.6	<1.2
**18a**	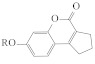	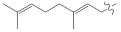	26.6 ± 12.3	>26	<1	15.1±3.4	1.8
**18b**		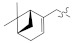	13.4 ± 4.6	>13	<1	>13	0.9
**18c**		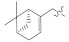	416.2 ± 32.9	4.1 ± 0.78	**100**	5.1±1.0	**81.6**
**18d**		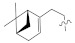	408.1 ± 56.1	>400	<1	408.6	<1
**18e**		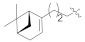	66.7 ± 23.1	4.1 ± 0.7	**18.3**	24.7 ± 5.3	2.7
**18f**		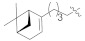	47.3 ± 12.7	50.2 ± 13.2	1	10 ± 1.7	4.7
**18g**		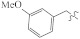	37.2 ± 9.8	>37	<1	21.7 ± 2.7	1.8
**19a**	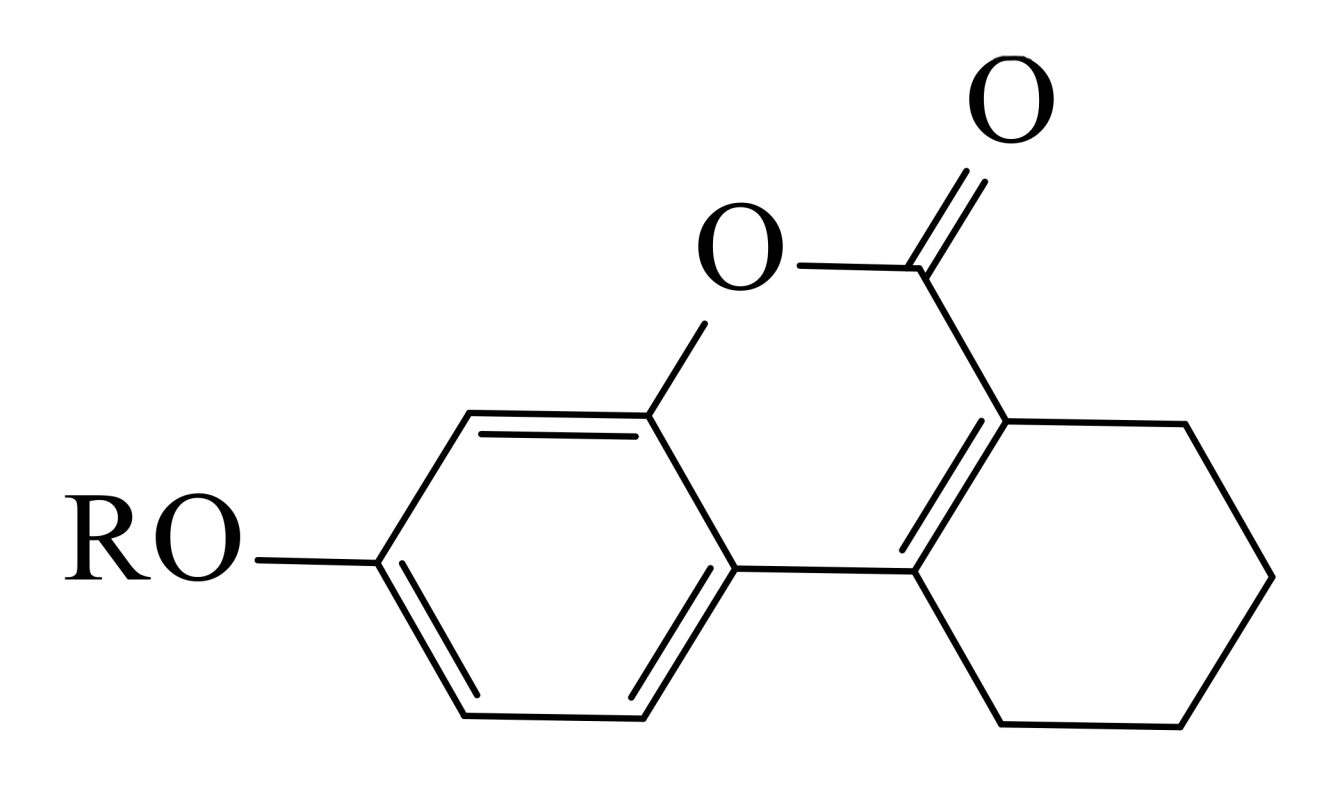	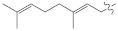	51.1 ± 11.3	0.57 ± 0.2	**90**	0.6±0.1.	**85.2**
**19b**		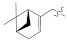	333.9 ± 56.8	107.6 ± 13.5	3.1	60.5 ± 11.6	5.5
**19c**		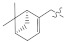	379.5 ± 29.4	5.1 ± 1.1	**77**	4.9 ± 0.6	**78.2**
**19d**		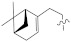	21.9 ± 11.8	0.6 ± 0.13	**40**	0.82±0.1	**26.7**
**19e**		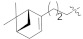	13.2 ± 4.8	6.1 ± 2.1	2.2	>13	<1
**19f**		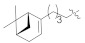	718.4 ± 21.9	15.3 ± 1.8	**57.9**	8.2 ± 0.83	**88.1**
**19g**		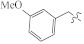	930.5 ± 56.9	81.8 ± 3.3	**11.4**	19.8±1.7	**46.9**
**19h**		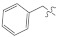	31.3 ± 7.4	>31	<1	5.2 ± 0.69	6
**25**	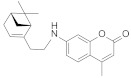		355,6 ± 12,7	33.7 ± 7.6	**10.5**	114.9 ± 16.7	3.1
**26**	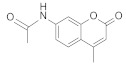		84,3 ± 11,7	6.2 ± 1.2	**14.5**	>84	<0.9
**27**	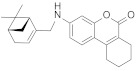		53,5 ± 10,9	51.5 ± 5.4	1	>53	<0.9
**Ribavirin**			>4095	31.1 ± 6.7	**131.6**	54.5±5.9	**75.1**

*^a^* CC_50_ is the median cytotoxic concentration, i.e., the concentration causing 50% cell death. *^b^* IC_50_ is the 50% inhibiting concentration, i.e., the concentration causing a 50% decrease in virus replication. *^c^* SI is the selectivity index, which is the CC_50_/IC_50_ ratio. CC_50_’s and IC_50_’s are presented as mean ± standard deviation. The values are calculated from three independent experiments. *^d^* d.n.t.—did not test.

## Data Availability

The data presented in this study are available on request from the corresponding author.

## Supplementary Materials

NMR ^1^H and ^13^C spectra of compounds **16**–**19**, **25**, and **27**; Energy parameters of the docking study procedure; Figure S1—best position of Sisunatovir; Figure S2—best position of **19c**; Figure S3—best position of **19f**; Figure S4—best position of **19h**; Figure S5—the pharmacophore features of known F-protein inhibitor of sisunatovir and compounds **19c**, **19f**, and **19h**; 1. Figure S6—Dose-response curve and half maximal inhibitory concentration (IC_50_) values of active compounds in Hep-2 cells against RSV A and B. Table S1—Energy parameters of docking study procedure.

## Sample Availability

Samples of the compounds presented in this study are available on request from the authors.
