# Supplementary material for: Monoterpene-Containing Substituted Coumarins as Inhibitors of Respiratory Syncytial Virus (RSV) Replication
Source: Molecules. 2021 Dec 10;26(24):7493. doi: 10.3390/molecules26247493 (PMC8708370; doi:10.3390/molecules26247493)
Supplement: Supplementary file 1 [file molecules-26-07493-s001.zip › molecules-1481635-supplementary.pdf]

SUPPLEMENTARY INFORMATION

Monoterpene-containing Substituted Coumarins as Inhibitors of Respiratory Syncytial Virus (RSV) Replication

Tatyana M. Khomenko[1], Anna A. Shtro[2], Anastasia V. Galochkina[2], Yulia V. Nikolaeva[2], Galina D. Petukhova[2], Sophia S. Borisevich[3], Dina V. Korchagina[1], Konstantin P. Volcho[1*], Nariman F. Salakhutdinov[1]

[1] N.N. Vorozhtsov Novosibirsk Institute of Organic Chemistry, 630090, Russia, Novosibirsk, acad. Lavrentjev ave. 9
[2] Smorodintsev Research Intitute of Influenza, 197376, Russia, St. Petersburg, professor Popova str, 15/17
[3] Ufa Chemistry Institute of the Ufa Federal Research Center, 71 Octyabrya pr., 450054 Ufa, Russia
***** Correspondence: volcho@nioch.nsc.ru

1. NMR ^{1}H and ^{13}C spectra of the compounds **16-19, 25 and 27**
2. Energy parameters of docking study procedure
3. Figure S1 – best position of Sisunatovir
4. Figure S2 – best position of **19c**
5. Figure S3 – best position of **19f**
6. Figure S4 – best position of **19h**
7. Figure S5 – the pharmacophore features of known F-protein inhibitor of sisunatovir and compounds **19c**, **19f**, and **19h**
8. Figure S6 – Dose-response curve and half maximal inhibitory concentration (IC_{50}) values of active compounds in Hep-2 cells against RSV A and B

Compound **16a**

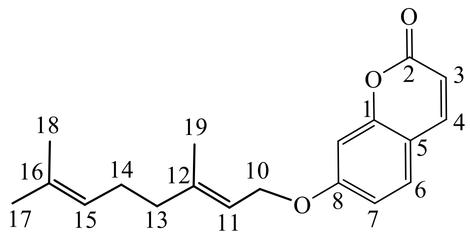

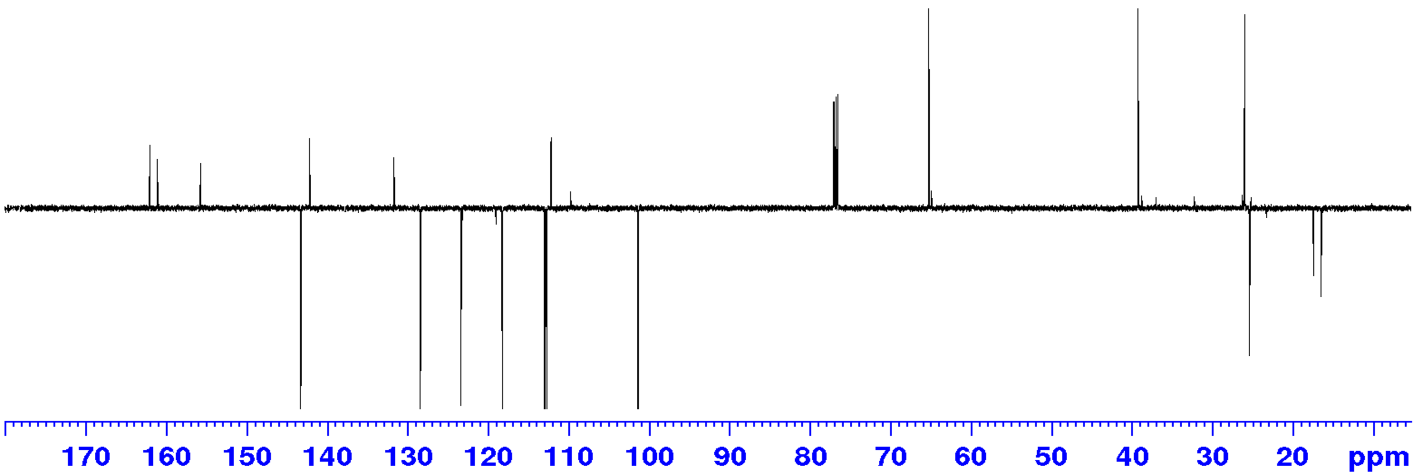

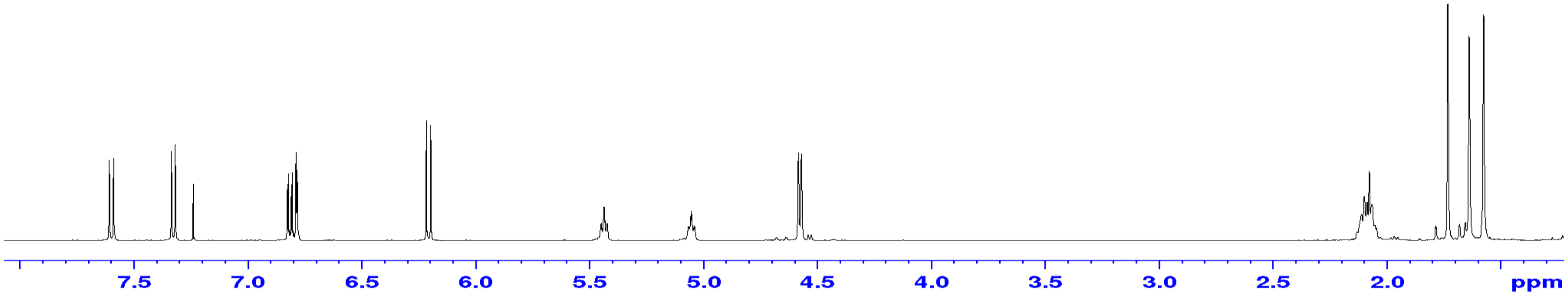

Compound **16c**

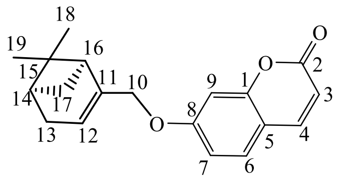

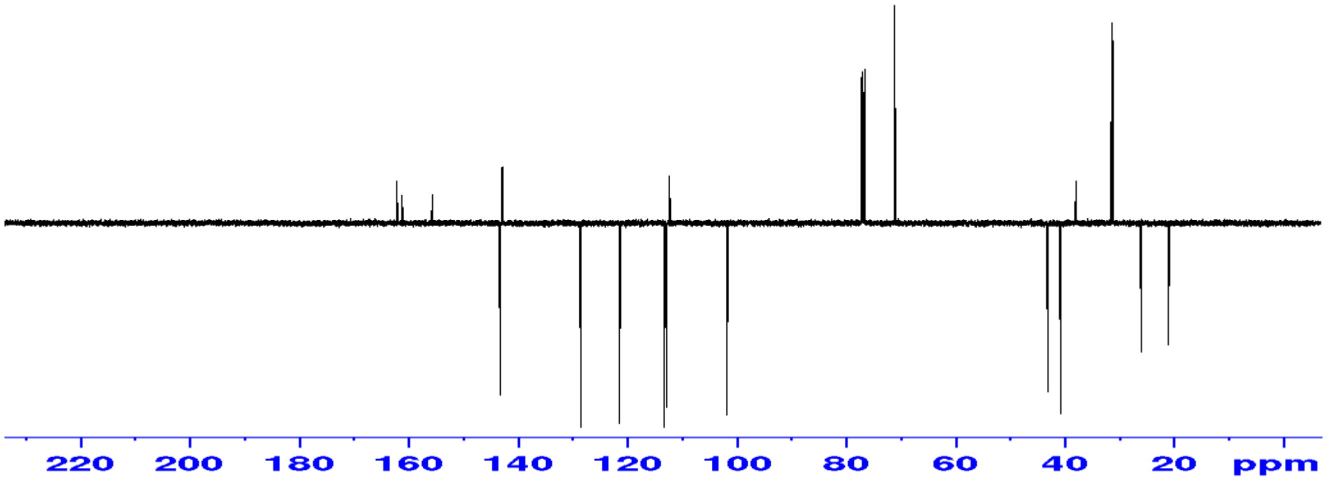

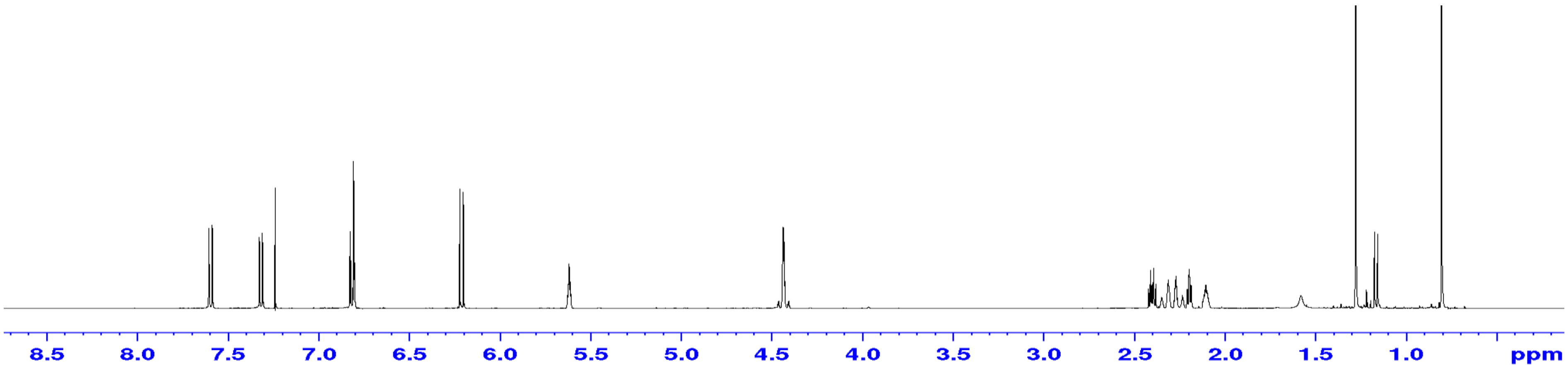

Compound **16d**

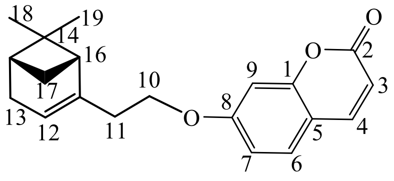

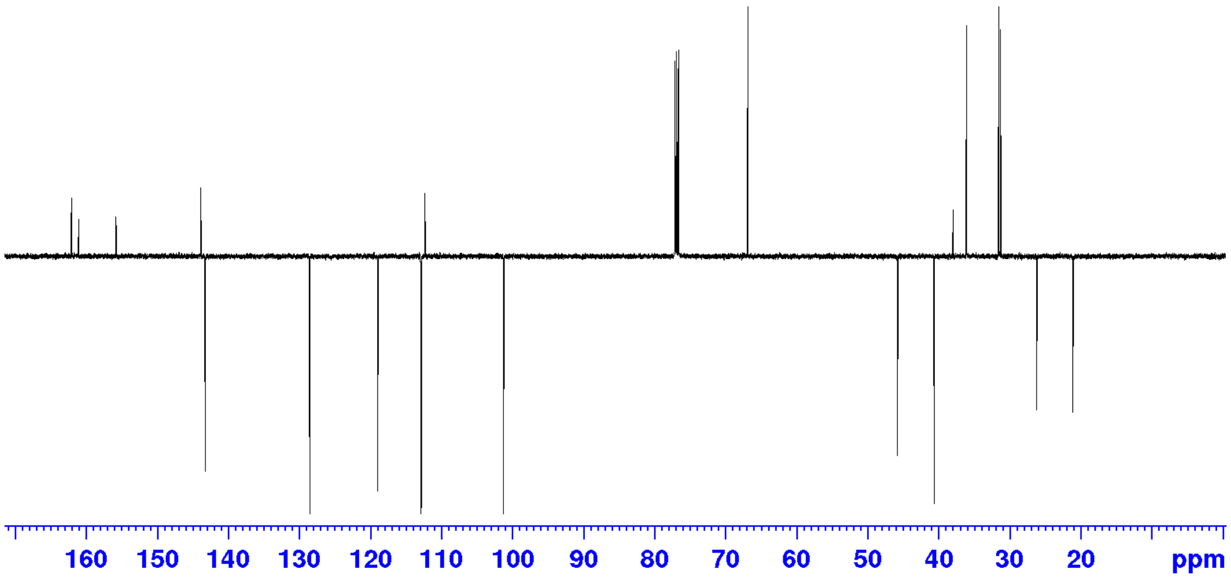

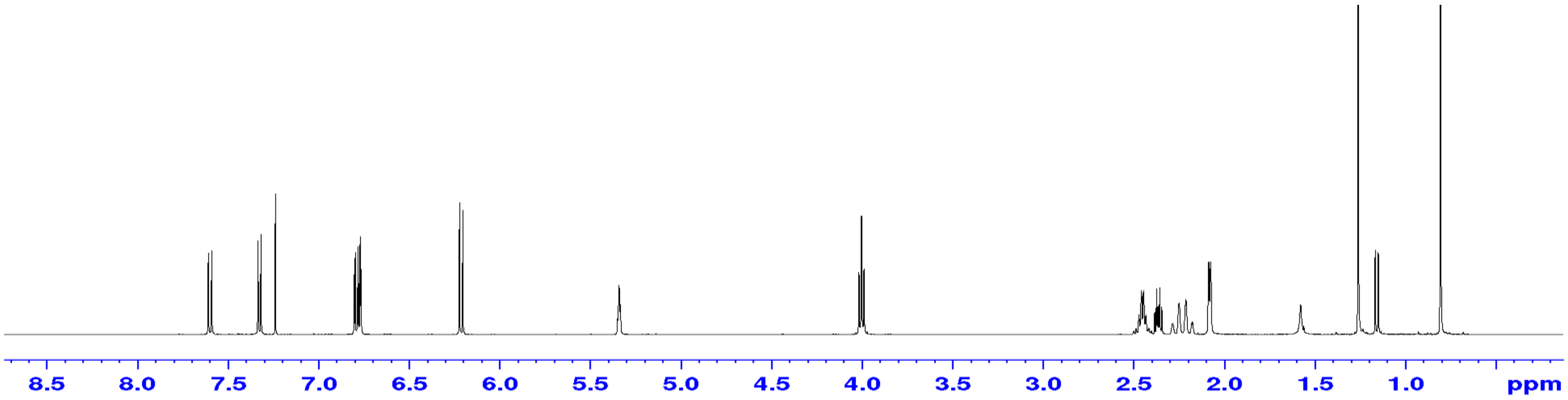

Compound **16e**

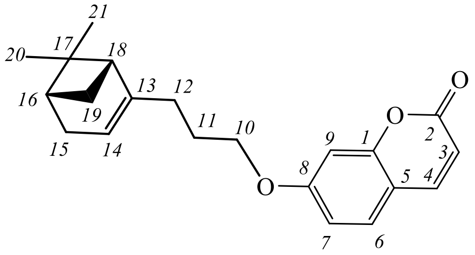

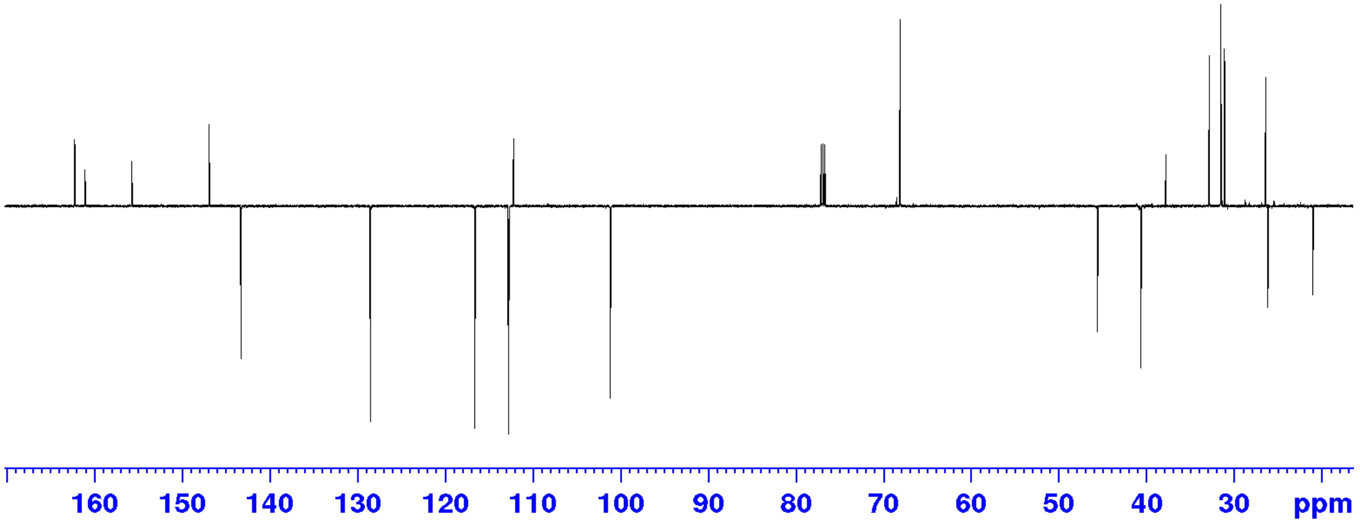

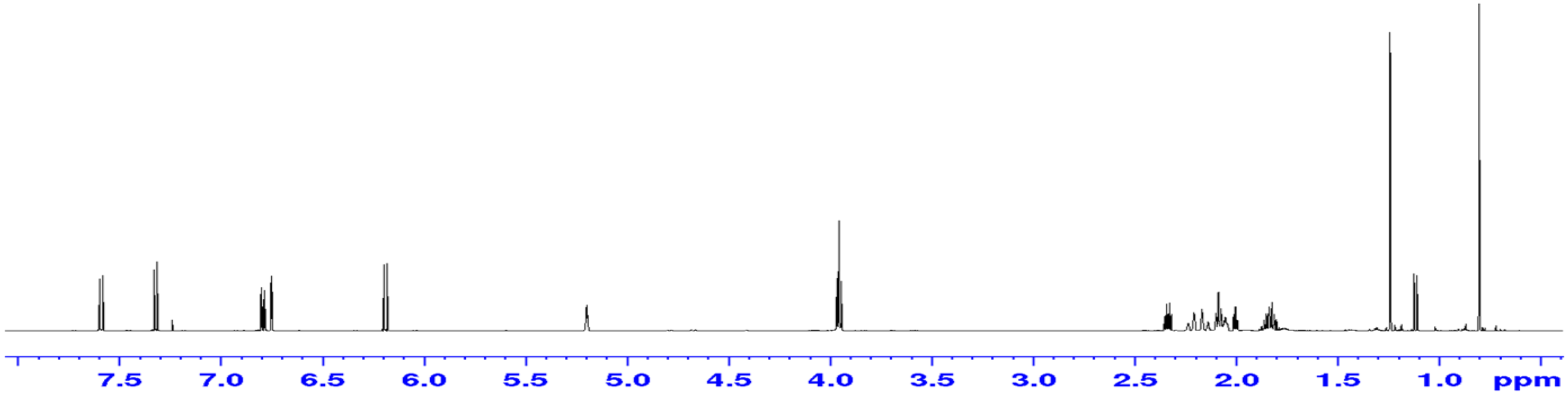

Compound **16f**

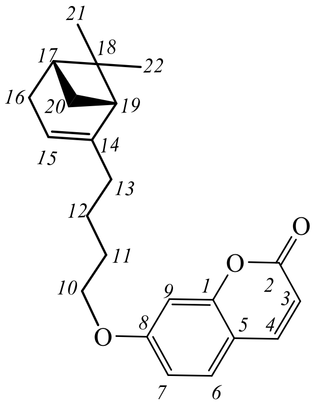

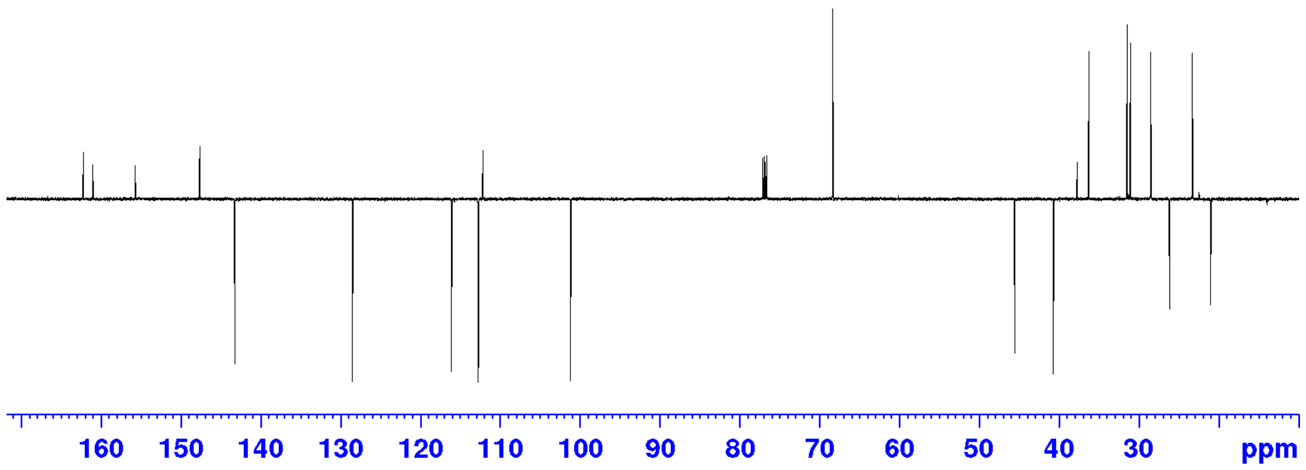

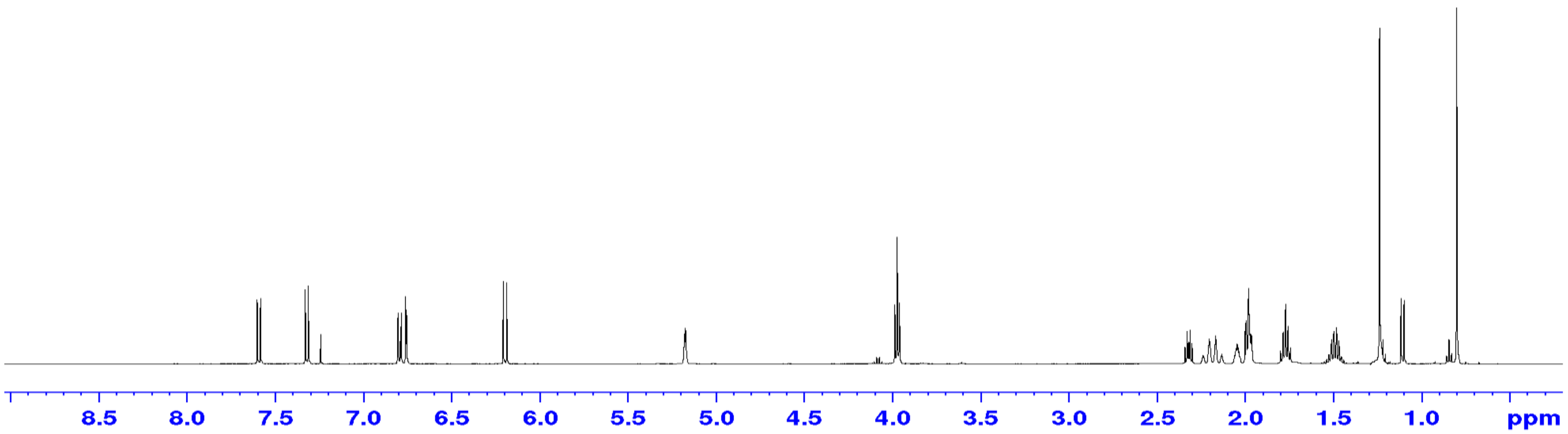

Compound **16g**

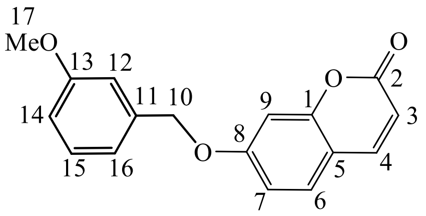

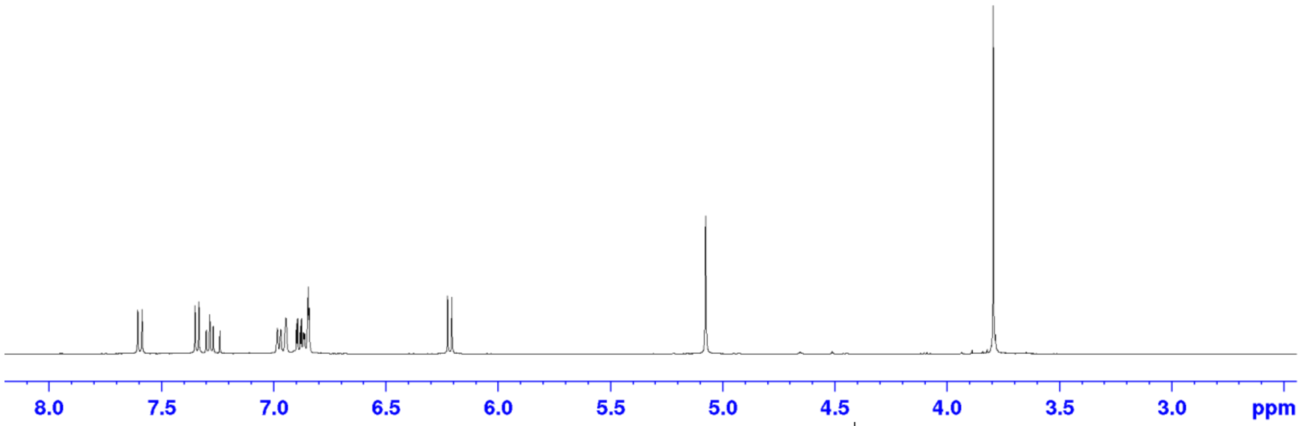

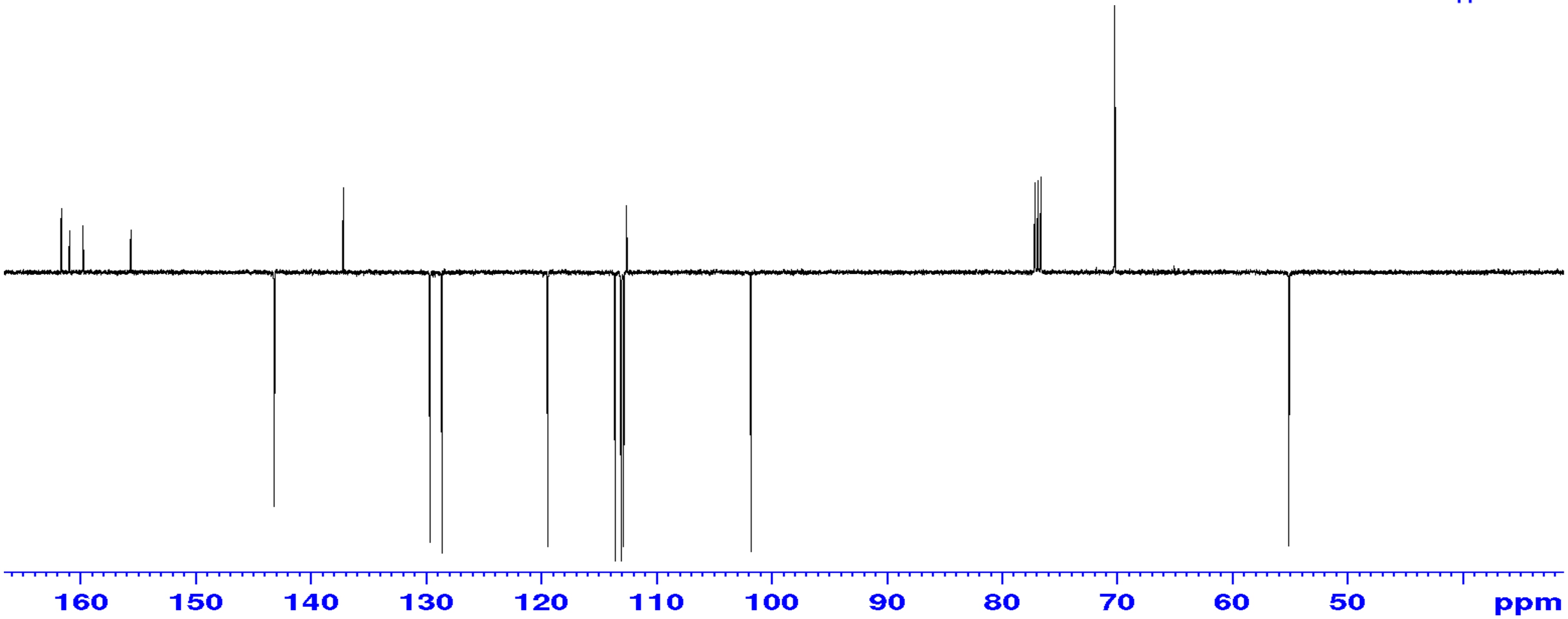

Compound **17a**

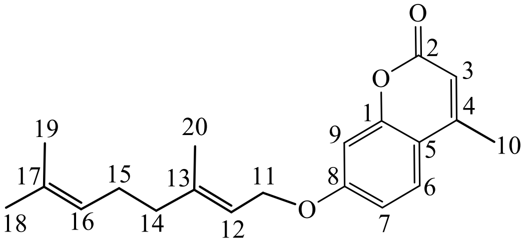

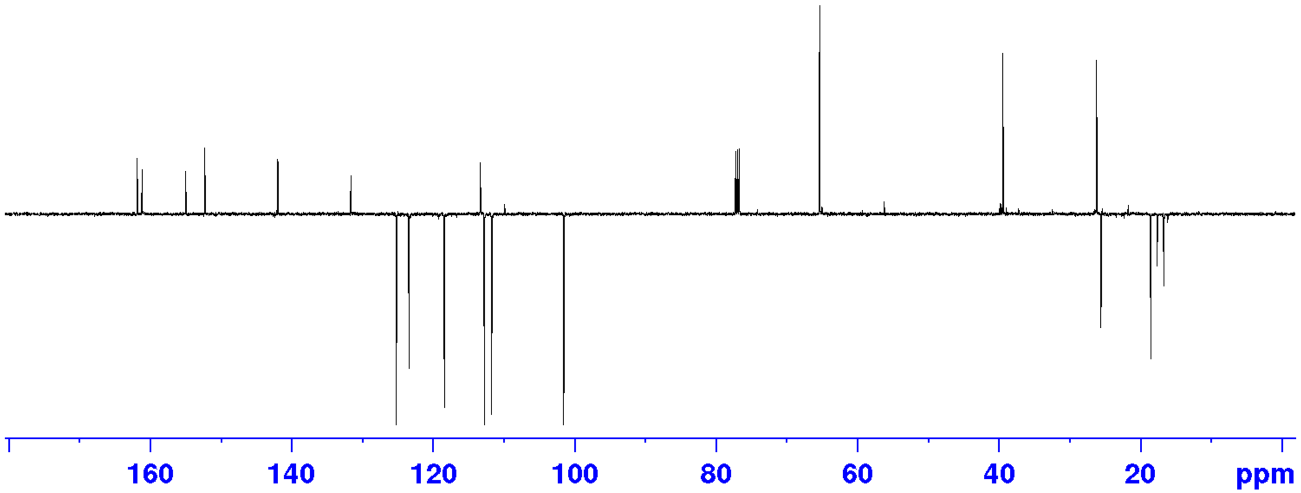

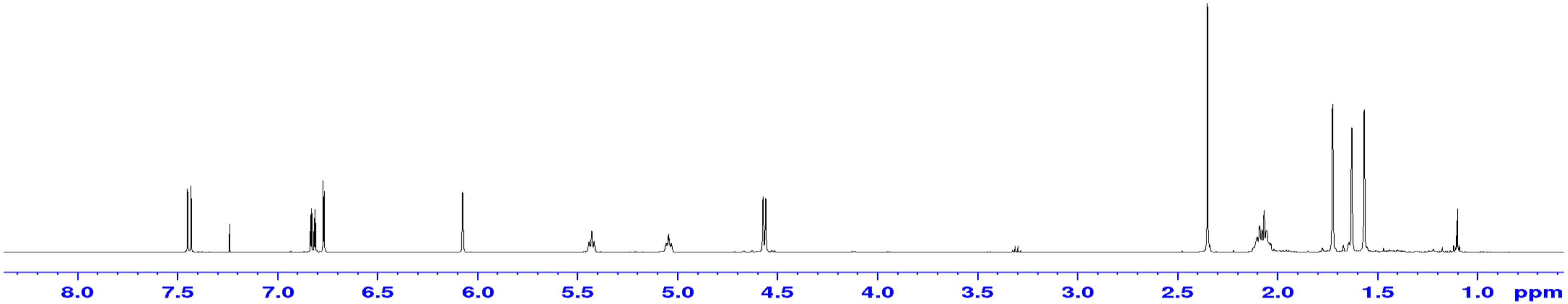

Compound **17c**

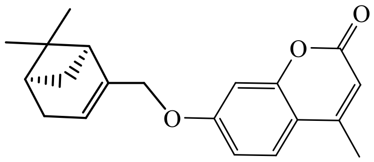

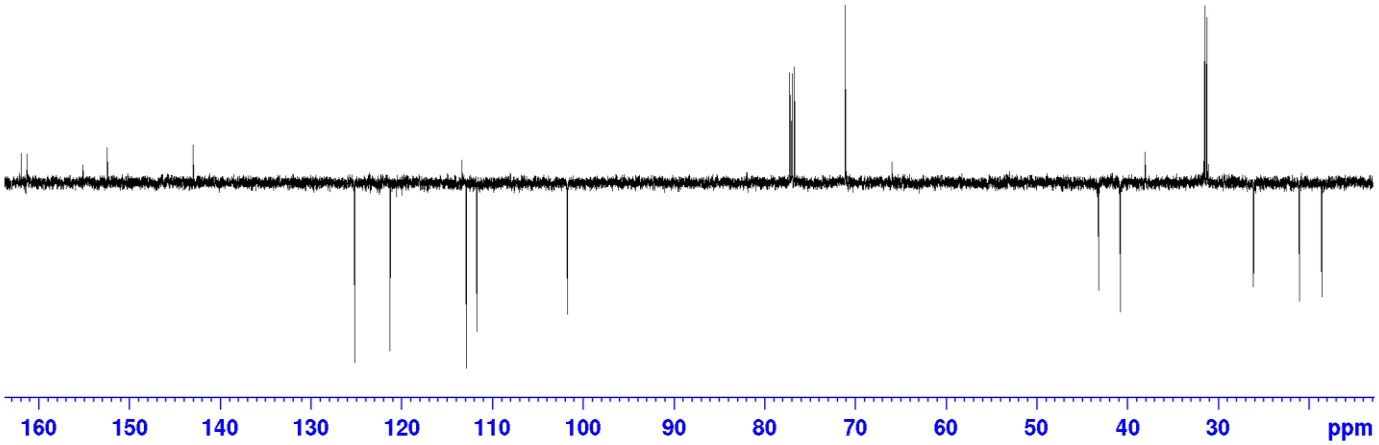

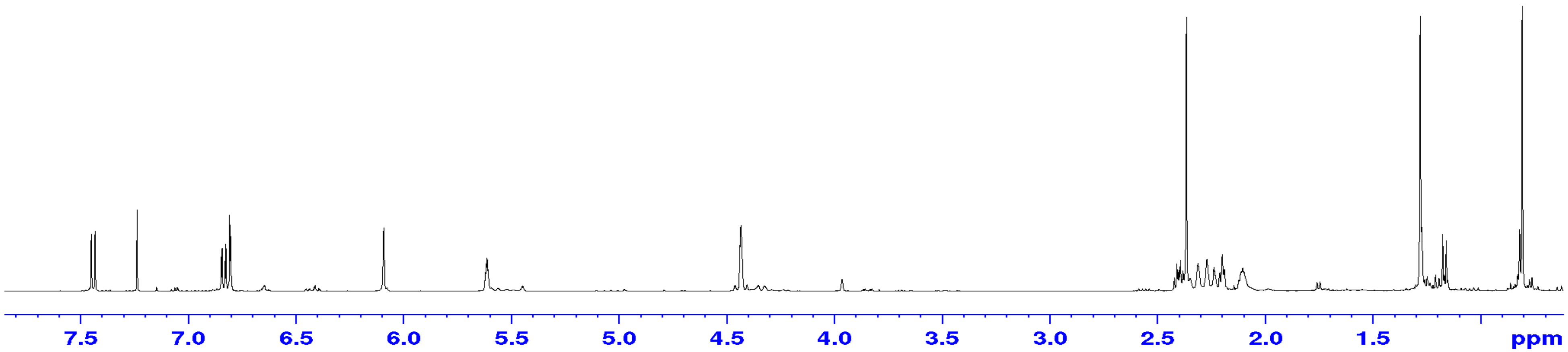

Compound **17e**

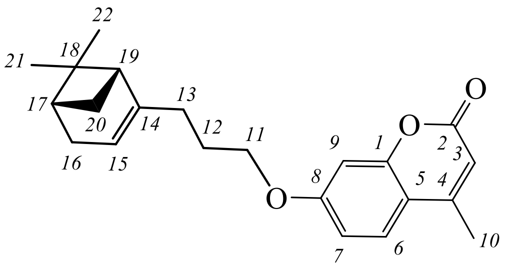

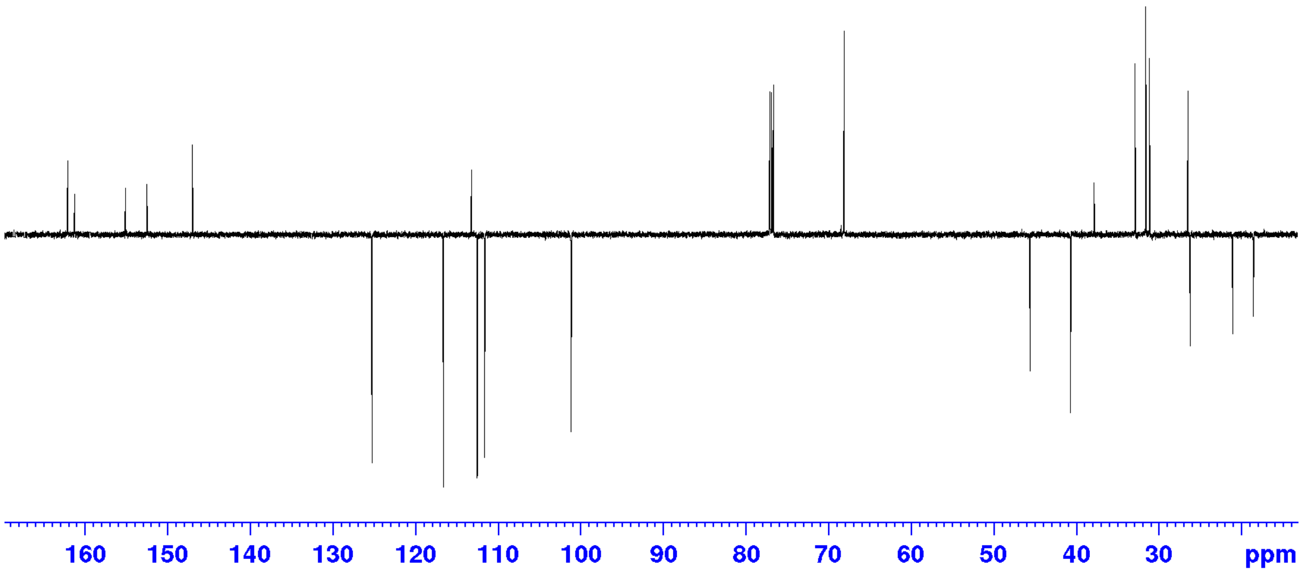

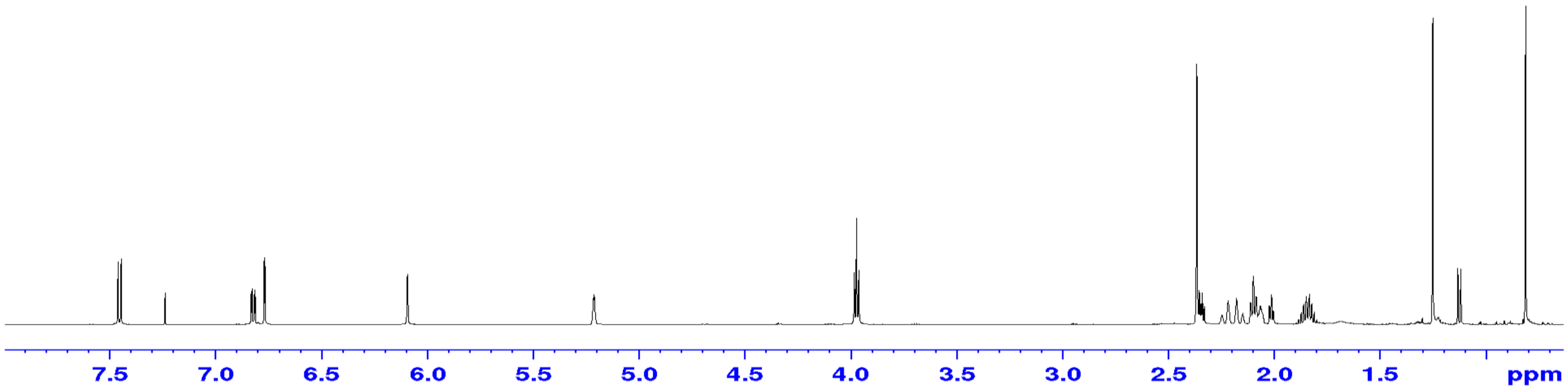

Compound **17f**

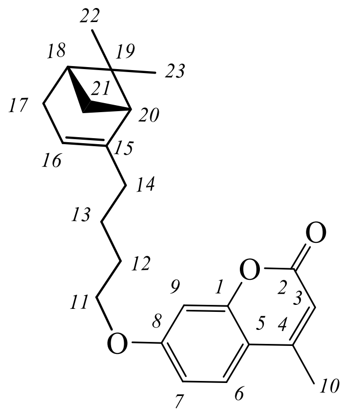

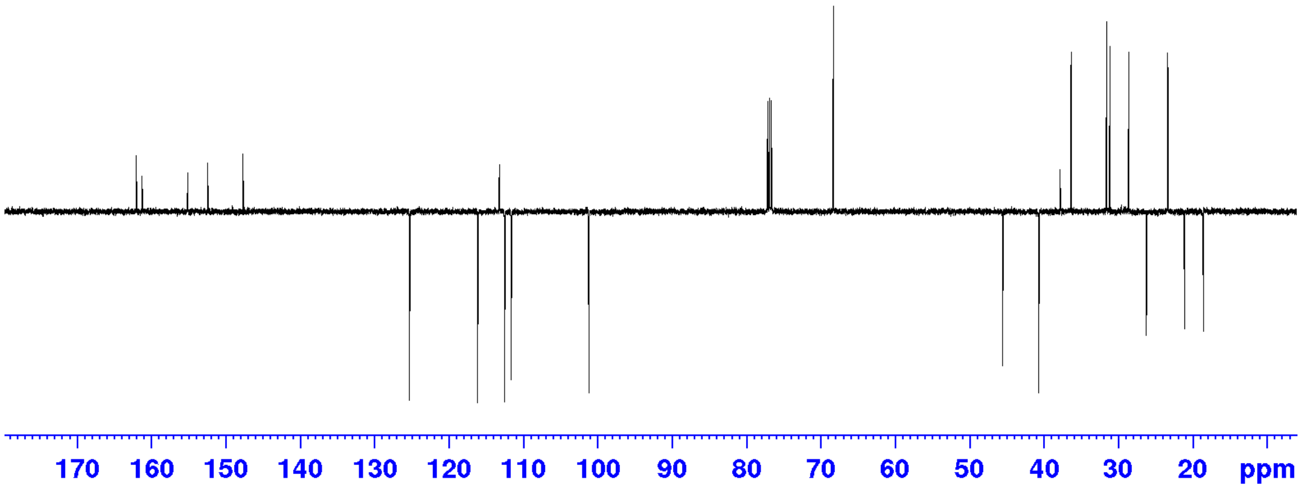

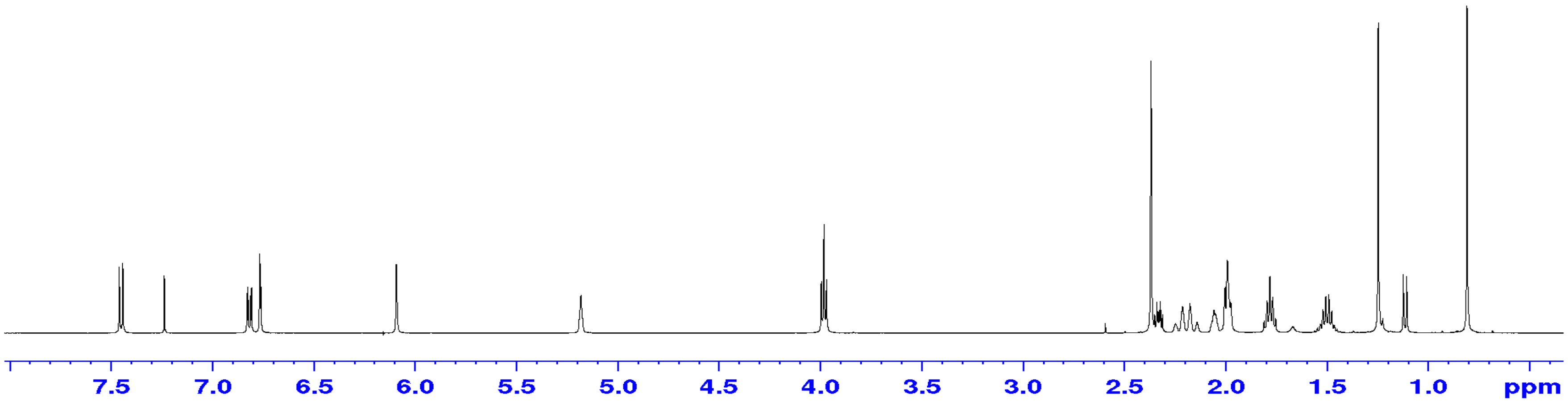

Compound **17g**

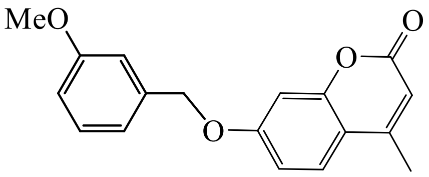

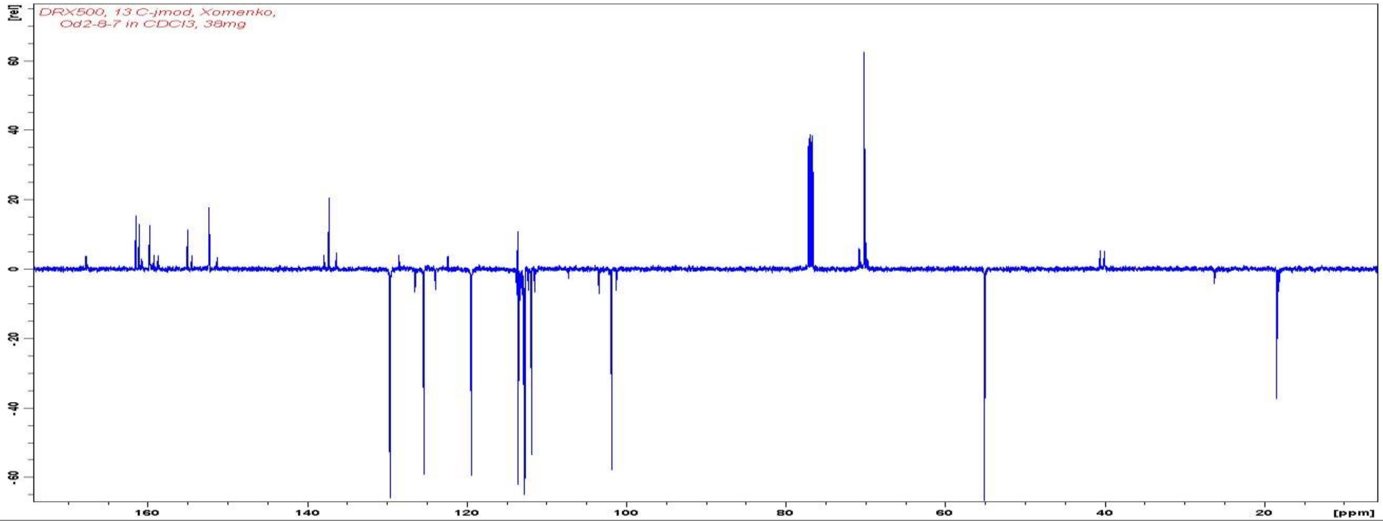

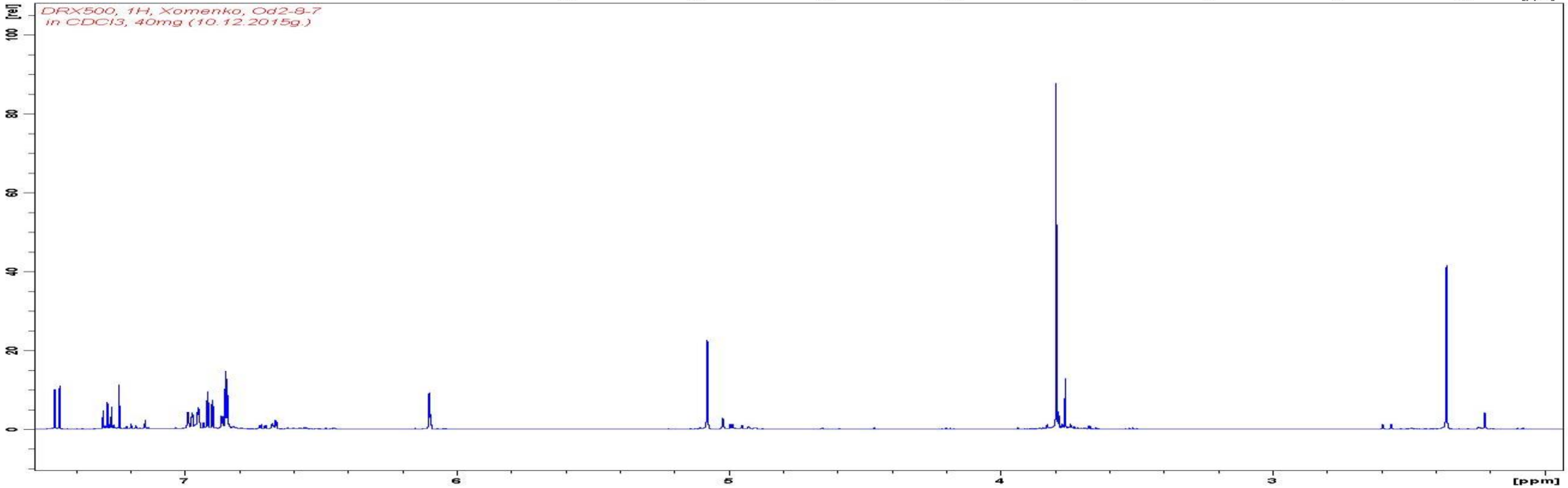

Compound **18a**

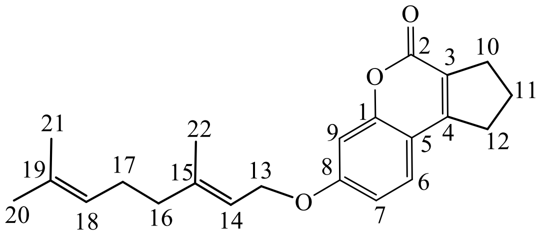

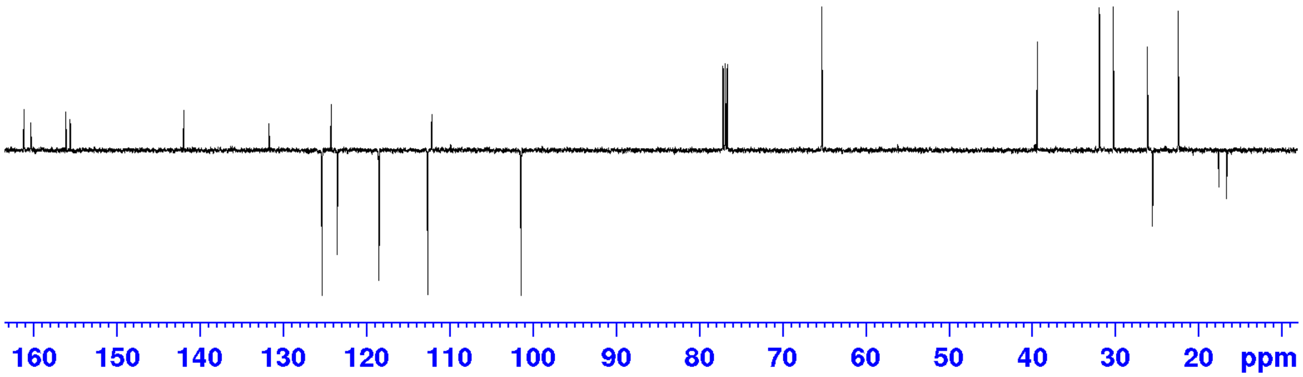

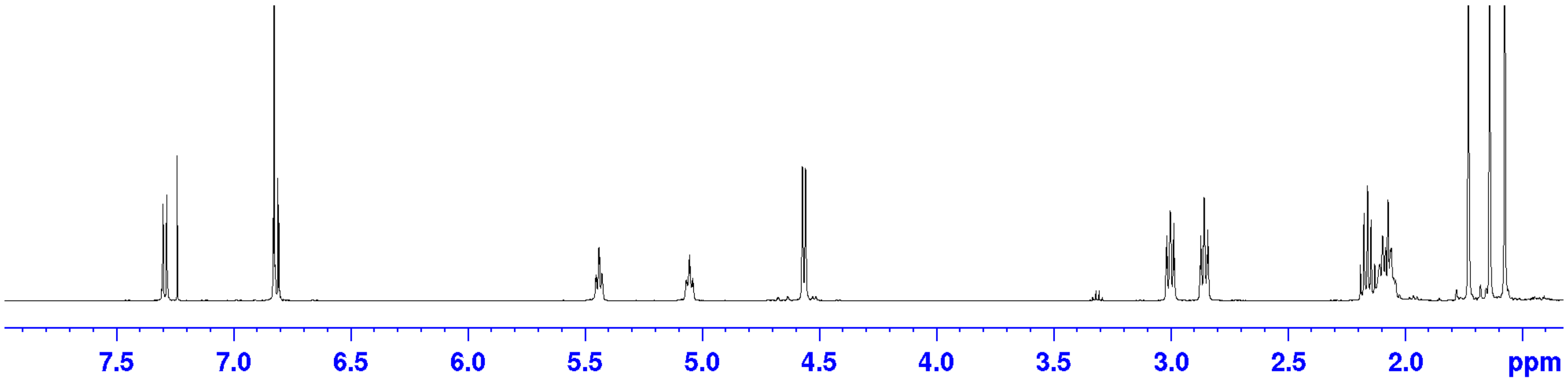

Compound **18c**

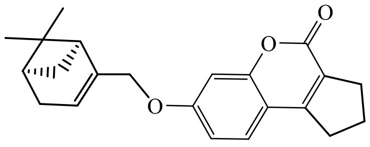

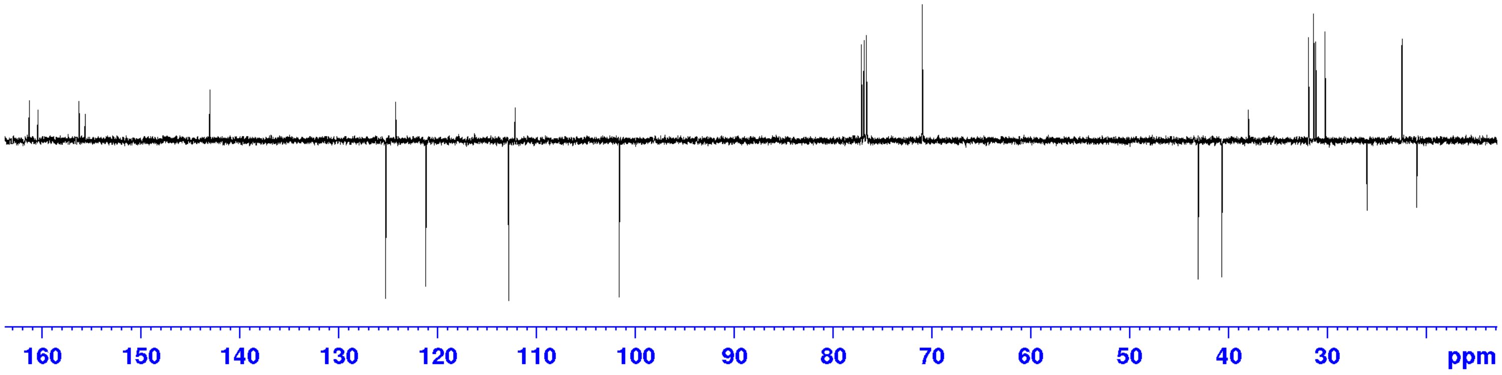

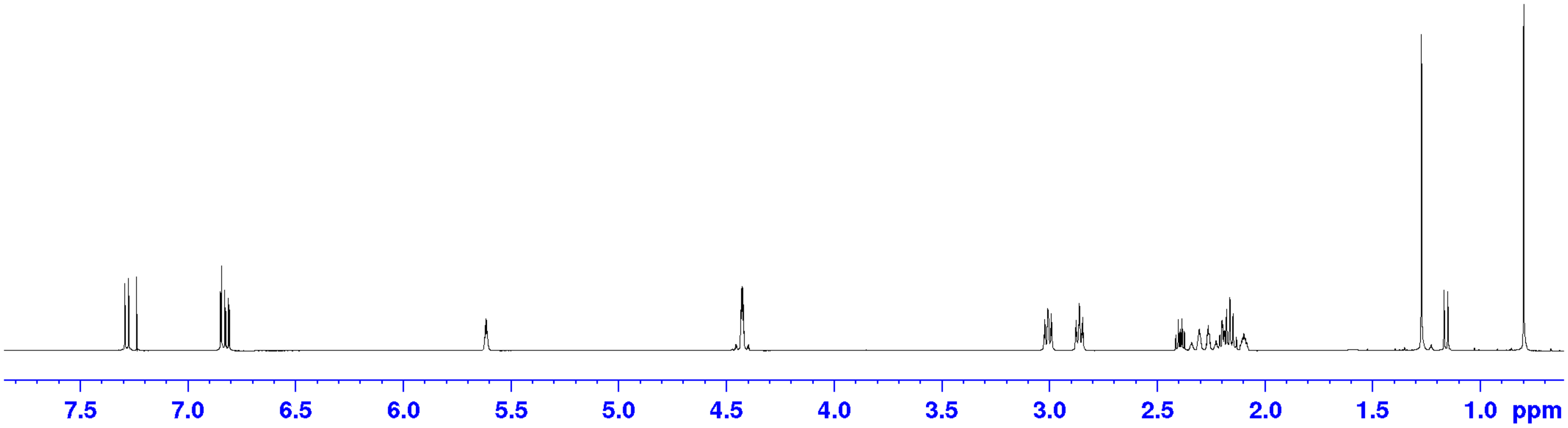

Compound **18d**

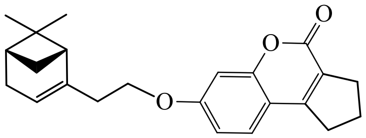

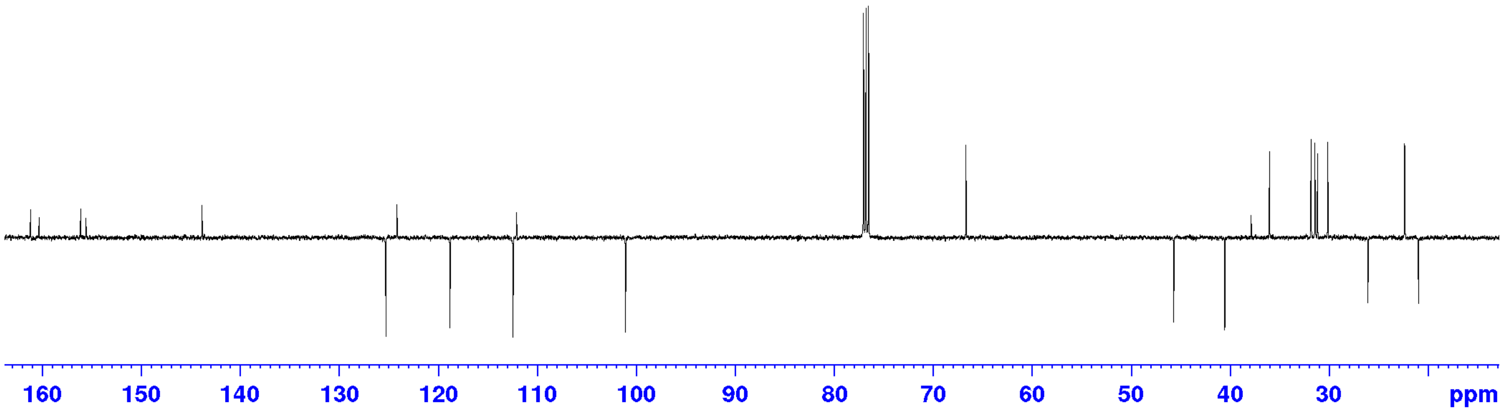

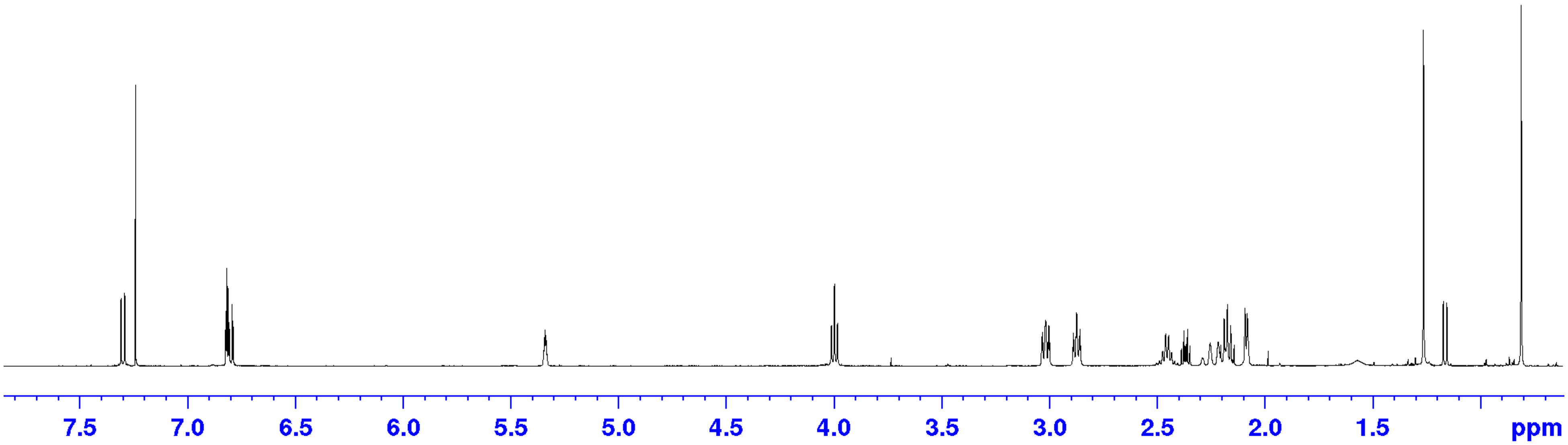

Compound **18e**

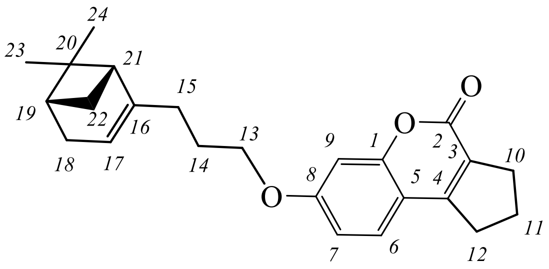

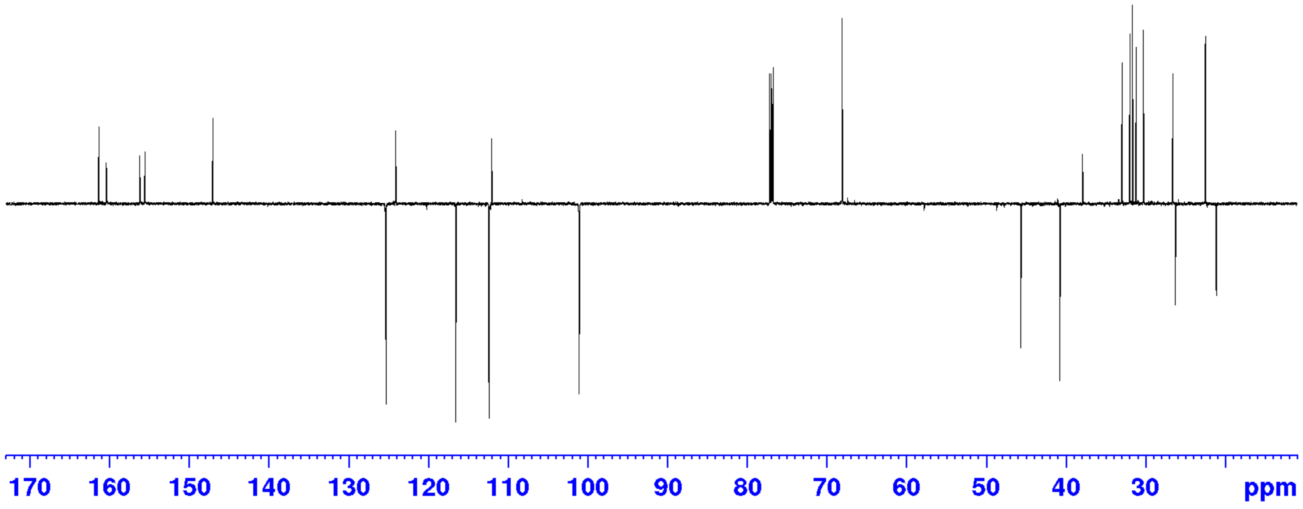

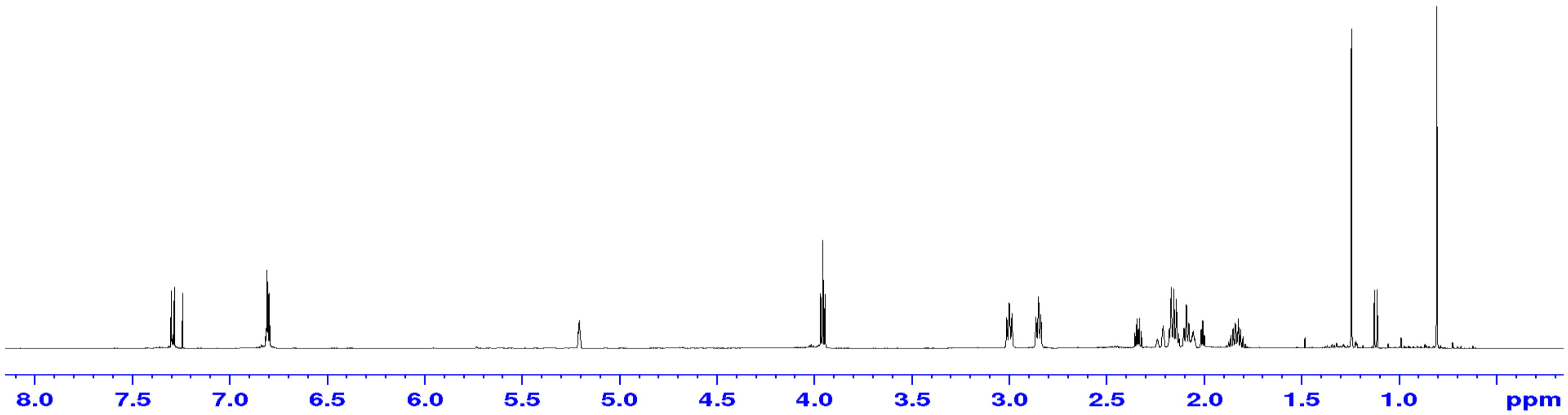

Compound **18f**

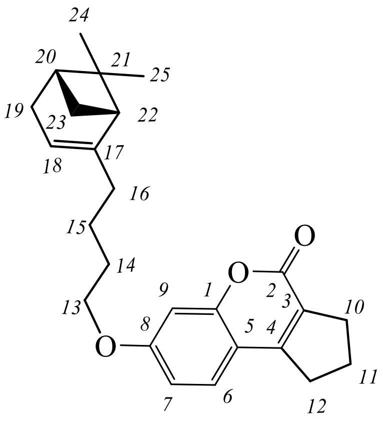

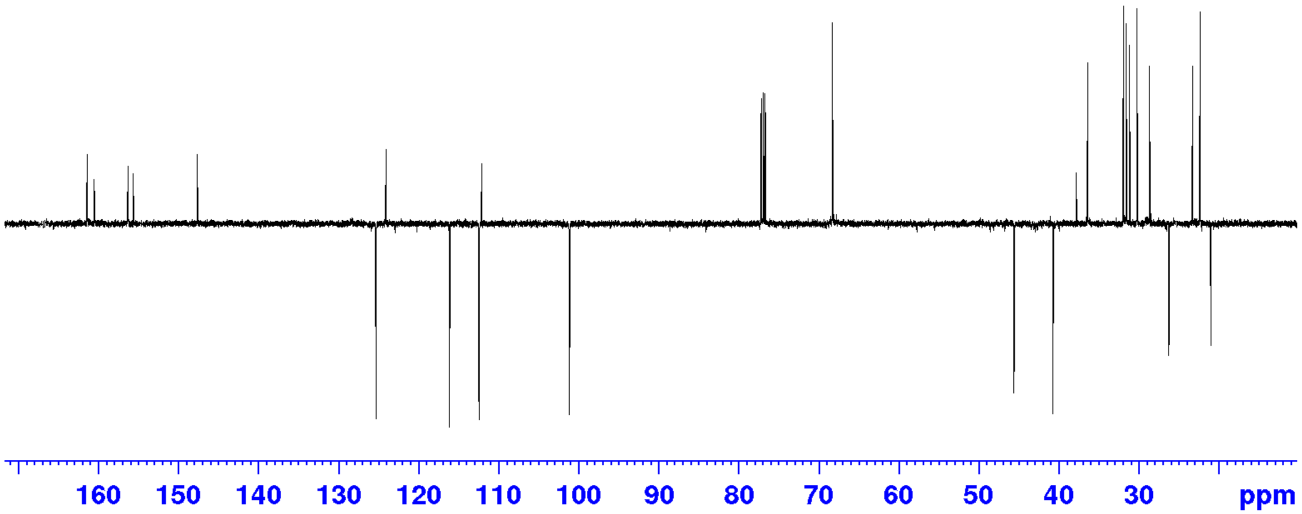

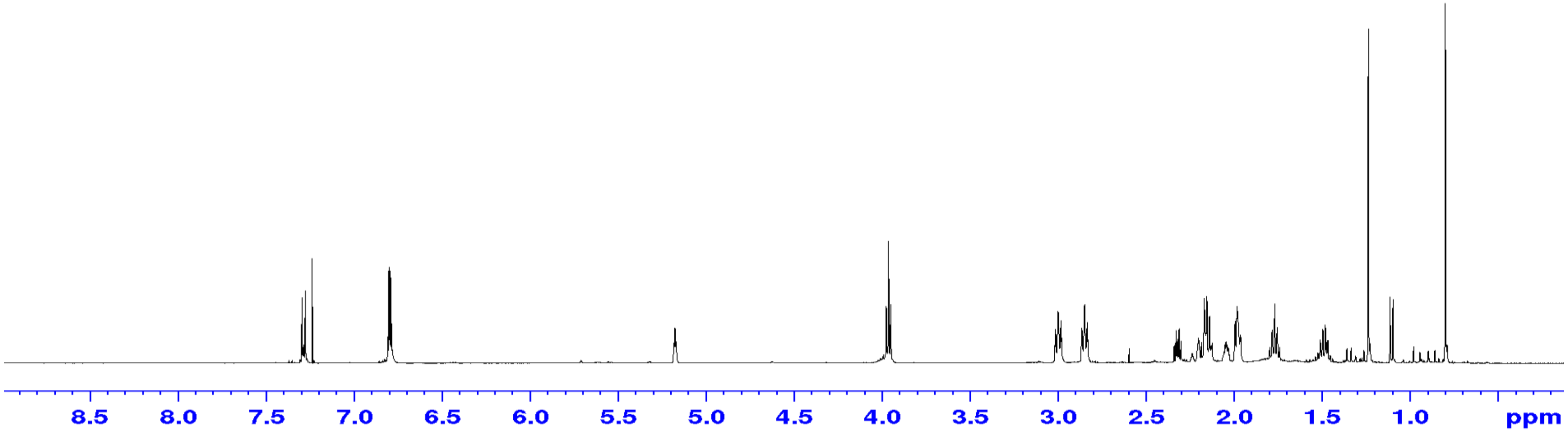

Compound **18g**

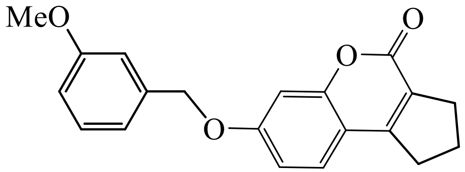

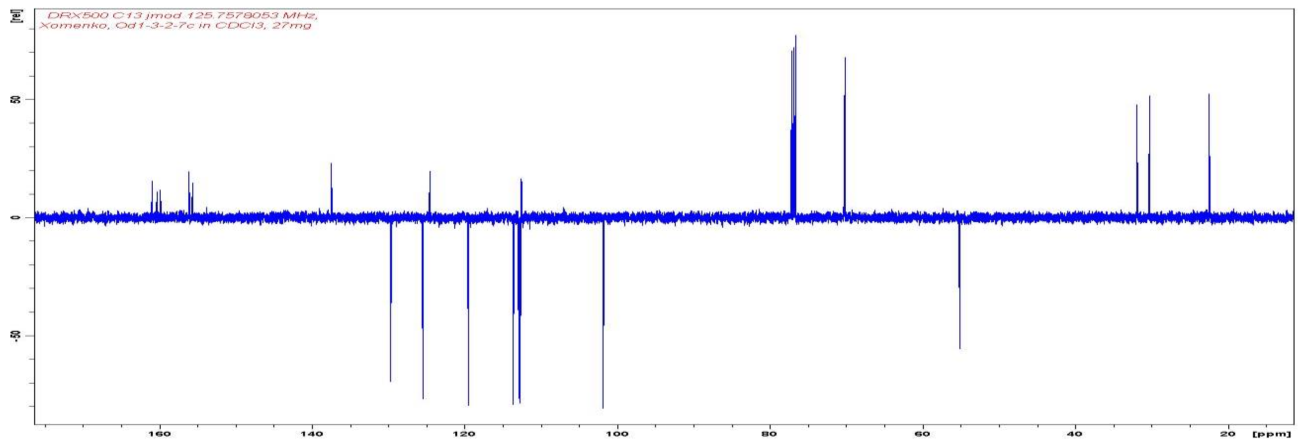

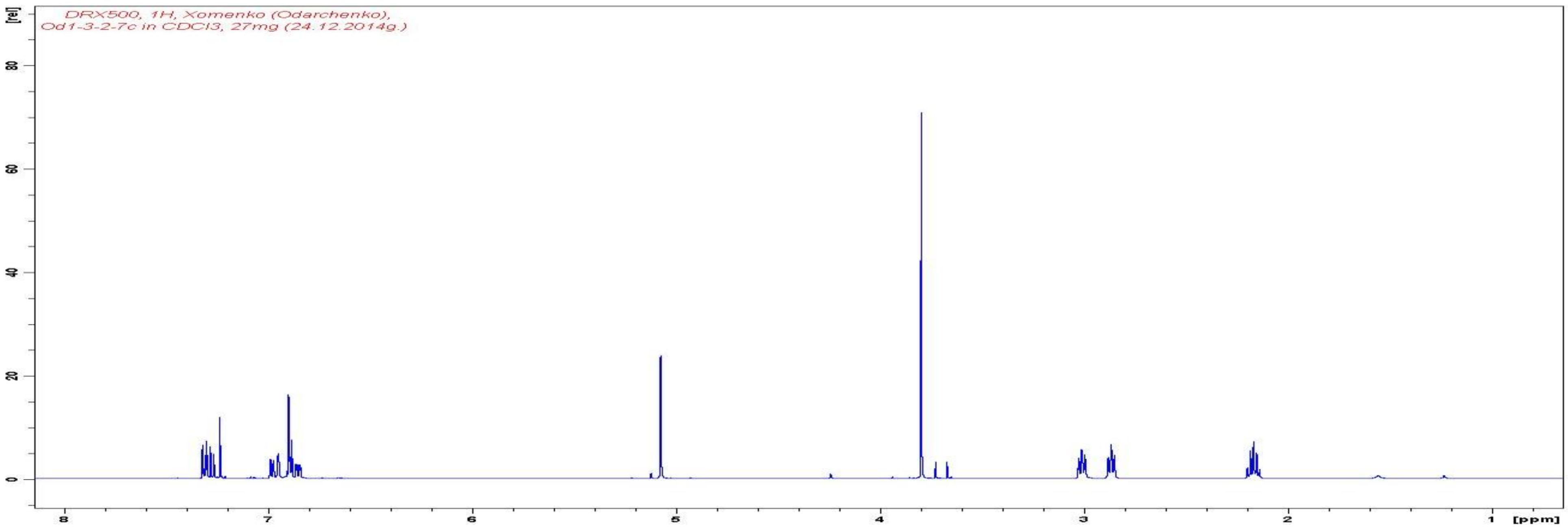

Compound **19a**

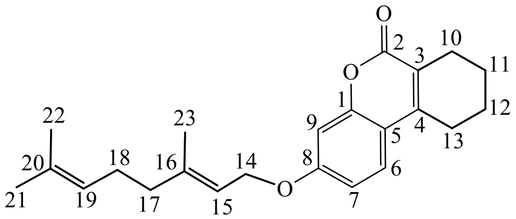

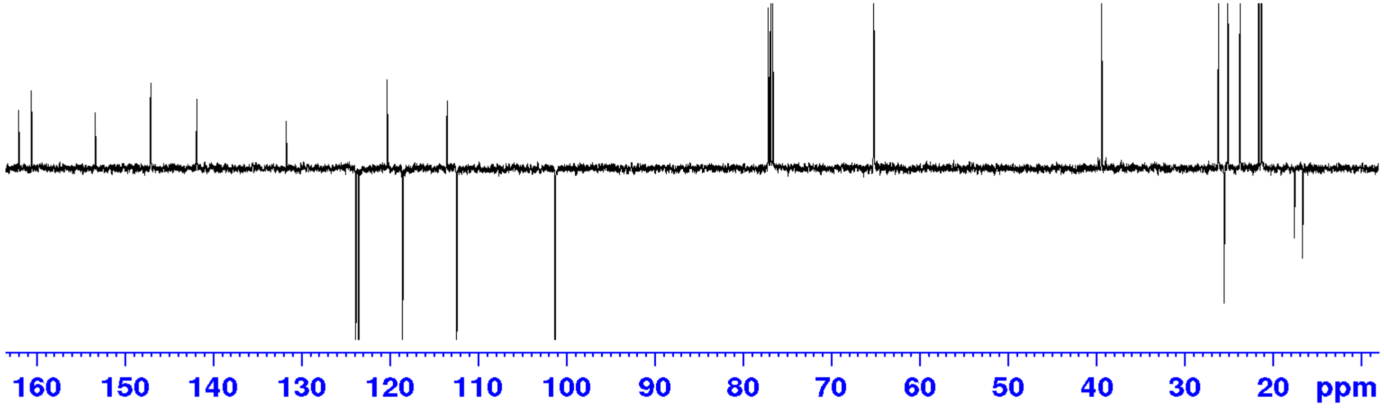

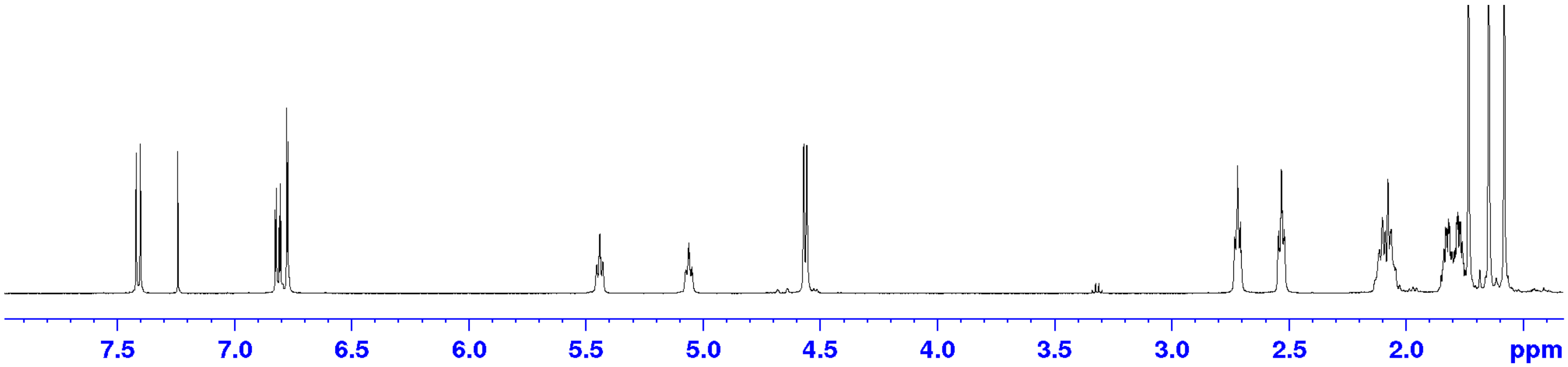

Compound **19c**

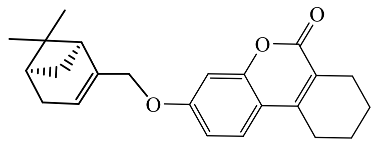

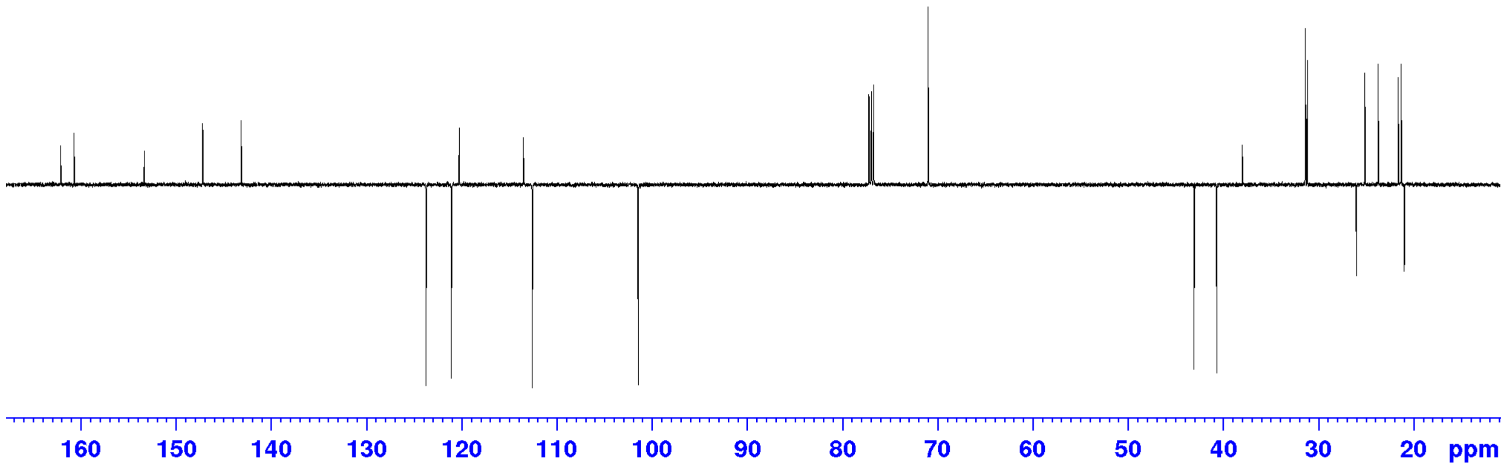

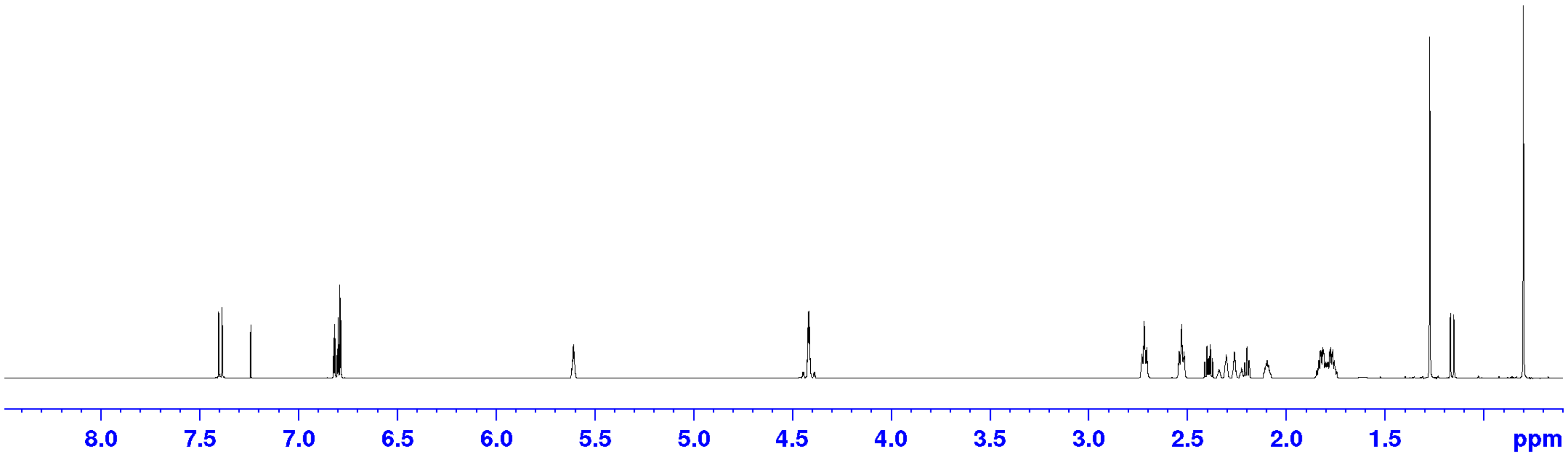

Compound **19d**

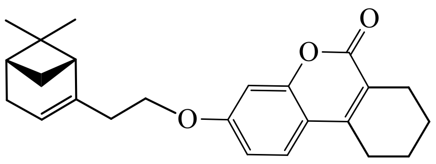

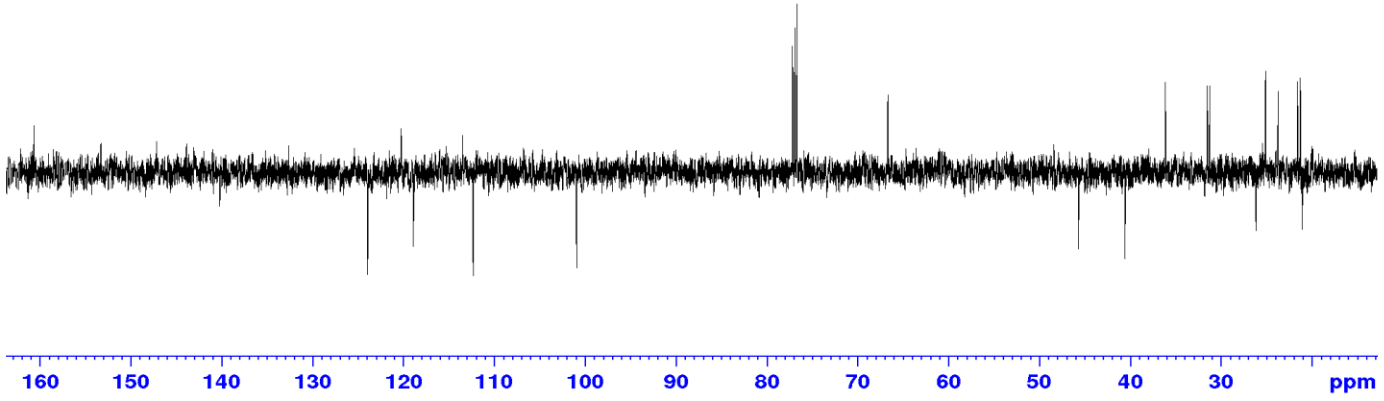

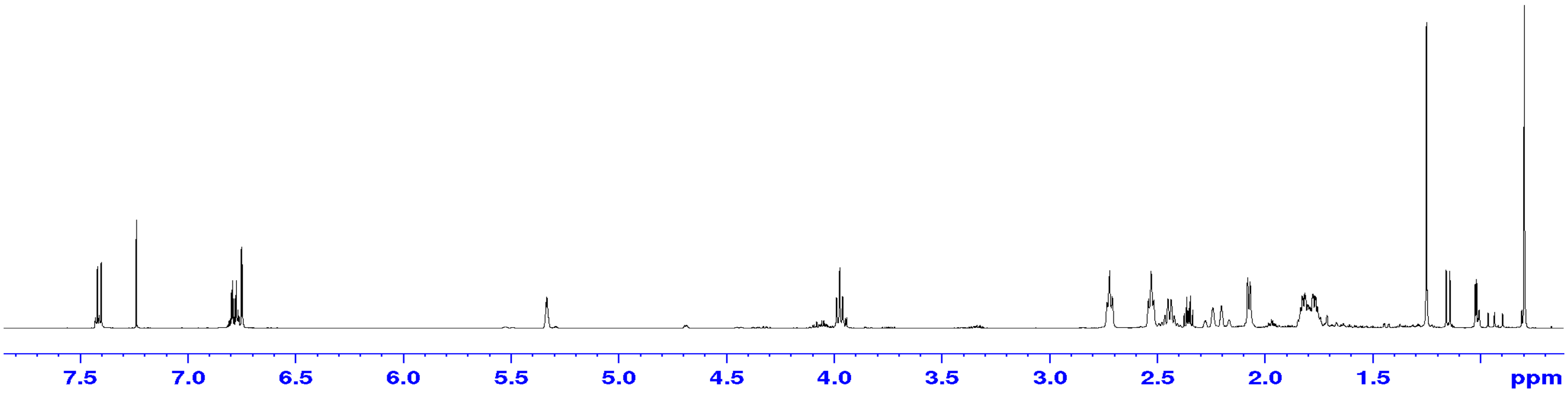

Compound **19e**

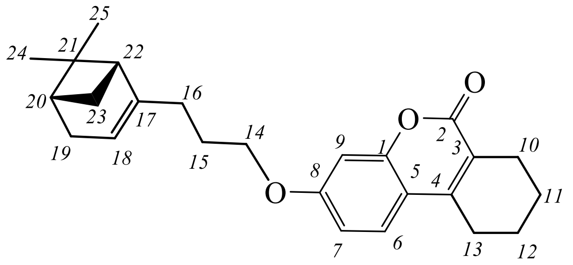

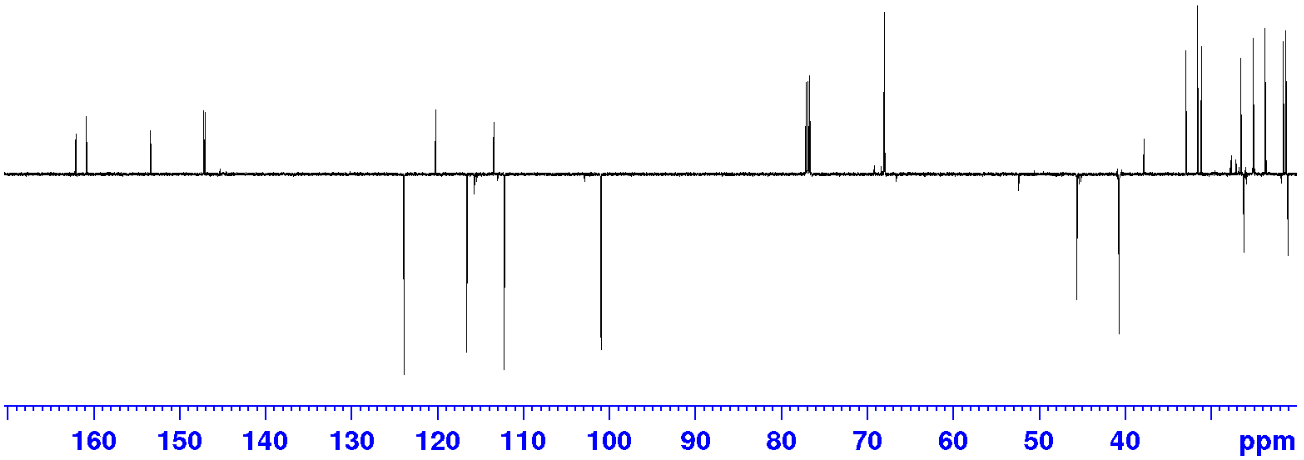
160 150 140 130 120 110 100 90 80 70 60 50 40 ppm

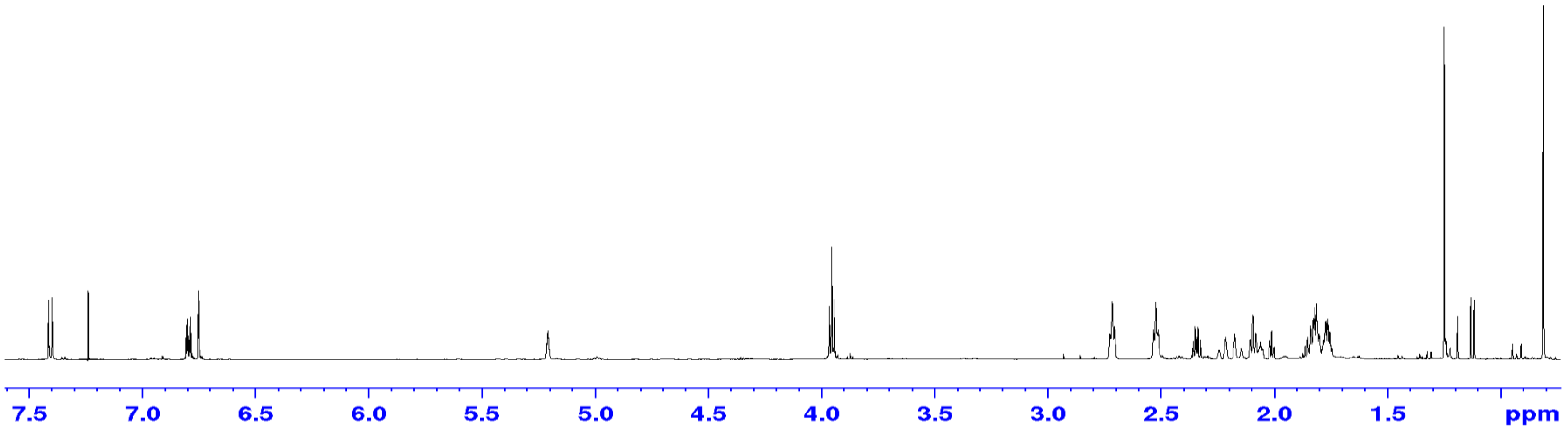
7.5 7.0 6.5 6.0 5.5 5.0 4.5 4.0 3.5 3.0 2.5 2.0 1.5 ppm

Compound **19f**

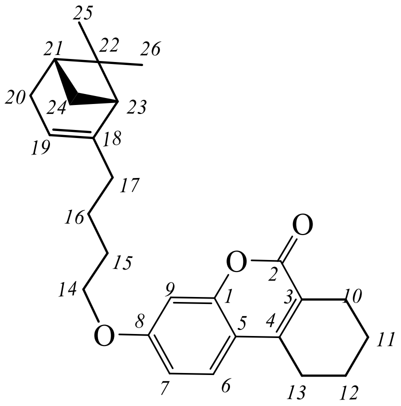

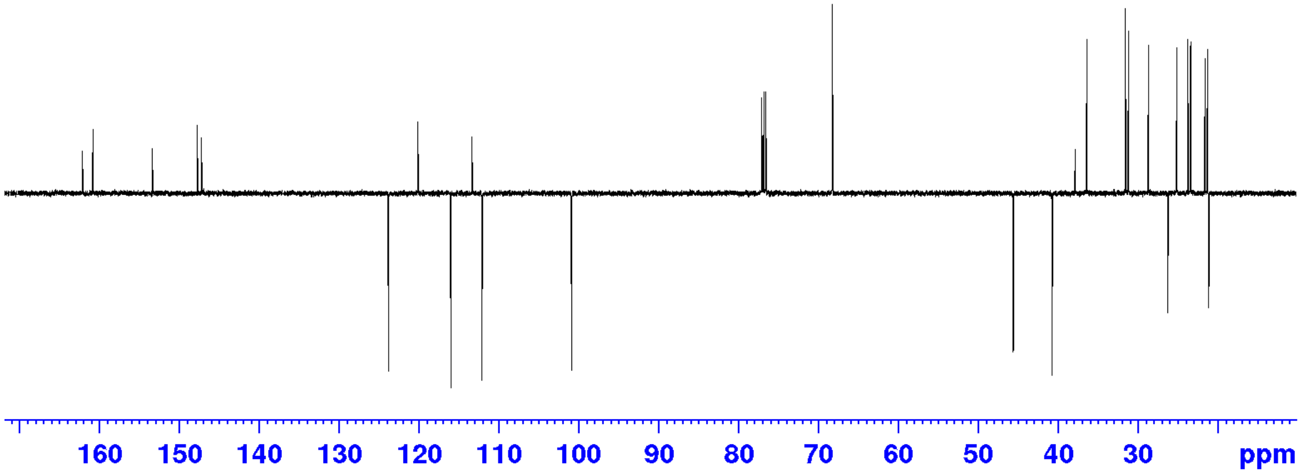

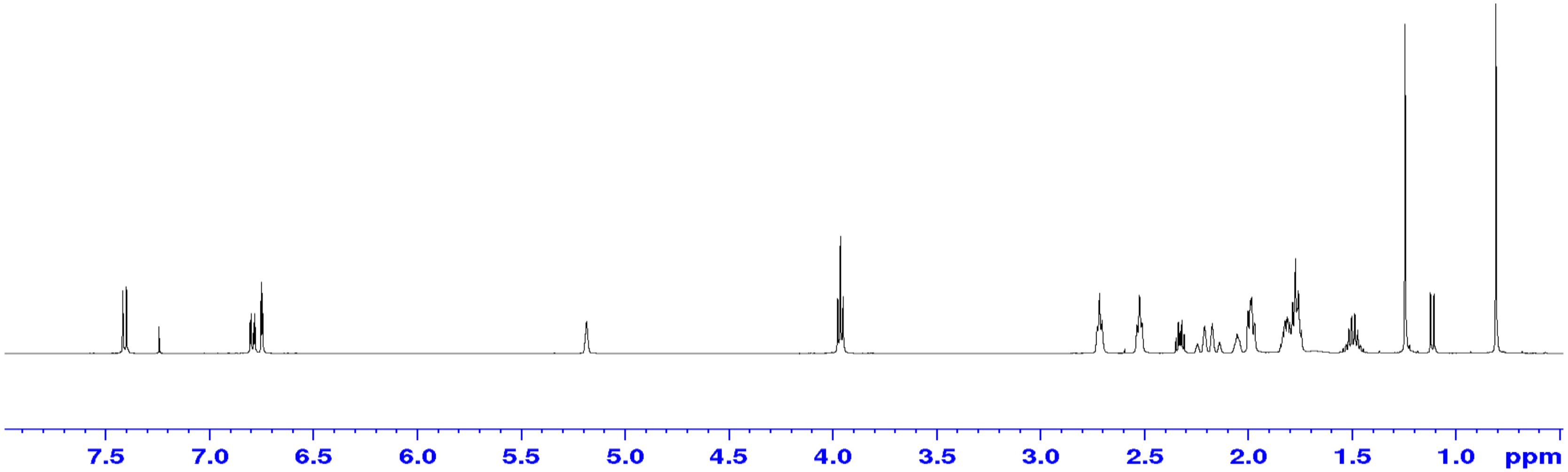

Compound **19g**

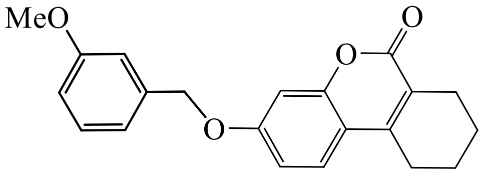

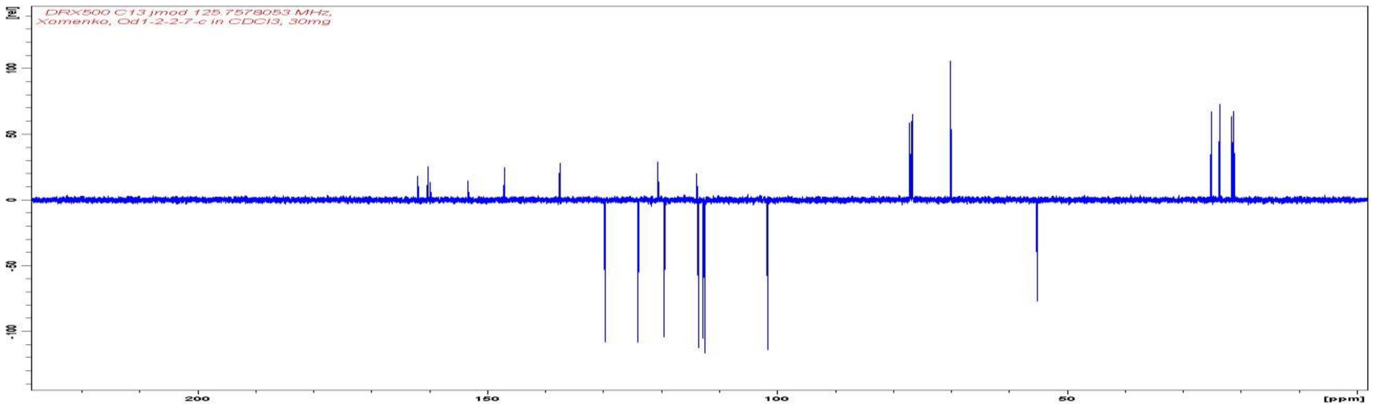

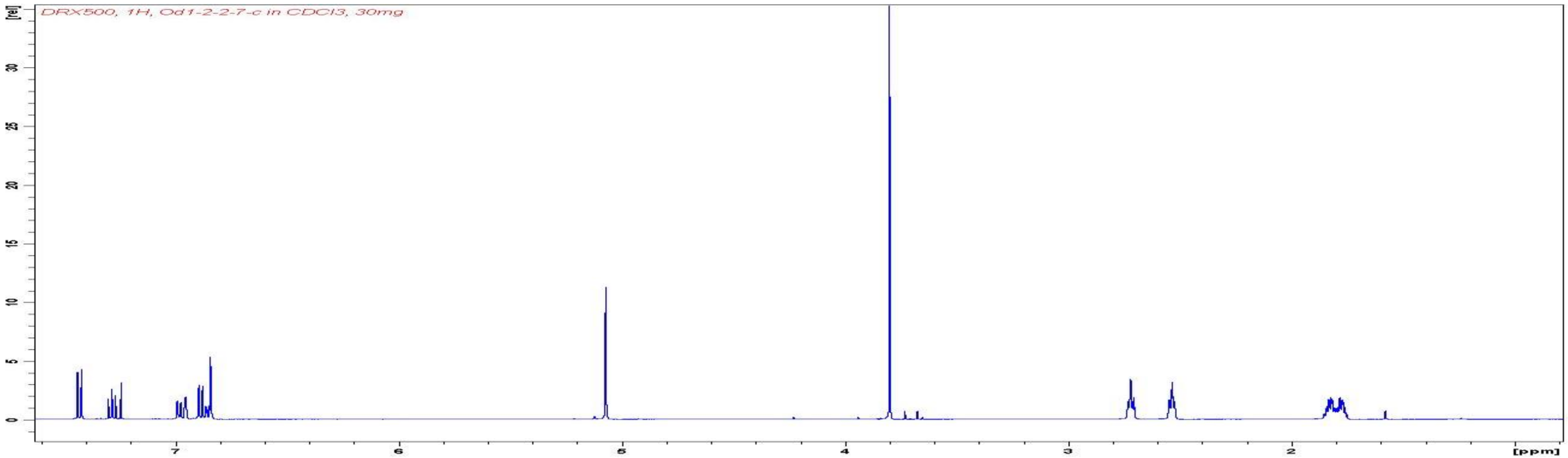

Compound **25**

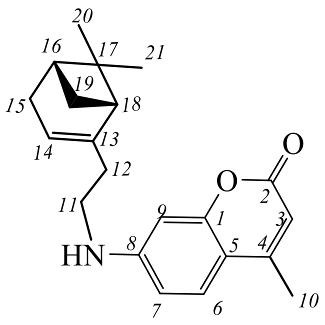
20
16
17
21
19
15
18
13
14
12
O
O
2
11
9
1
3
HN
8
5
4
7
6
10

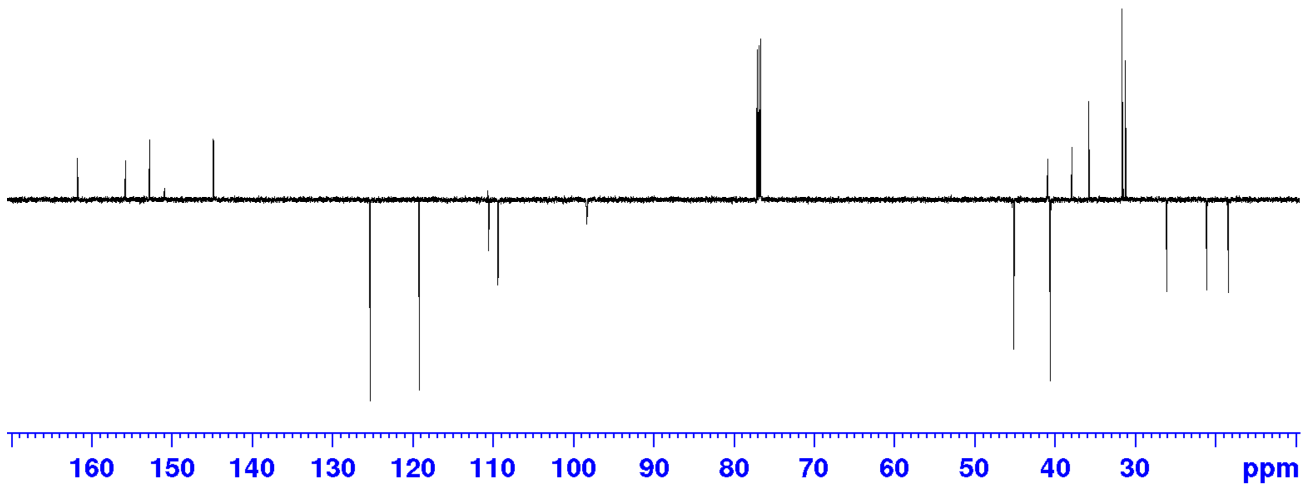
160 150 140 130 120 110 100 90 80 70 60 50 40 30 ppm

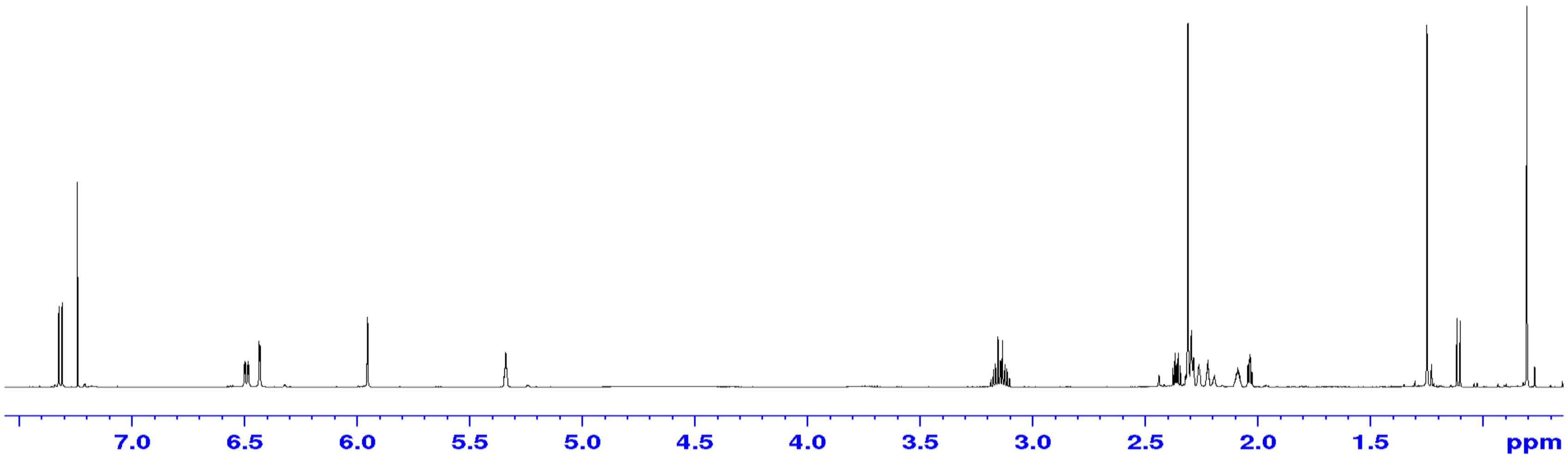
7.0 6.5 6.0 5.5 5.0 4.5 4.0 3.5 3.0 2.5 2.0 1.5 ppm

Compound **27**

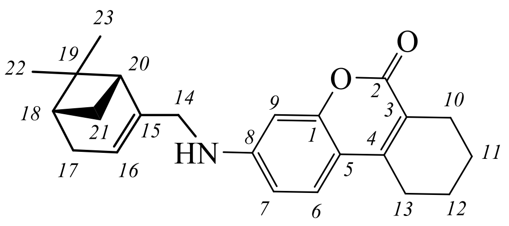
23
22
19
20
18
21
15
14
17
16
HN
9
8
1
O
2
O
3
10
11
4
5
7
6
13
12

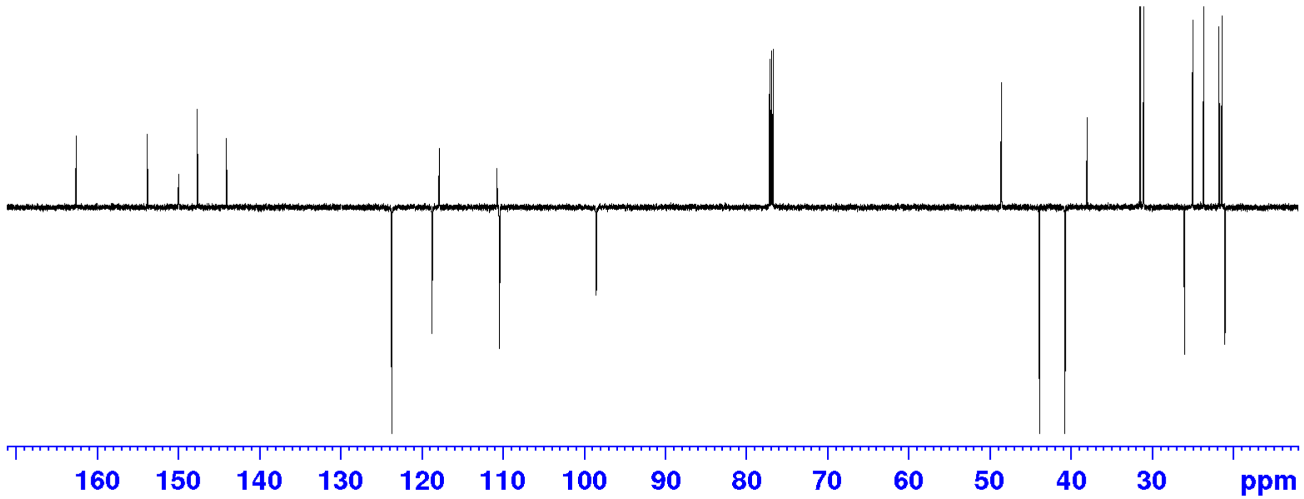
160
150
140
130
120
110
100
90
80
70
60
50
40
30
ppm

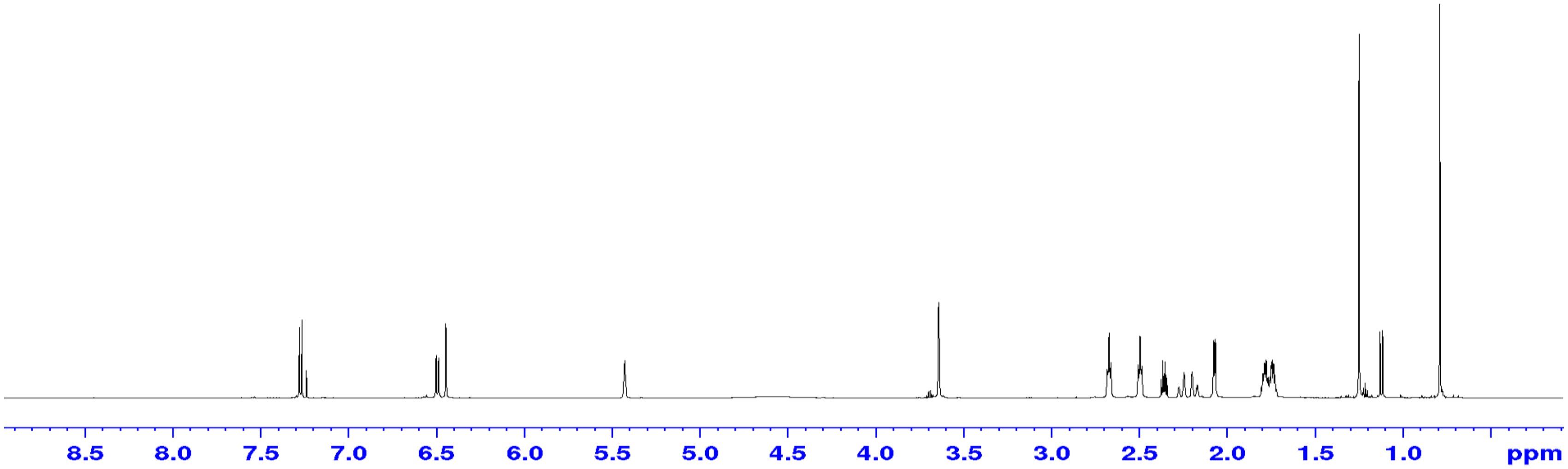
8.5
8.0
7.5
7.0
6.5
6.0
5.5
5.0
4.5
4.0
3.5
3.0
2.5
2.0
1.5
1.0
ppm

Table S1 Energy parameters of docking study procedure

Compounds	Number of pose (max 20)	|LE| (range)	Glide score (range)	Emodel (range)	IFD score (range)	Amino acids (For best position)	Type of interactions (For best position)
Sisunatovir (Figure – SM1)	20	0.351 (0.310 – 0.351)	-11.236 (-9.924 – - 11.236)	-112.836 (-92.886 – - 112.836)	-2963.43 (-2961.15 – - 2963.43)	A: Asp486<br>B: Glu487	Salt bridge
						B: Asp486	H-bond
						A: Phe488<br>B: Phe488<br>B: Phe140	π-π
19c (Figure – SM2)	20	0.368 (0.323 – 0.368)	-9.567 (-8.403 – -9.567)	-70.740 (-70.740 – - 74.951)	-2960.50 (-2958.93 – - 2960.50)	A: Phe488	H-bond
						A: Phe488<br>D: Phe488	π-π
19f (Figure – SM3)	19	0.318 (0.240 – 0.327)	-9.233 (-6.973 – -9.233)	-71.713 (-54.428 – 71.713)	-2961.78 (-2959.07 – - 2961.78)	A: Phe488<br>A: Asp489	H-bond
						D: Phe488	π-π
19h (Figure – SM4)	15	0.367 (0.317 – 0.367)	-8.433 (-7.297 – -8.591)	-60.551 (-53.183 – - 63.834)	-2960.72 (-2959.62 – - 2960.72)	A: Phe488	H-bond
						B: Phe488	π-π

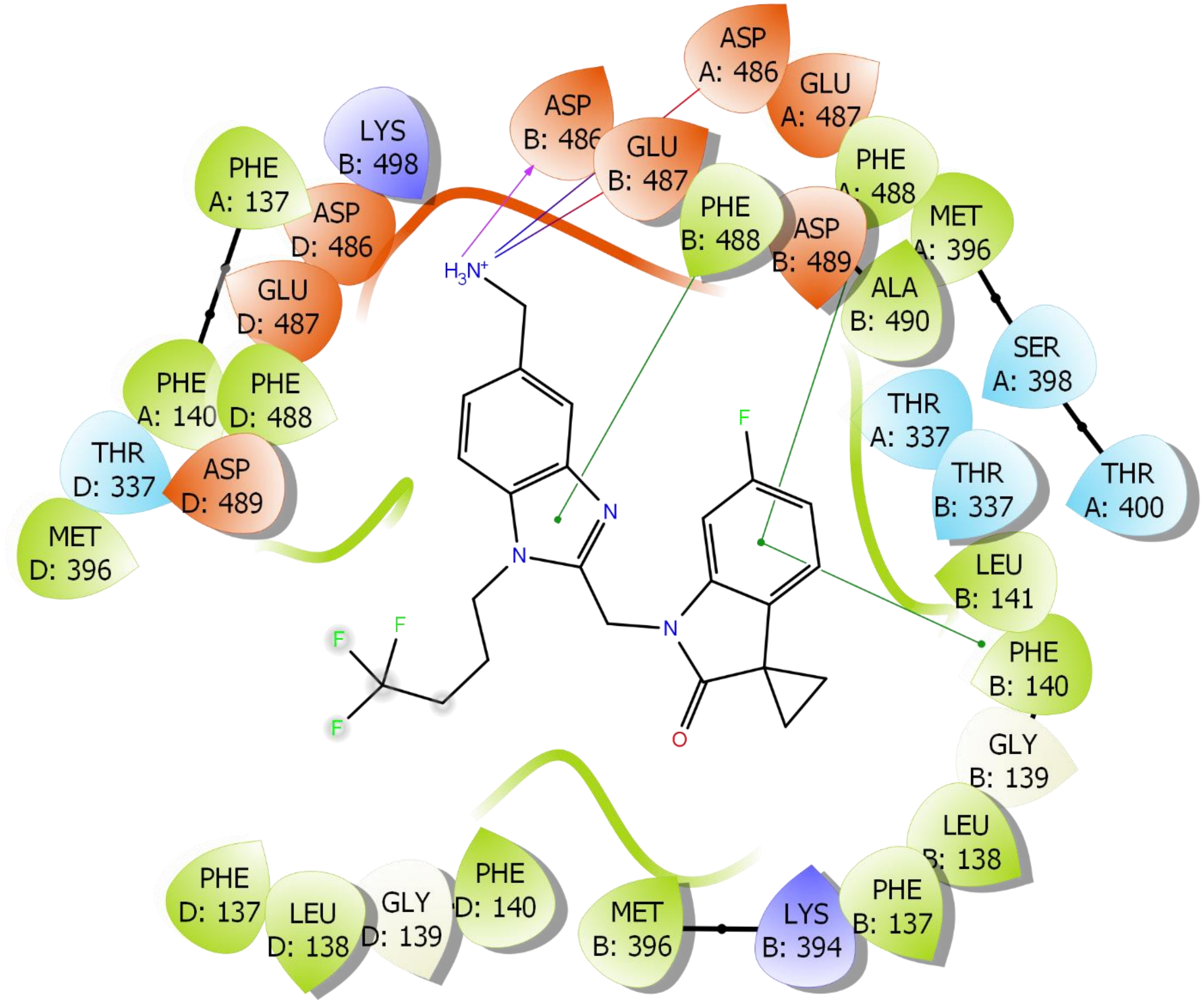

Figure S1 – best position of Sisunatovir.

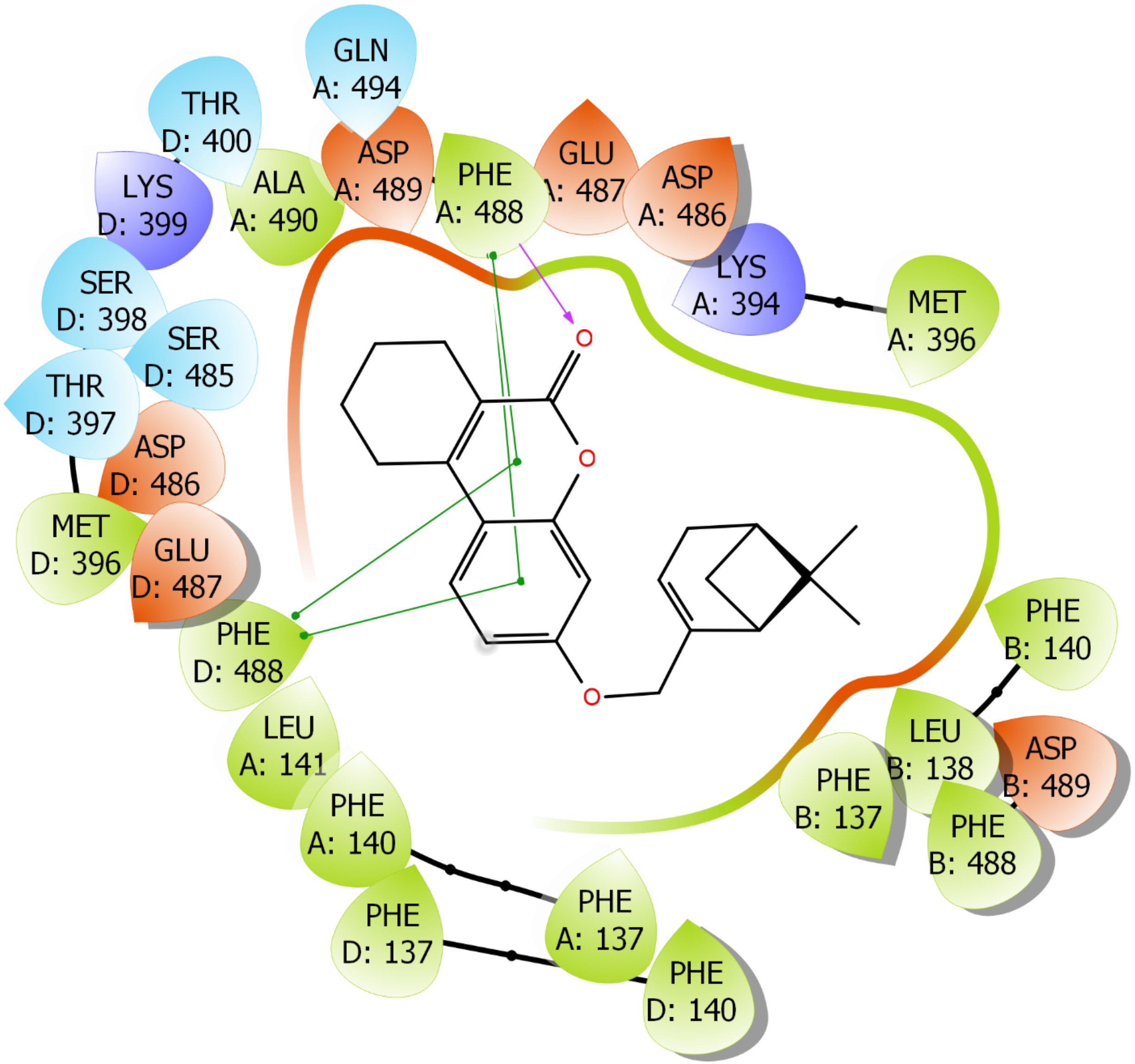

Figure S2 – best position of **19c**

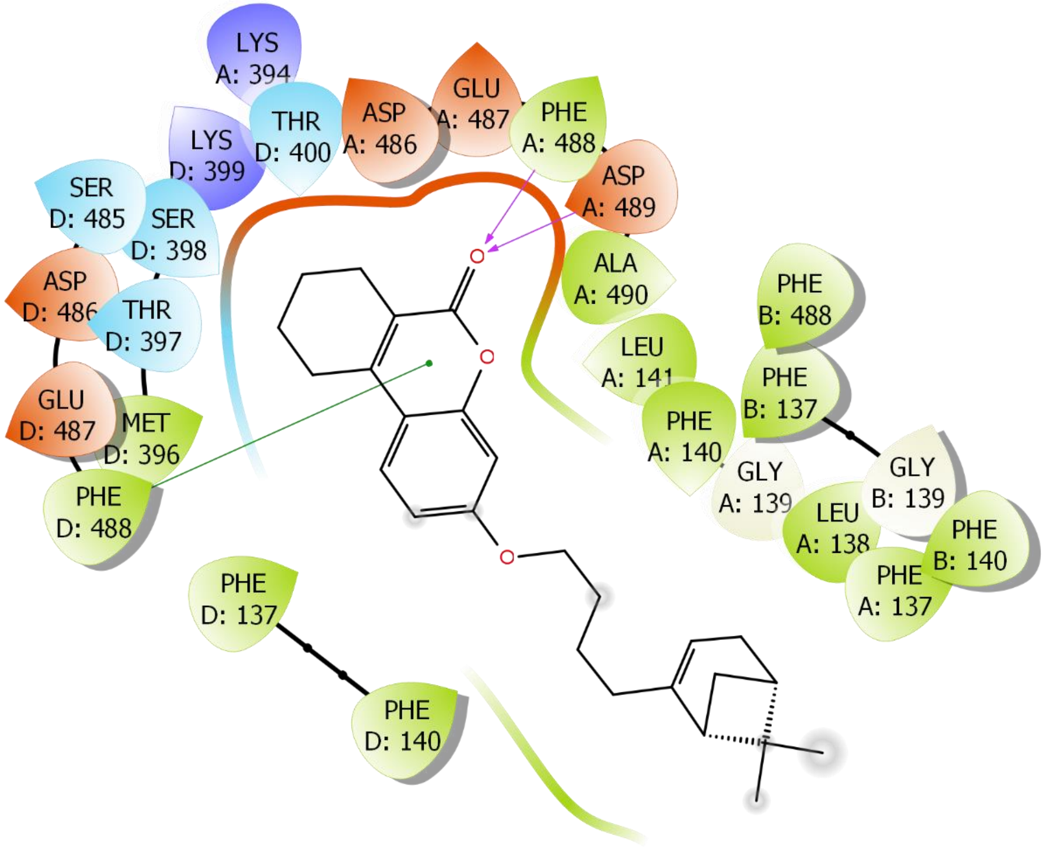

Figure S3 – best position of **19f**

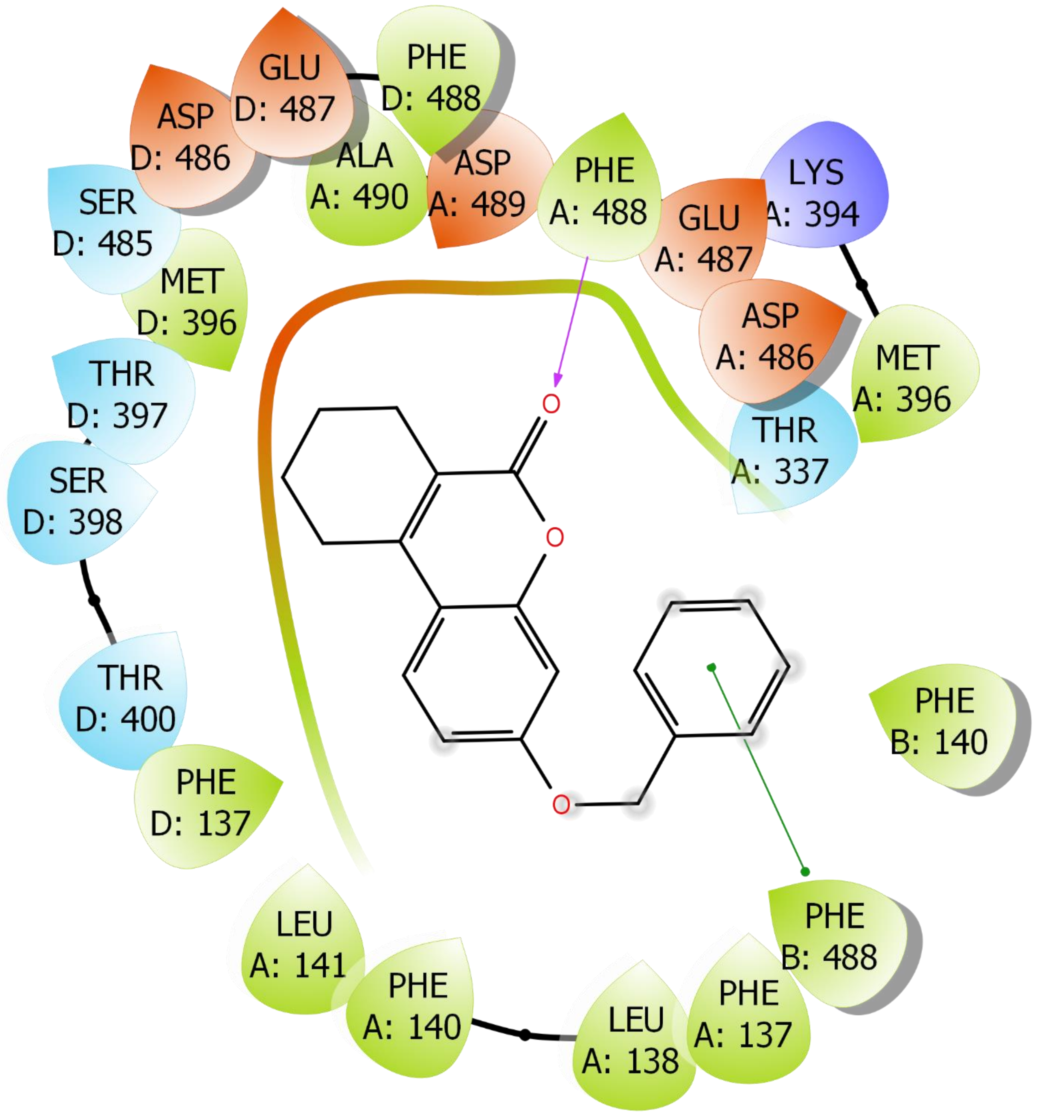

Figure S4 – best position of **19h**

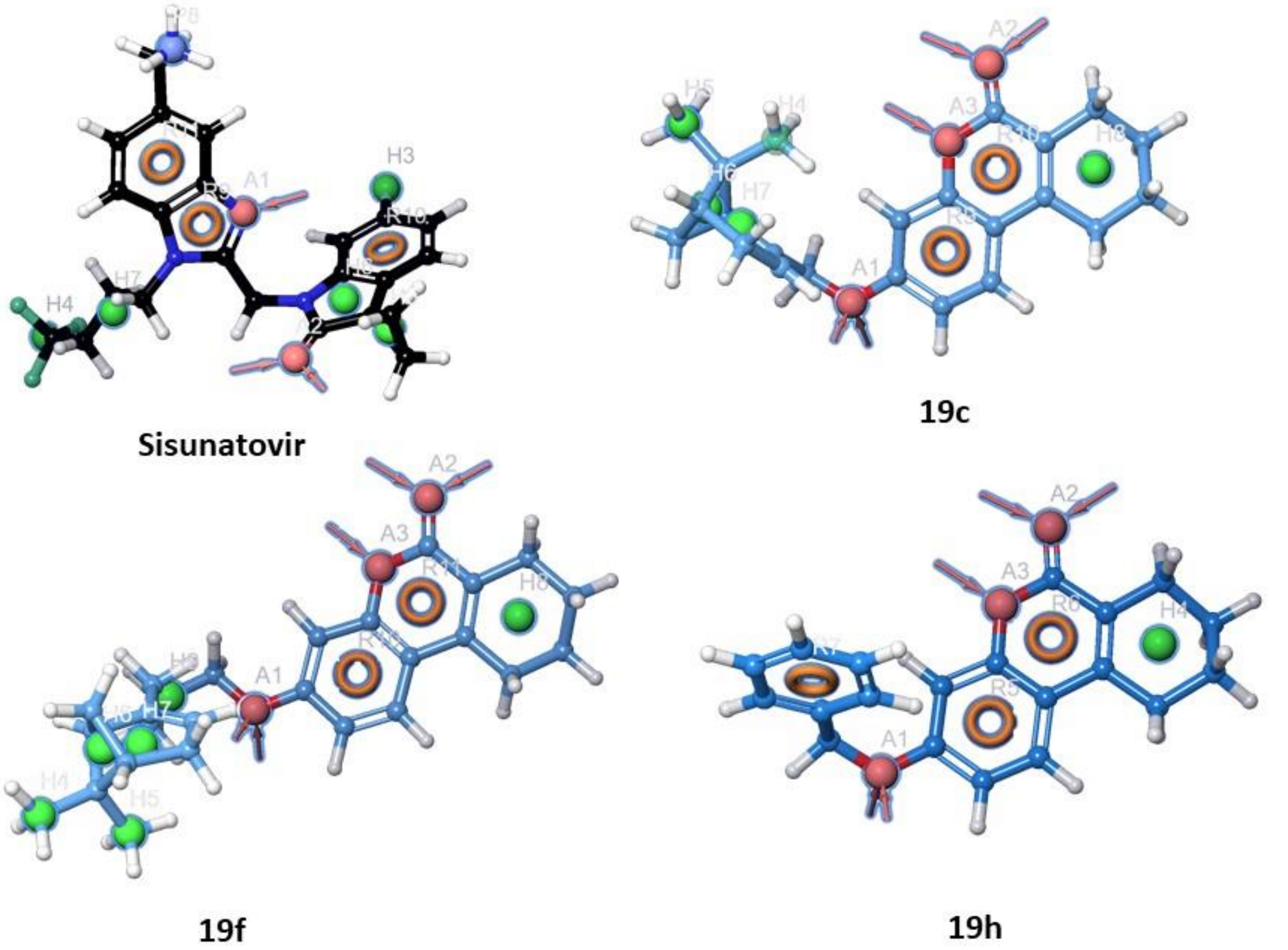

Figure S5 – the pharmacophore features of known F-protein inhibitor of sisunatovir and compounds **19c**, **19f**, and **19h**

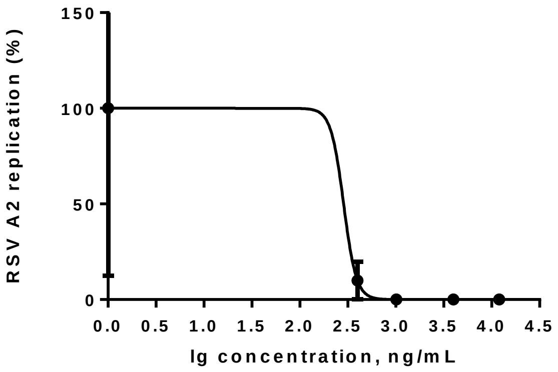

17c

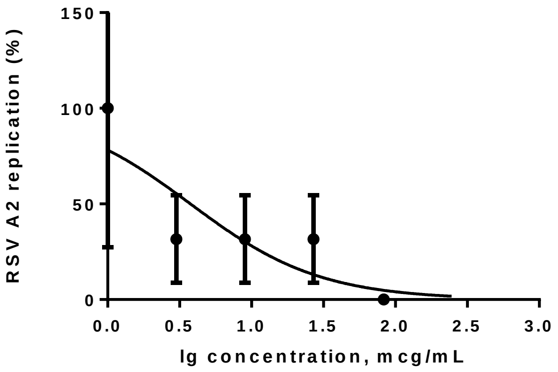

17f

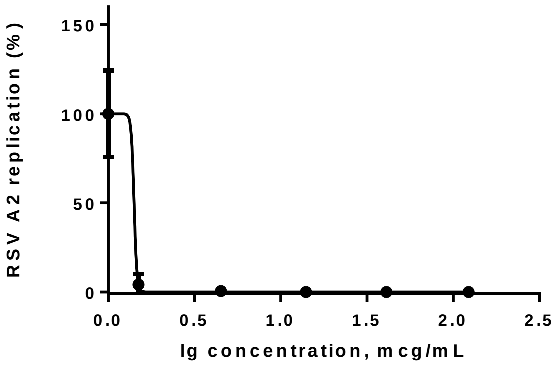

18c

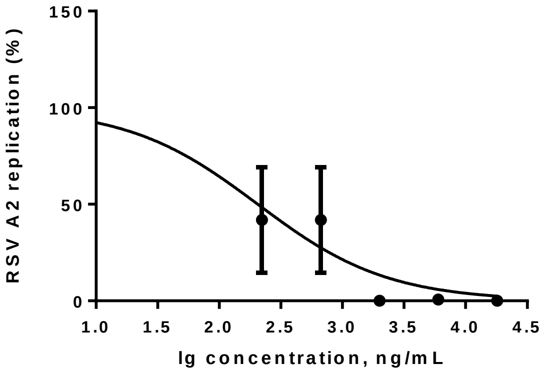

19a

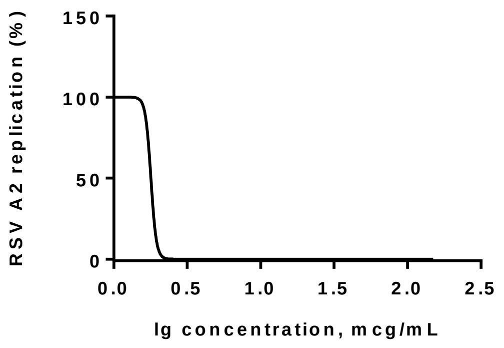

19c

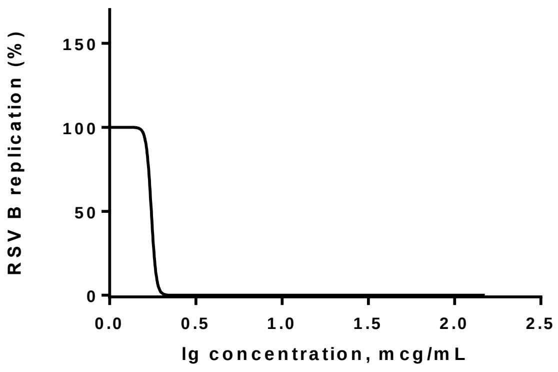

19c

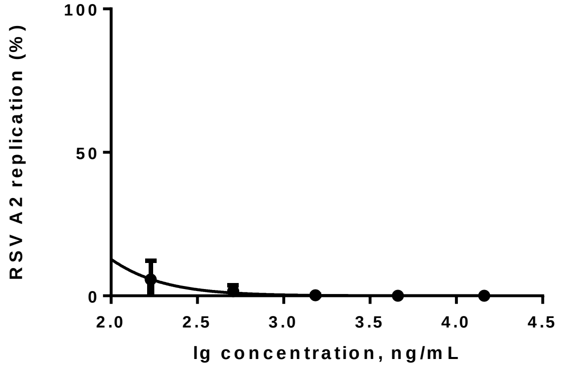

19d

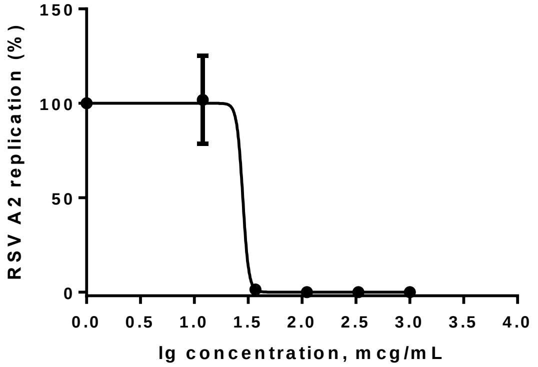

Ribavirin

Figure S6 – Dose-response curve and half maximal inhibitory concentration (IC_{50}) values of active compounds in Hep-2 cells against RSV A and B
